# Machine learning and Shapley Additive exPlanations to predict metastasis of lymph nodes posterior to the recurrent laryngeal nerve in cN0 papillary thyroid carcinoma

**DOI:** 10.3389/fonc.2025.1673332

**Published:** 2026-01-07

**Authors:** Jing Zhou, Ben Li, Tao Sun, DaXue Li, Chun Huang, Han Gao, Jiahui Ren, Yuchen Zhuang, Song Xue, Qian Xiao, Lin Chun, Xinliang Su

**Affiliations:** 1 Department of Breast and Thyroid, Women and Children’s Hospital of Chongqing Medical University, Chongqing Health Center for Women and Children, Chongqing, China; 2 Department of Chongqing Key Laboratory of Molecular Oncology and Epigenetics, The First Affiliated Hospital of Chongqing Medical University, Chongqing, China; 3 Department of Breast and Thyroid Surgery, The First Affiliated Hospital of Chongqing Medical University, Chongqing, China; 4 Intelligent Integrated Circuits and Systems Laboratory (SICS Lab), University of Electronic Science and Technology of China, Chengdu, Sichuan, China; 5 Department of Breast and Thyroid Surgery, Guangyuan Central Hospital, Guangyuan, Sichuan, China

**Keywords:** clinically negative neck lymph nodes (cN0), papillary thyroid carcinoma (PTC), lymph nodes posterior to the recurrent laryngeal nerve (LN-prRLN), Extreme Gradient Boosting (XGBoost), SHapley Additive exPlanations (SHAP)

## Abstract

**Objective:**

Prophylactic dissection of lymph nodes posterior to the recurrent laryngeal nerve (LN-prRLN) in clinically node-negative (cN0) papillary thyroid carcinoma (PTC) remains controversial due to the inability to preoperatively assess LN-prRLN metastasis.

**Materials and methods:**

This study aims to construct and validate an interpretable predictive model for LN-prRLN metastasis in cN0 PTC using machine learning (ML) method. Data were collected from hospital A and divided into training and testing sets (7:3). Additional data from the hospital B were used as validation set. Nine ML models, including XGBoost, were developed. Predictive performance was evaluated using ROC curves, decision curve analysis (DCA), calibration curves, and precision-recall curves. The best model was compared to a traditional logistic regression-based nomogram using learning curves and the method of Probability-based Ranking Model Approach (PMRA). SHapley Additive exPlanations (SHAP) were used to interpret the top ten predictive features and create a web-based calculator.

**Results:**

A total of 2033 patients were included. XGBoost outperformed other models with AUCs of 0.859, and 0.885 for the testing, and validation sets, respectively, compared to the nomogram (0.814, 0.836). SHAP-based visualizations identified the top ten predictive features: ipsilateral paratracheal lymph node metastasis rate, number of total central lymph node metastases, total central lymph node metastasis rate, number of ipsilateral paratracheal lymph node metastases, pretracheal lymph node metastasis rate, ipsilateral paratracheal lymph node metastasis, unclear tumor border, size, and age ≤39 years. These features were used to develop a web-based calculator.

**Conclusion:**

ML is a reliable tool for predicting LN-prRLN metastasis in cN0 PTC patients. The SHAP method provides insights into the XGBoost model, and the resultant web-based calculator is a clinically useful tool to assist in the surgical planning for LN-prRLN dissection.

## 1 Introduction

Papillary thyroid carcinoma (PTC) is the most common subtype of differentiated thyroid carcinoma (DTC), accounting for approximately 85% of thyroid cancers (1–4). Despite its indolent nature, lymph node metastasis (LNM) occurs in 20-90% of PTC patients, leading to local recurrence and distant metastasis (2, 5, 6). Anatomically, the right recurrent laryngeal nerve (RLN) branches from the first segment of the subclavian artery and divides the right paratracheal lymph nodes into LN-prRLN and anterior RLN lymph nodes (LN-arRLN) (6–8).

Prophylactic central lymph node dissection (CLND) is recommended for high-risk PTC patients (T3 or T4) (2, 9, 10), But is controversial for cN0 patients due to the low LN-prRLN metastasis rate (2.7%-22.4%) (11–13). Extensive dissection increases the risk of complications such as RLN injury, parathyroid damage, or chyle leakage (14–16).However, occult LN-prRLN metastasis, the situation of central compartment without metastasis, but with LN-prRLN metastasis, has been reported in 2.3%-5.86% of cases (17–19). It may cause recurrence, and reoperation can lead to severe complications like permanent vocal cord paralysis or hypoparathyroidism (5, 20). Accurately predicting LN-prRLN metastasis could optimize surgical planning, minimizing unnecessary dissections and associated complications (8, 21, 22).

Although ultrasound is commonly used for examination (4, 12, 23, 24), its accuracy is limited (44.4%-65.8%) due to the deep anatomical location of LN-prRLN and the presence of micrometastases in cN0 patients (25). Multimodal prediction models incorporating clinical, ultrasound, and intraoperative frozen pathology data have improved accuracy but often rely on linear models such as logistic regression or LASSO (11, 21, 26), which may have selection bias and overfitting issues (19, 21, 27), could not allowed clinicians to make preliminary assessments of the likelihood of LN-prRLN metastasis prior to surgery (25).

Machine learning (ML) has shown promise in medical applications, addressing the limitations of linear models by handling large (28–30), diverse datasets through various algorithms (29, 31, 32). This study develops a new ML-based scoring system to predict LN-prRLN metastasis in cN0 PTC patients, comparing traditional models with various ML models, visualizing predictive results, and creating a web-based calculator for clinical decision support.

## 2 Materials and methods

### 2.1 Patients

This retrospective study was approved by the Ethics Committee of the First Affiliated Hospital of Chongqing Medical University (Approval No. 2020-181). All methods of this study were performed in accordance with the principles outlined in the Declaration of Helsinki. Clinical records of 4185 PTC patients treated at the First Affiliated Hospital of Chongqing Medical University (Hospital A) from 2016 to 2020 were retrospectively analyzed. Additionally, clinical and pathological data of 651 PTC patients treated at the Women and Children’s Hospital of Chongqing Medical University (Hospital B) from 2018 to 2020 were collected. Inclusion criteria were: age above 18 years; diagnosis of thyroid cancer; complete case data and regional lymph node analysis; no history of previous neck surgery or radiation; no preoperative diagnosis of cN1. Exclusion criteria included: previous neck surgery or radiation; age below 18 years; other types of thyroid malignancies; incomplete case data or lack of regional lymph node analysis; preoperative diagnosis of cN1 (1, 2). Strict adherence to inclusion and exclusion criteria resulted in 1714 PTC patients in the medical cohort and 319 PTC patients in the surgical cohort. Data preprocessing steps for the training, testing and validation sets included: 1. Handling missing values using mode imputation. 2. Categorizing age and tumor diameter into categorical variables using optimal cutoff values determined by ROC curve analysis.

**Figure 1:**
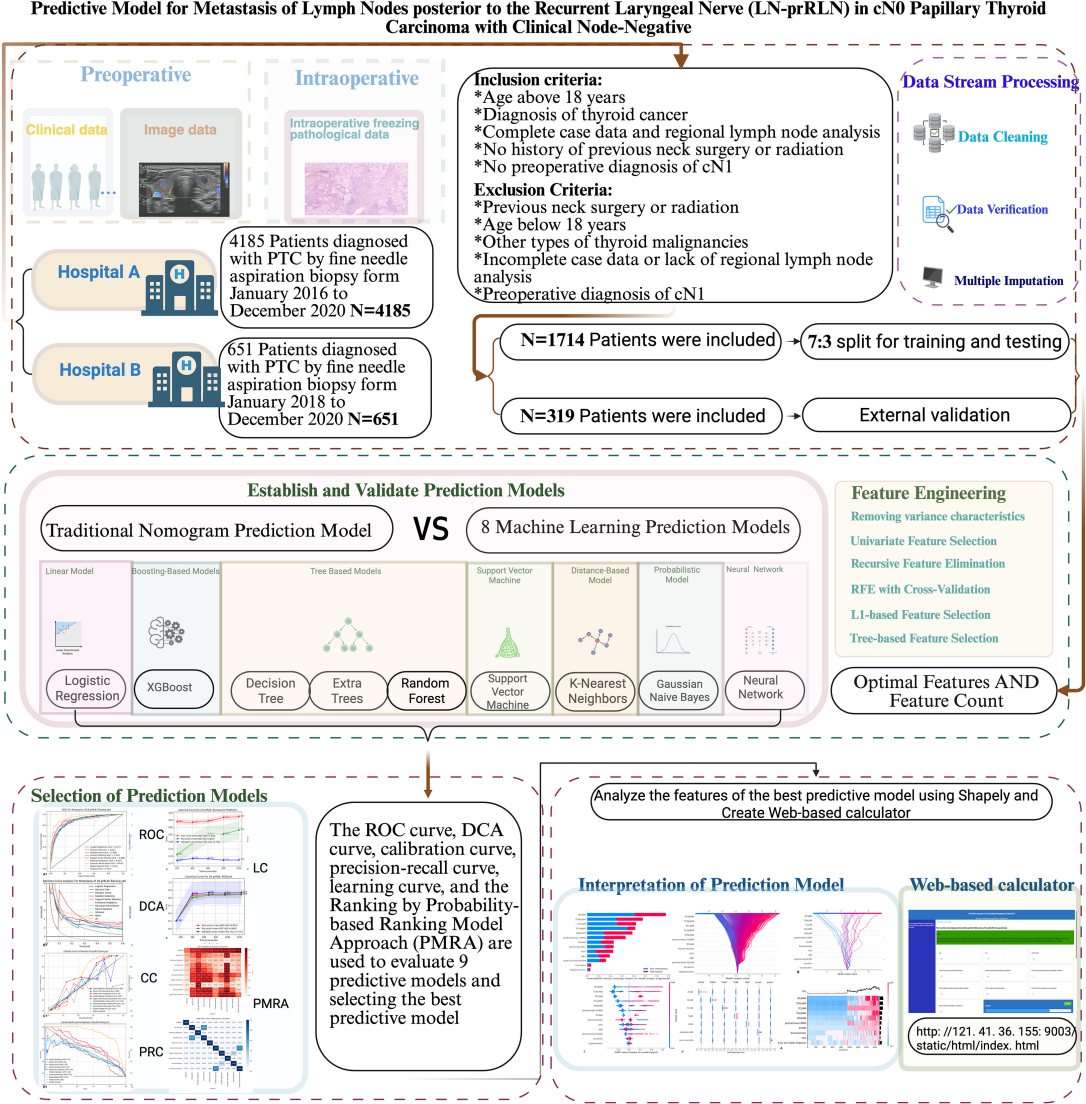
Workflow of the predictive model for metastasis of lymph nodes posterior to the recurrent laryngeal nerve (LN-prRLN) in papillary thyroid carcinoma with clinical node-negative (cN0) classification.

Standardizing and normalizing numerical data to improve data quality and reduce feature disparities. All patients were divided into LN-prRLN metastasis group (LN-prRLN positive group) and non-metastasis group (LN-prRLN negative group) based on postoperative pathological examination results. The medical cohort was randomly divided into training and testing sets (internal validation set) in a 7:3 ratio. The entire surgical cohort was used as the validation set (external validation set). Overall balance of each dataset was verified using chi-square tests (**Figure 1**; **Supplementary Material 1**).

### 2.2 Routine feature variables selection

Clinical Features: Age, sex, BMI. The optimal cutoff value for age determined by ROC curve analysis was 39 years. BMI was categorized according to Chinese and WHO standards: BMI < 18.5: underweight; 18. 5 ≤ BMI < 24: normal weight; BMI ≥ 24: overweight. Ultrasound Features: Collected ultrasound image parameters included tumor boundary (extra-thyroidal extension, irregular/lobulated, smooth/no boundary), aspect ratio (≤1, >1), composition (cystic/spongiform, mixed cystic and solid, solid), internal echo pattern (anechoic, hyperechoic/isoechoic, hypoechoic, very hypoechoic), internal echo homogeneity (homogeneous, heterogeneous), echogenic foci (none/comet-tail artifact, coarse calcifications, peripheral calcifications, microcalcifications), peripheral blood flow (none, abundant), internal tumor vascularization (none, abundant), tumor size defined as the maximum diameter, and location (upper, middle, lower, multiple sites, isthmus). All parameters were measured twice by three radiologists with 10, 15, and 17 years of experience, respectively, and the results were averaged. Cases were re-tested a week later for validation. Pathological features: Extrathyroidal extension (ETE) was defined as tumor invasion beyond the thyroid capsule, including gross ETE (invasion into prethyroid muscles) and maximal ETE (invasion into the trachea, larynx, or recurrent laryngeal nerve). Hashimoto’s thyroiditis was diagnosed based on any of the following criteria: (i) thyroid peroxidase antibody levels >50 IU/mL, (ii) diffuse heterogeneity on ultrasound, (iii) diffuse lymphocytic infiltration on histopathology. Multifocality was confirmed via ultrasound and intraoperative frozen section. Tumor staging was based on intraoperative frozen pathology results (**Table 1**).

**Table 1:** Clinical features, ultrasound image features, and intraoperative frozen section pathology features across training, testing and validation sets.

Characteristics	Total(N = 1714)		Univariate	Training set(N = 1200)		Univariate	Testing set(N = 514)		Univariate	Validation set(N = 319)		Univariate
	Metastasis of LN-prRLN(-) 1471(85.823%)	Metastasis of LN-prRLN(+) 243(14.177%)	P-value	Metastasis of LN-prRL(-)1021(85.083%)	Metastasis of LN-prRLN(+)179(14.917%)	P-value	Metastasis of LN-prRLN(-) 450(87.549%)	Metastasis of LN-prRLN(+) 64(12.451%)	P-value	Metastasis of LN-prRLN(-) 278(87.147%)	Metastasis of LN-prRLN(+) 41(12.853%)	P-value
Age	43 ± 11.7	40.2 ± 11.4	<0.001	42.986 ± 11.769	39.682 ± 11.631	0.001	42.993 ± 11.581	41.828 ± 10.512	0.447	42.155 ± 11.173	35.512 ± 10.332	0
age>39	876 (59.6%)	117 (48.1%)	0.001	604 (59.158%)	81 (45.251%)	0.001	272 (60.444%)	36 (56.250%)	0.614	146 (52.518%)	10 (24.390%)	0.001
age ≤ 39	595 (40.4%)	126 (51.9%)		417 (40.842%)	98 (54.749%)		178 (39.556%)	28 (43.750%)		132 (47.482%)	31 (75.610%)	
Sex												
Female	1095 (74.4%)	155 (63.8%)	<0.001	774 (75.808%)	112 (62.570%)	<0.001	321 (71.333%)	43 (67.188%)	0.592	242 (87.050%)	38 (92.683%)	0.44
Male	376 (25.6%)	88 (36.2%)		247 (24.192%)	67 (37.430%)		129 (28.667%)	21 (32.812%)		36 (12.950%)	3 (7.317%)	
BMI	23.200 ± 3.220	23.400 ± 3.410	0.411	23.249 ± 3.200	23.602 ± 3.555	0.18	23.185 ± 3.275	22.920 ± 2.951	0.539	23.533 ± 3.256	24.155 ± 4.251	0.274
Normal	859 (58.4%)	147 (60.5%)	0.559	597 (58.472%)	104 (58.101%)	0.784	262 (58.222%)	43 (67.188%)	0.356	155 (55.755%)	18 (43.902%)	0.338
Overweight	542 (36.8%)	88 (36.2%)		379 (37.120%)	69 (38.547%)		163 (36.222%)	19 (29.688%)		110 (39.568%)	20 (48.780%)	
Underweight	70 (4.8%)	8 (3.3%)		45 (4.407%)	6 (3.352%)		(25 (5.556%)	2 (3.125%)		13 (4.676%)	3 (7.317%)	
Tumor.border												
extrandular-invasion	644 (43.8%)	43 (17.7%)	<0.001	72 (7.052%)	67 (37.430%)	<0.001	36 (8.000%)	19 (29.688%)	<0.001	15 (5.396%)	2 (4.878%)	0.396
irregular-shape/lsharpobed	719 (48.9%)	114 (46.9%)		500 (48.972%)	82 (45.810%)		219 (48.667%)	32 (50.000%)		230 (82.734%)	37 (90.244%)	
smooth/borderless	108 (7.3%)	86 (35.4%)		449 (43.976%)	30 (16.760%)		195 (43.333%)	13 (20.312%)		33 (11.871%)	2 (4.878%)	
Aspect.ratio												
≤1	752 (51.1%)	112 (46.1%)	0.166	532 (52.106%)	83 (46.369%)	0.182	220 (48.889%)	29 (45.312%)	0.688	90 (32.374%)	17 (41.463%)	0.33
>1	719 (48.9%)	131 (53.9%)		489 (47.894%)	96 (53.631%)		230 (51.111%)	35 (54.688%)		188 (67.626%)	24 (58.537%)	
Ingredients												
cystic/cavernous	8 (0.5%)	0 (0%)	0.489	7 (0.686%)	0(0%)	0.492	1 (0.222%)	0(0%)	0.387	1 (0.360%)		0.528
Mixed cystic and solid	35 (2.4%)	5 (2.1%)		23 (2.253%)	5 (2.793%)		12 (2.667%)	0(0%)		2 (0.719%)	1 (2.439%)	
solid	1428 (97.1%)	238 (97.9%)		991 (97.062%)	174 (97.207%)		437 (97.111%)	64 (100.000%)		275 (98.921%)	40 (97.561%)	
Internal.echo.pattern												
echoless	3 (0.2%)	0 (0%)	0.119	1 (0.098%)	0 (0%)	0.215	2 (0.444%)	0(0%)	0.446	1 (0.360%)		0.71
high/isoechoic	169 (11.5%)	18 (7.4%)		118 (11.557%)	12 (6.704%)		51 (11.333%)	6 (9.375%)		23 (8.273%)	5 (12.195%)	
hypoechoic	1247 (84.8%)	220 (90.5%)		864 (84.623%)	162 (90.503%)		383 (85.111%)	58 (90.625%)		250 (89.928%)	36 (87.805%)	
very hypoechoic	52 (3.5%)	5 (2.1%)		38 (3.722%)	5 (2.793%)		4 (3.111%)	0(0%)		4 (1.439%)		
Internal.echo.homogeneous												
Uniform	593 (40.3%)	89 (36.6%)	0.309	602 (58.962%)	116 (64.804%)	0.165	276 (61.333%)	38 (59.375%)	0.87	184 (66.187%)	28 (68.293%)	0.929
Non-uniform	878 (59.7%)	154 (63.4%)		419 (41.038%)	63 (35.196%)		174 (38.667%)	26 (40.625%)		94 (33.813%)	13 (31.707%)	
Hyperechoic												
no/large comet tail	378 (25.7%)	52 (21.4%)	0.478	516 (50.539%)	34 (18.994%)	0.112	112 (24.889%)	18 (28.125%)	0.039	67 (24.101%)	4 (9.756%)	0.085
coarse calcification	279 (19.0%)	50 (20.6%)		207 (20.274%)	32 (17.877%)		72 (16.000%)	18 (28.125%)		19 (6.835%)	2 (4.878%)	
peripheral calcification	45 (3.1%)	6 (2.5%)		32 (3.134%)	6 (3.352%)		13 (2.889%)	0(0%)		4 (1.439%)	2 (4.878%)	
Microcalcification	769 (52.3%)	135 (55.6%)		516 (50.539%)	107 (59.777%)		253 (56.222%)	28 (43.750%)		188 (67.626%)	33 (80.488%)	
Tumor.Peripheral.blood.flow												
Without	1254 (85.2%)	141 (58.0%)	<0.001	869 (85.113%)	102 (56.983%)	<0.001	379 (84.222%)	38 (59.375%)	<0.001	129 (46.403%)	13 (31.707%)	0.11
Abundant	217 (14.8%)	102 (42.0%)		152 (14.887%)	77 (43.017%)		71 (15.778%)	26 (40.625%)		149 (53.597%)	28 (68.293%)	
Tumor.internal.vascularization												
Without	1248 (84.8%)	140 (57.6%)	<0.001	875 (85.700%)	102 (56.983%)	<0.001	379 (84.222%)	39 (60.938%)	<0.001	158 (56.835%)	13 (31.707%)	0.004
Abundant	223 (15.2%)	103 (42.4%)		146 (14.300%)	77 (43.017%)		71 (15.778%)	25 (39.062%)		120 (43.165%)	28 (68.293%)	
Size	11.100 ± 7.640	15.600 ± 10.100	<0.001	11.219 ± 7.886	16.022 ± 10.500	<0.001	10.823 ± 7.051	14.523 ± 8.798	<0.001	8.950 ± 5.926	12.005 ± 7.095	0.003
>10	940 (63.9%)	102 (42.0%)	<0.001	374 (36.631%)	108 (60.335%)	<0.001	157 (34.889%)	33 (51.562%)	0.014	87 (31.295%)	19 (46.341%)	0.083
≤10	531 (36.1%)	141 (58.0%)		647 (63.369%)	71 (39.665%)		293 (65.111%)	31 (48.438%)		191 (68.705%)	22 (53.659%)	
Location												
Upper	271 (18.4%)	50 (20.6%)	0.334	190 (18.609%)	32 (17.877%)	0.491	81 (18.000%)	18 (28.125%)	0.229	34 (12.230%)	5 (12.195%)	0.171
Middle	571 (38.8%)	94 (38.7%)		386 (37.806%)	73 (40.782%)		185 (41.111%)	21 (32.812%)		102 (36.691%)	9 (21.951%)	
Under	413 (28.1%)	69 (28.4%)		293 (28.697%)	50 (27.933%)		120 (26.667%)	19 (29.688%)		62 (22.302%)	16 (39.024%)	
Multisite	192 (13.1%)	30 (12.3%)		135 (13.222%)	24 (13.408%)		57 (12.667%)	6 (9.375%)		74 (26.619%)	10 (24.390%)	
Isthmus	24 (1.6%)	0 (0%)		17 (1.665%)	0(0%)		7 (1.556%)	0(0%)		6 (2.158%)	1 (2.439%)	
ETE												
Without	1335 (90.8%)	158 (65.0%)	<0.001	931 (91.185%)	112 (62.570%)	<0.001	404 (89.778%)	46 (71.875%)	<0.001	241 (86.691%)	32 (78.049%)	0.218
Abundant	136 (9.2%)	85 (35.0%)		90 (8.815%)	67 (37.430%)		46 (10.222%)	18 (28.125%)		37 (13.309%)	9 (21.951%)	
Mulifocality												
Without	1141 (77.6%)	156 (64.2%)	<0.001	785 (76.885%)	115 (64.246%)	<0.001	356 (79.111%)	41 (64.062%)	0.011	180 (64.748%)	21 (51.220%)	0.133
Abundant	330 (22.4%)	87 (35.8%)		236 (23.115%)	64 (35.754%)		94 (20.889%)	23 (35.938%)		98 (35.252%)	20 (48.780%)	
Hashimoto												
Without	1174 (79.8%)	191 (78.6%)	0.728	814 (79.726%)	145 (81.006%)	0.769	360 (80.000%)	46 (71.875%)	0.184	203 (73.022%)	24 (58.537%)	0.084
Abundant	297 (20.2%)	52 (21.4%)		207 (20.274%)	34 (18.994%)		90 (20.000%)	18 (28.125%)		75 (26.978%)	17 (41.463%)	
T.staging												
1	1222 (83.1%)	127 (52.3%)	<0.001	852 (83.448%)	89 (49.721%)	<0.001	370 (82.222%)	38 (59.375%)	<0.001	235 (84.532%)	26 (63.415%)	0.01
2	109 (7.4%)	26 (10.7%)		73 (7.150%)	21 (11.732%)		36 (8.000%)	5 (7.812%)		9 (3.237%)	4 (9.756%)	
3	107 (7.3%)	70 (28.8%)		72 (7.052%)	54 (30.168%)		35 (7.778%)	16 (25.000%)		26 (9.353%)	9 (21.951%)	
4	33 (2.2%)	20 (8.2%)		24 (2.351%)	15 (8.380%)		9 (2.000%)	5 (7.812%)		8 (2.878%)	2 (4.878%)	
prelaryngeal.LNM												
No	1186 (80.6%)	116 (47.7%)	<0.001	830 (81.293%))	84 (46.927%)	<0.001	356 (79.111%)	32 (50.000%)	<0.001	209 (75.180%)	24 (58.537%)	0.027
Yes	143 (9.7%)	99 (40.7%)		102 (9.990%)	73 (40.782%)		41 (9.111%)	26 (40.625%)		33 (11.871%)	11 (26.829%)	
	142 (9.7%)	28 (11.5%)		89 (8.717%)	22 (12.291%)		53 (11.778%)	6 (9.375%)		36 (12.950%)	6 (14.634%)	
prelaryngeal.LNMR												
Mean ± SD	0.074 ± 0.234	0.339 ± 0.419	<0.001	0.075 ± 0.236	0.355 ± 0.431	<0.001	0.072 ± 0.232	0.295 ± 0.385	<0.001	0.069 ± 0.206	0.186 ± 0.335	0.005
prelaryngeal.NLNM												
Mean ± SD	0.163 ± 0.566	0.698 ± 0.936	<0.001	0.171 ± 0.584	0.682 ± 0.877	<0.001	0.146 ± 0.521	0.741 ± 1.085	<0.001	0.182 ± 0.499	0.400 ± 0.651	0.021
pretracheal.LNM												
No	1090 (74.1%)	63 (25.9%)	<0.001	762 (74.633%)	45 (25.140%)	<0.001	328 (72.889%)	18 (28.125%)	<0.001	201 (72.302%)	9 (21.951%)	<0.001
Yes	371 (25.2%)	179 (73.7%)		254 (24.878%)	134 (74.860%)		117 (26.000%)	45 (70.312%)		77 (27.698%)	32 (78.049%)	
	10 (0.7%)	1 (0.4%)		5 (0.490%)	0(0%)		5 (1.111%)	1 (1.562%)		0(0%)	0(0%)	
pretracheal.LNMR												
Mean ± SD	0.128 ± 0.263	0.471 ± 0.387	<0.001	0.125 ± 0.261	0.486 ± 0.392	<0.001	0.133 ± 0.266	0.430 ± 0.374	<0.001	0.155 ± 0.295	0.457 ± 0.344	<0.001
pretracheal.NLNM												
Mean ± SD	0.463 ± 0.971	1.89 ± 2.23	<0.001	0.464 ± 0.984	1.883 ± 2.188	<0.001	0.461 ± 0.940	1.905 ± 2.374	<0.001	0.514 ± 1.015	1.878 ± 1.364	<0.001
IPLNM												
No	1111 (75.5%)	49 (20.2%)	<0.001	775 (75.906%)	35 (19.553%)	<0.001	336 (74.667%)	14 (21.875%)	<0.001	201 (72.302%)	2 (4.878%)	<0.001
Yes	360 (24.5%)	194 (79.8%)		246 (24.094%)	144 (80.447%)		114 (25.333%)	50 (78.125%)		77 (27.698%)	39 (95.122%)	
IPLNMR												
Mean ± SD	0.134 ± 0.278	0.595 ± 0.39	<0.001	0.130 ± 0.271	0.594 ± 0.390	<0.001	0.145 ± 0.294	0.595 ± 0.394	<0.001	0.103 ± 0.191	0.445 ± 0.203	<0.001
IPNLNM												
Mean ± SD	0.432 ± 0.947	1.99 ± 1.94	<0.001	0.430 ± 0.943	1.877 ± 1.708	<0.001	0.436 ± 0.959	2.312 ± 2.474		0.482 ± 1.057	2.049 ± 1.161	<0.001
TCLNM												
No	927 (63.0%)	19 (7.8%)	<0.001	649 (63.565%)	15 (8.380%)	<0.001	278 (61.778%)	4 (6.250%)	<0.001	160 (57.554%)	0(0%)	<0.001
Yes	544 (37.0%)	224 (92.2%)		372 (36.435%)	164 (91.620%)		172 (38.222%)	60 (93.750%)		118 (42.446%)	41 (100.000%)	
TCLNMR												
Mean ± SD	0.119 ± 0.202	0.492 ± 0.301	<0.001	0.130 ± 0.271	0.594 ± 0.390	<0.001	0.124 ± 0.215	0.470 ± 0.292	<0.001	0.117 ± 0.177	0.420 ± 0.168	<0.001
TCNLNM												
Mean ± SD	1.04 ± 1.84	4.49 ± 3.66	<0.001	1.047 ± 1.859	4.358 ± 3.381	<0.001	1.020 ± 1.794	4.859 ± 4.342	<0.001	1.155 ± 1.898	4.268 ± 1.924	<0.001

LN-prRLN, Lymph nodes posterior to the recurrent laryngeal nerve; ETE, Extrathyroidal Extension; LNM, Lymph Node Metastasis; LNMR, Lymph Node Metastasis Ratio; SD, Standard Deviation; NLNM, Number of Lymph Nodes Metastasis; IPLNM, Ipsilateral paratracheal lymph node metastasis; IPLNMR, Ratio of Ipsilateral Paratracheal Lymph Node Metastasis; IPNLNM, Number of Ipsilateral Paratracheal Lymph Node Metastasis; TCLNM, Total Central Lymph Node Metastasis; TCLNMR, Ratio of Total Central Lymph Node Metastasis: TCNLNM, Number of Total Central Lymph Node Metastasis.

### 2.3 Surgical procedure and intraoperative frozen section variables

LN-prRLN were identified according to standardized anatomical landmarks as previously described (13, 18). The recurrent laryngeal nerve was traced from its laryngeal entry point caudally to its vagal origin (33, 34). LN-prRLN were defined as lymph nodes in the tissue plane immediately posterior to the recurrent laryngeal nerve, bounded anteriorly by the nerve, posteriorly by the prevertebral fascia, medially by the tracheoesophageal groove, and laterally by the carotid sheath (35, 36). During dissection, LN-prRLN were carefully separated from adjacent nodal stations using meticulous technique while preserving nerve integrity. Each specimen was individually harvested and placed in separate labeled containers indicating “Lymph nodes posterior to recurrent laryngeal nerve “ with detailed anatomical descriptions. All specimens were submitted to pathology with standardized labeling protocols, and a quality assurance protocol requiring cross-verification between surgical notes and pathological reports ensured 100% concordance in nodal station identification.

Surgical procedures: For right-sided PTC patients with tumors <4.0 cm, we routinely performed lobectomy and isthmectomy with ipsilateral CLND. If intraoperative frozen section examination detected LNM, total thyroidectomy was performed. For bilateral PTC, we performed total thyroidectomy with bilateral CLND. In the right paratracheal region, LN-arRLN were labeled as ipsilateral paratracheal lymph nodes to distinguish them from LN-prRLN. Total central lymph nodes (TCLN), excluding LN-prRLN, referred to prelaryngeal, pretracheal, and paratracheal lymph nodes collectively. The surgical procedure has been previously described in our studies (19, 28, 30). surgical specimens were categorized into four subgroups: prelaryngeal, pretracheal, paratracheal, and LN-prRLN, sequentially dissected and labeled, then sent for immediate intraoperative frozen section examination. Postoperative histopathological examination was independently conducted by three pathologists. Intraoperative frozen pathology provided 14 variables of LNM status: prelaryngeal LNM, pretracheal LNM, ipsilateral paratracheal LNM, and TCLNM were binary variables; prelaryngeal, pretracheal, ipsilateral paratracheal, and TCLNM numbers were continuous variables including micro metastases and macro metastases; ratios of prelaryngeal, pretracheal, ipsilateral paratracheal, and TCLNM were calculated as the number of metastatic lymph nodes divided by the total number of lymph nodes dissected in each region, also as continuous variables without cut-off value determination due to non-normal distribution.

### 2.4 Construction of traditional nomogram prediction model

**Figure 2:**
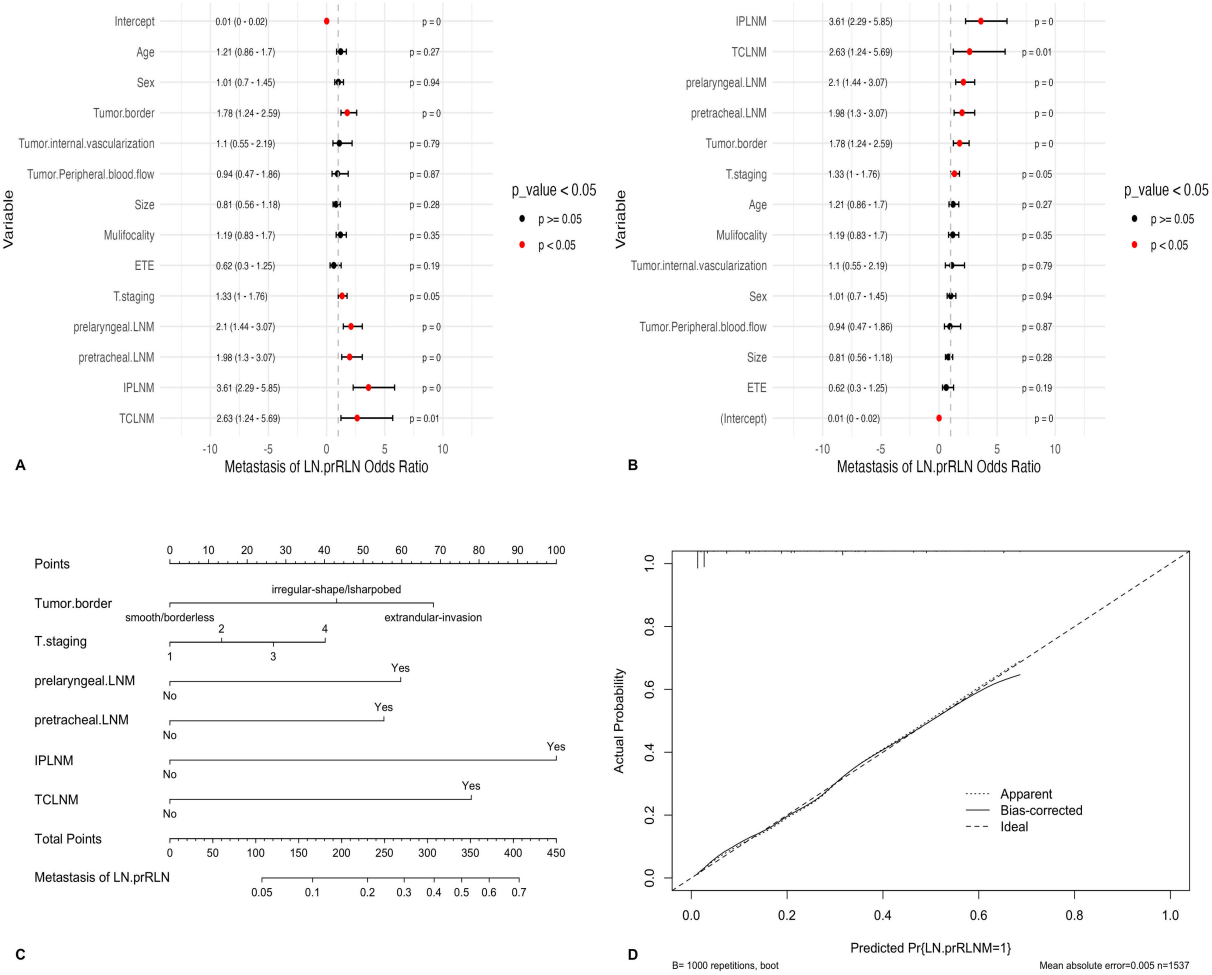
**(A)** Forest plot of binary logistic regression model, ordered by variable importance; **(B)** Forest plot of binary logistic regression model, ordered by odds ratio magnitude; **(C)** Traditional nomogram based on logistic regression; **(D)** Calibration curve of the nomogram; ETE, Extrathyroidal Extension; LNM, Lymph Node Metastasis; IPLNM, Ipsilateral Paratracheal Lymph Node Metastasis; TCLNM, Total Central Lymph Node Metastasis; LN-prRLN, Lymph Nodes posterior to the Recurrent Laryngeal Nerve.

Univariate analysis was conducted to screen feature variables, excluding those with poor correlation (**Table 1**). Feature variables with P-values < 0.05 in univariate analysis were included in multivariate logistic regression analysis, and a forest plot was generated (**Table 2**, **Figures 2A, B**). Independent risk factors identified through multivariate analysis were incorporated into the nomogram prediction model, drawn using the training set (**Figure 2C**). The goodness-of-fit was assessed using the Hosmer-Lemeshow method, comparing observed and predicted values, and displayed in a calibration curve (**Figure 2D**). The evaluation metrics of the traditional nomogram were subsequently compared with those of the ML prediction models.

**Table 2:** Multivariate logistic regression of factors associated with lymph node metastasis posterior to the recurrent laryngeal nerve.

Characteristics	P-value	OR(95%CI)
(Intercept)	0.000	0.009(0.005~0.018)
Age(≤39 years)	0.272	1.21(0.861~1.702)
Sex(Male)	0.942	1.013 (0.702~1.453)
Tumor.border(irregular-shape/lsharpobed)	0.002	1.776 (1.237~2.59)
Tumor.internal.vascularization(Abundant)	0.794	1.097 (0.548~2.195)
Tumor.Peripheral.blood.flow(Abundant)	0.866	0.943(0.471~1.86)
Size(>10mm)	0.278	0.812 (0.557~1.18)
Mulifocality(Yes)	0.351	1.187(0.825~1.699)
ETE(Yes)	0.187	0.619 (0.304~1.254)
T.staging(T3-T4)	0.046	1.332 (1.003~1.763)
prelaryngeal.LNM(Yes)	<0.001	2.101(1.439~3.072)
pretracheal.LNM(Yes)	0.002	1.983 (1.302~3.073)
IPLNM(Yes)	<0.001	3.605 (2.287~5.85)
TCLNM(Yes)	0.013	2.633 (1.236~5.69)

ETE, Extrathyroidal Extension; LNM, lymph node metastasis; IPLNM, Ipsilateral paratracheal lymph node metastasis; TCLNM, Total Central Lymph Node Metastasis.

Values highlighted in red indicate statistical significance at P < 0.05.

### 2.5 Establishment, optimization, and screening of ML prediction models

The architecture of the proposed ML models included four steps: 1. Feature variable selection; 2. Selection and training of ML prediction models; 3. Evaluation and tuning of nine prediction models; 4. Optimization and validation of models using testing and validation sets to identify the optimal prediction model.

A total of 31 feature variables were included. Feature selection methods varied for different prediction models (**Supplementary Material 2**). For logistic regression, variables were selected through univariate and multivariate analysis. Support vector machines used recursive feature elimination. Decision trees, random forests, gradient boosting, and extreme gradient boosting (XGBoost) used tree-based feature importance. Nearest neighbor algorithms applied recursive feature elimination with cross-validation. Gaussian Naive Bayes used correlation coefficient-based univariate feature selection. Neural networks employed L1 regularization-based model feature selection. The most relevant or important feature variables were incorporated into prediction models.

**Figure 3:**
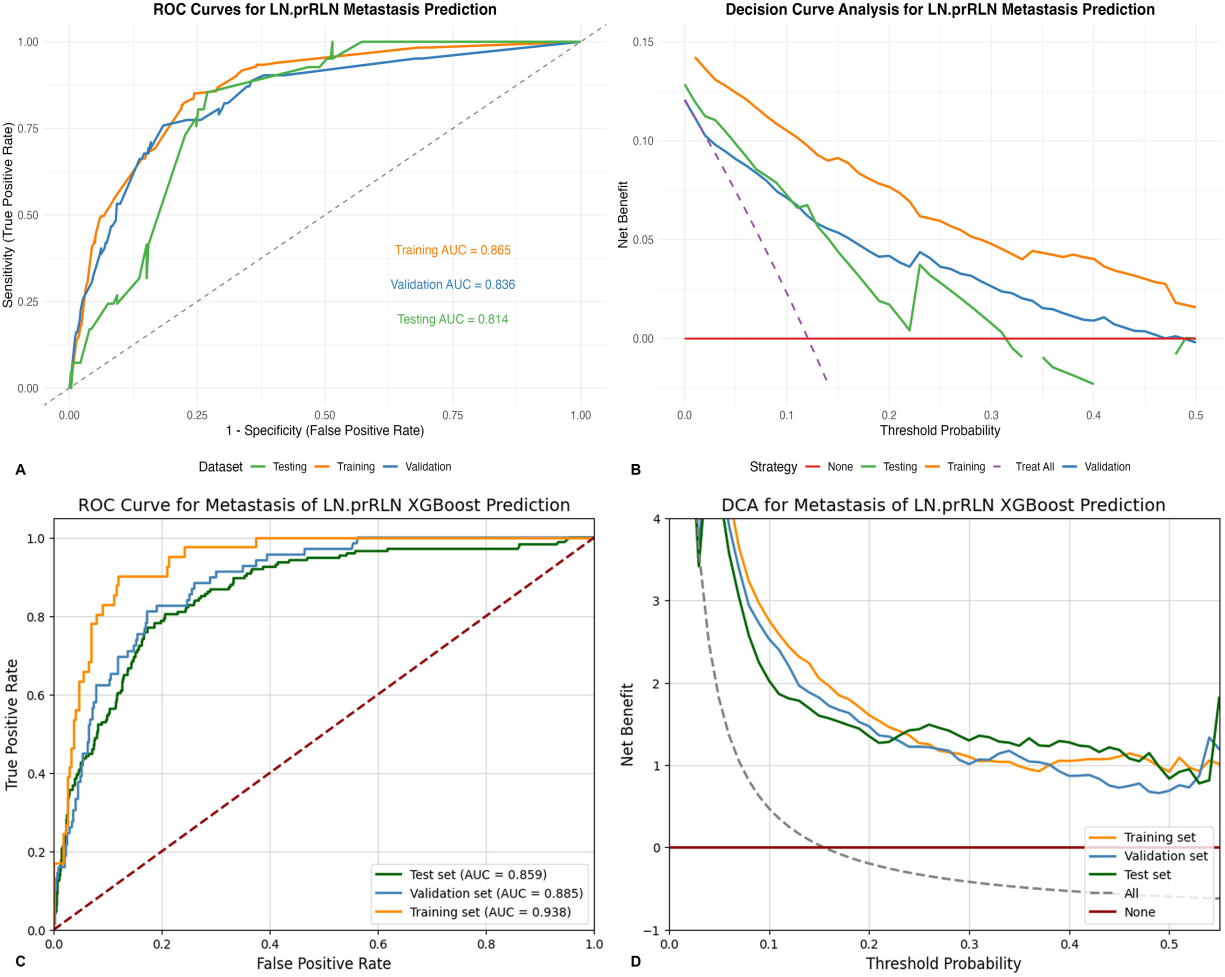
**(A)** ROC curves of the traditional predictive model on the training, testing and validation sets; **(B)** DCA curves of the traditional predictive model on the training, testing and validation sets; **(C)** ROC curves of the XGBoost predictive model on the training, testing and validation sets; **(D)** DCA curves of the XGBoost predictive model on the training, testing and validation sets; ROC, Receiver Operating Characteristic; DCA, Decision curve analysis; XGBoost, Extreme Gradient Boosting; LN-prRLN, Lymph Nodes posterior to the Recurrent Laryngeal Nerve.

Supervised learning was used for all ML prediction models, trained on the training set. Model tuning was performed using 10-fold cross-validation and grid search methods to optimize parameters and hyperparameters. Appropriate evaluation metrics were selected to assess model performance on training, testing and validation datasets. Evaluation metrics included ROC curves with AUC values for sensitivity and specificity trade-offs, decision curve analysis (DCA) curves for model utility assessment, calibration curves, and Brier scores for consistency between predicted probabilities and actual LNM rates. Precision-recall curves and area under precision-recall (AUPR) values assessed model performance at various prediction thresholds. Generalization ability and performance were evaluated using the testing set (internal validation) and validation set (external validation). Models were further tuned based on evaluation results, adjusting hyperparameters and adding regularization. Evaluation metrics and curves were recalculated for testing and validation sets to identify the optimal prediction model. Comparative ROC and DCA curves were plotted to assess the prediction value and utility of the optimal prediction model against traditional models (**Figure 3**).

### 2.6 Visualization and clinical application of the optimal model

Based on comprehensive evaluation metrics and assessment curves, the optimal prediction model and its parameter settings were identified: detailed parameter search range included ‘eta’: 0.027825594022071243, ‘n_estimators’: 333, ‘gamma’: 0.89, ‘max_depth’: 3, ‘min_child_weight’: 0, ‘colsample_bytree’: 0.3, ‘colsample_bylevel’: 0.0, ‘subsample’: 0.11111111111, ‘reg_lambda’: 0.2, ‘reg_alpha’: 0.Shapley Additive Explanations (SHAP) values for each feature variable in the prediction model were calculated, and a histogram of the top ten contributing feature variables was created. A web-based calculator was developed to visualize and apply the model in clinical settings.

### 2.7 Statistical analysis

Chi-square tests were used for analyzing binary, unordered multicategorical, and ordered multicategorical count data. Binary logistic regression analysis was employed to identify independent risk factors in metastasis of LN-prRLN. A nomogram prediction model was constructed based on statistically significant indicators from binary logistic regression analysis. Statistical analysis was conducted using R version 4.3.2 in R Studio (R Project for Statistical Computing). The “pROC” package was used for calculating optimal cut-off values for age and tumor diameter among categorical variables. The “foreign” and “rms” packages were used to create the nomogram prediction model and calibration curves. Python (version 3.11.5; Python Software Foundation, Wilmington, DE, USA) was used for variable selection, model training, and evaluation. The scikit-learn Python library (version 0.24) and the XGBoost package (version 1.7.3) were used to create and tune ML models. Performance metrics for evaluating classification performance were also derived using the scikit-learn Python library (version 0.24). The design code for this study using Python software can be obtained on GitHub (https://github.com/ZJ573693/ML-for-LN-prRLN).

## 3 Results

### 3.1 Basic characteristics

This study included 1200 patients (70%) in the training set, average age 42.5 ± 11.8 years (314 males, 886 females). The testing set comprised 514 patients (30%), average age 42.8 ± 11.5 years (150 males, 364 females). The validation set included 319 patients, average age 41.3 ± 11.3 years. Detailed ultrasound and clinicopathological characteristics are in **Supplementary Material 1**. Consistency analyses for continuous variables showed overall data balance (p>0. 05).

### 3.2 Establishment of traditional nomogram prediction model

Univariate analysis identified age ≤ 39 years, male gender, irregular tumor border, abundant tumor vascularization, tumor size > 10 mm, multifocality, ETE, T staging as T3-T4, prelaryngeal LNM, pretracheal LNM, IPLNM, and TCLNM as associated with LN-prRLN metastasis (**Table 1**, **Figure 2A**). Multivariate analysis confirmed irregular tumor border, T staging as T3-T4, prelaryngeal LNM, pretracheal LNM, IPLNM, and TCLNM as independent risk factors (**Table 2**, **Figures 2A, B**). These were included in the nomogram prediction model, showing good consistency (**Figures 2C, D**). ROC values for the training, testing and validation sets were 0.865, 0.814, and 0.836, respectively (**Figures 3A, B**).

### 3.3 Selection, optimization, and comparison of optimal ML model

**Figure 4:**
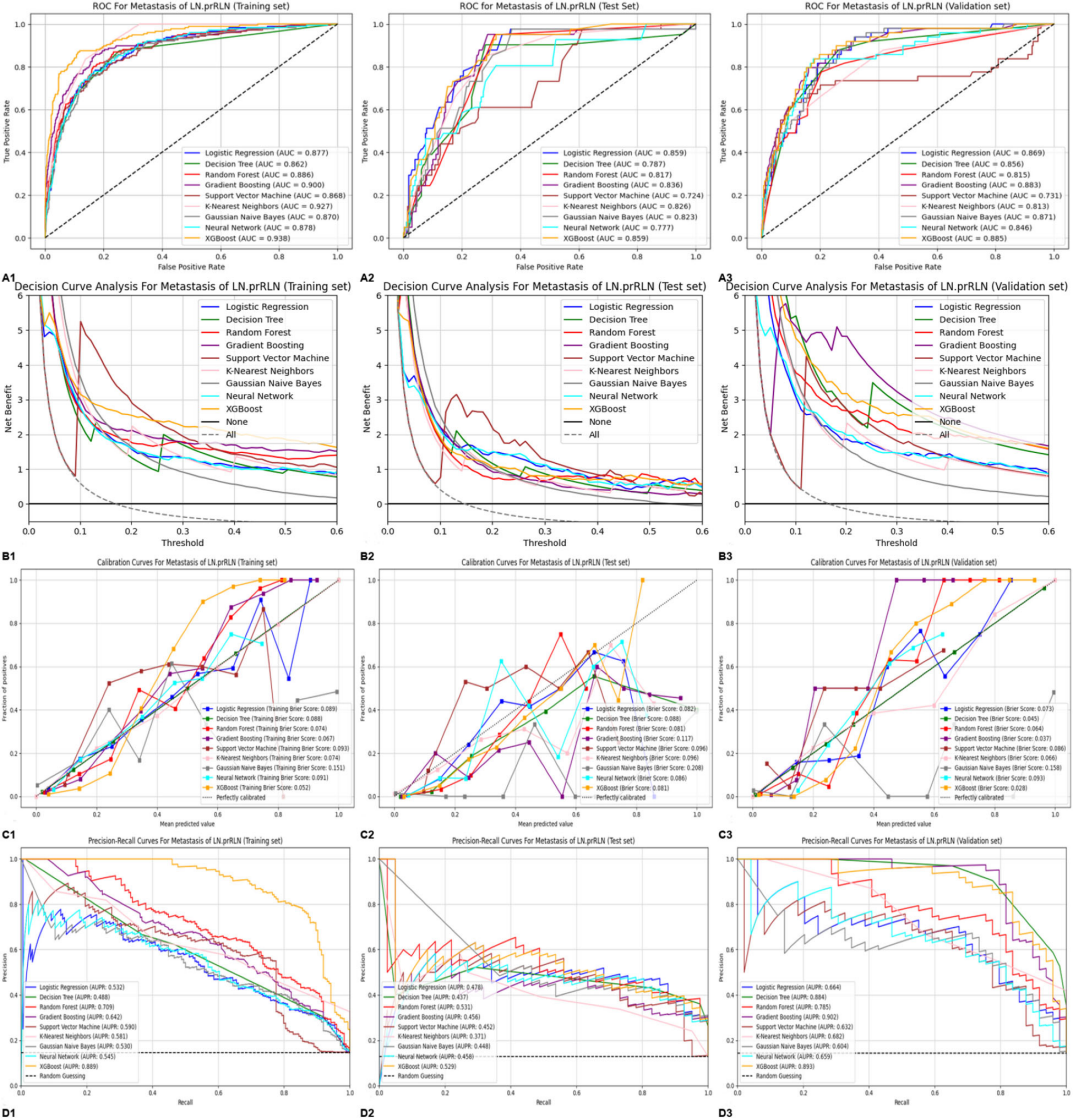
**(A)** ROC curves of the 9 machine learning predictive models on the training, testing and validation sets:(A1) ROC curve on the training set,(A2) ROC curve on the testing set,(A3) ROC curve on the validation set,**(B)** DCA curves of the 9 machine learning predictive models on the training, testing and validation sets:(B1) DCA curve on the training set,(B2) DCA curve on the testing set,(B3) DCA curve on the validation set; **(C)** Calibration curves of the 9 machine learning predictive models on the training, testing and validation sets:(C1) Calibration curve on the training set,(C2) Calibration curve on the testing set,(C3) Calibration curve on the validation set; **(D)** Precision-recall curves of the 9 machine learning predictive models on the training, testing and validation sets:(D1) Precision-recall curve on the training set,(D2) Precision-recall curve on the testing set,(D3) Precision-recall curve on the validation set. ROC, Receiver Operating Characteristic; DCA, Decision curve analysis; XGBoost, Extreme Gradient Boosting; LN-prRLN, Lymph Nodes posterior to the Recurrent Laryngeal Nerve.

All clinical, ultrasound, and intraoperative frozen pathology variables were used in nine ML algorithms. XGBoost was the optimal model for assessing LN-prRLN metastasis preoperatively and intraoperatively, with AUC values of 0.938, 0.885, and 0.859, respectively (**Figures 4A1-A3**). Decision curve analysis demonstrated superior net benefit of the XGBoost model across various threshold probabilities (**Figures 4B1-B3**).Calibration curves and Brier scores indicated low error rates (**Figures 4C1-C3**). Precision-recall curves showed high precision and accuracy for XGBoost (**Figures 4D1-D3**). Compared to the traditional model, XGBoost had higher AUC values and better clinical utility (**Table 3**; **Figures 3C, D**).

**Table 3:** Evaluation metrics for nine machine learning prediction models across training, testing and validation datasets.

Data sets	Model	Accuracy	AUC	Specificity	Sensitivity/recall	Negative predictive value	Positive predictive value/precision	F1 score	False positive rate
Training set	Logistic Regression	0.880	0.877	0.968	0.369	0.899	0.663	0.474	0.032
	Decision Tree	0.882	0.862	0.954	0.460	0.911	0.633	0.533	0.046
	Random Forest	0.886	0.886	0.978	0.352	0.898	0.729	0.475	0.022
	Gradient Boosting	0.898	0.900	0.980	0.420	0.908	0.787	0.548	0.020
	Support Vector Machine	0.876	0.868	0.966	0.352	0.897	0.639	0.454	0.034
	K-Nearest Neighbors	0.894	0.927	0.968	0.466	0.913	0.713	0.564	0.032
	Gaussian Naive Bayes	0.834	0.870	0.852	0.727	0.948	0.459	0.563	0.148
	Neural Network	0.880	0.878	0.961	0.409	0.904	0.643	0.500	0.039
	Extreme Gradient Boosting	0.910	0.938	0.983	0.483	0.917	0.833	0.612	0.017
Testing set	Logistic Regression	0.878	0.859	0.960	0.317	0.905	0.542	0.400	0.040
	Decision Tree	0.853	0.787	0.928	0.341	0.905	0.412	0.373	0.072
	Random Forest	0.871	0.817	1.000	0.000	0.871	0.000	0.000	0.000
	Gradient Boosting	0.834	0.836	0.910	0.317	0.900	0.342	0.329	0.090
	Support Vector Machine	0.871	0.724	1.000	0.000	0.871	0.000	0.000	0.000
	K-Nearest Neighbors	0.846	0.826	0.917	0.366	0.907	0.395	0.380	0.083
	Gaussian Naive Bayes	0.796	0.823	0.838	0.512	0.921	0.318	0.393	0.162
	Neural Network	0.878	0.777	0.978	0.195	0.892	0.571	0.291	0.022
	Extreme Gradient Boosting	0.862	0.859	0.935	0.366	0.909	0.455	0.405	0.065
Validation set	Logistic Regression	0.875	0.869	0.993	0.163	0.877	0.800	0.271	0.007
	Decision Tree	0.866	0.856	0.993	0.102	0.869	0.714	0.179	0.007
	Random Forest	0.857	0.815	1.000	0.000	0.857	0.000	0.000	0.000
	Gradient Boosting	0.883	0.883	0.983	0.286	0.892	0.737	0.412	0.017
	Support Vector Machine	0.880	0.731	0.986	0.245	0.887	0.750	0.369	0.014
	K-Nearest Neighbors	0.883	0.813	0.969	0.367	0.902	0.667	0.474	0.031
	Gaussian Naive Bayes	0.822	0.871	0.857	0.612	0.930	0.417	0.496	0.143
	Neural Network	0.872	0.846	0.976	0.245	0.886	0.632	0.353	0.024
	Extreme Gradient Boosting	0.875	0.885	0.963	0.347	0.898	0.607	0.442	0.037

### 3.4 Comparison of the optimal ML prediction model (XGBoost) and the traditional prediction model (nomogram prediction)

**Figure 5:**
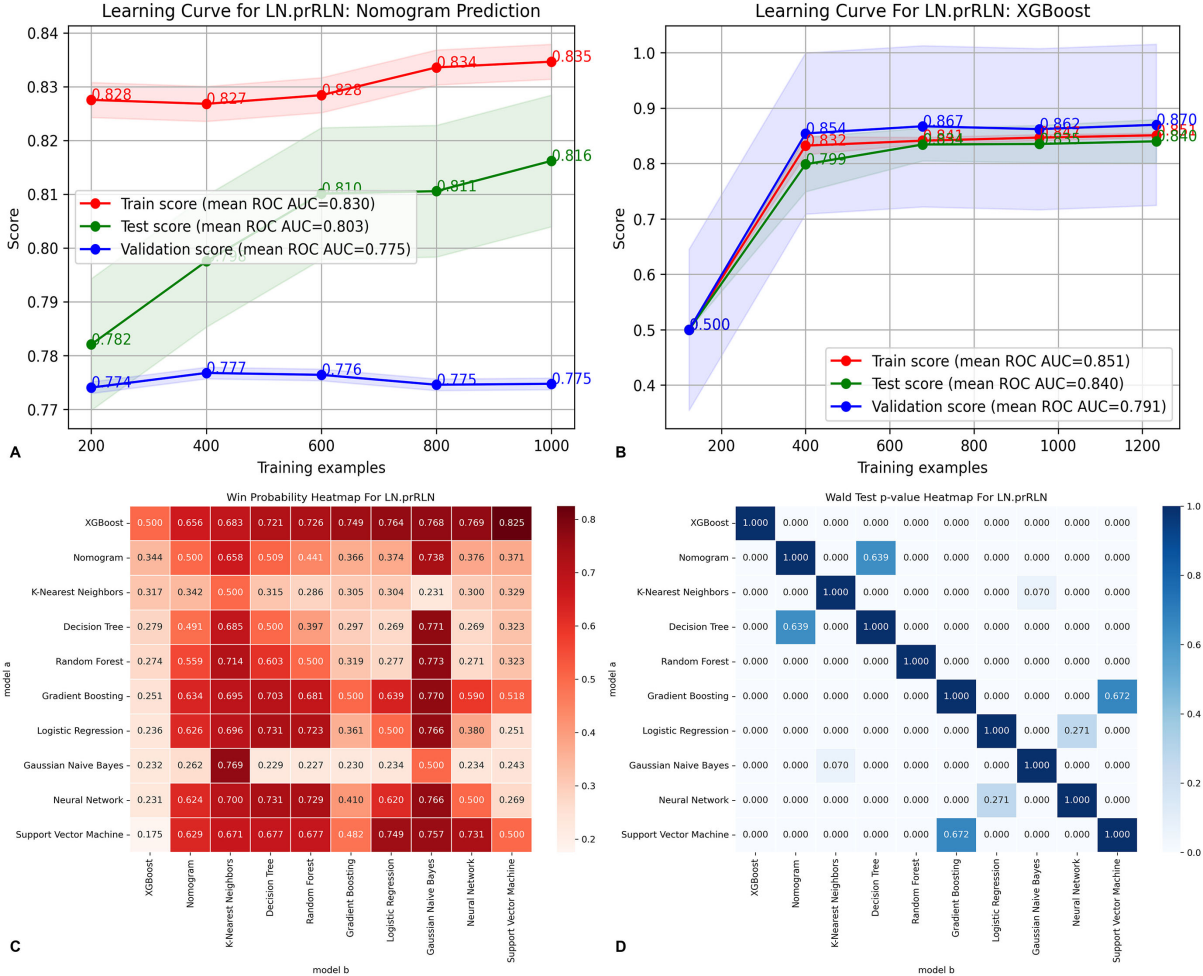
Comparison of learning curves and win probabilities estimated by PMRA and Wald test p-values for the traditional nomogram and machine learning models in predicting metastasis of LN-prRLN, **(A)** Learning curve of the traditional predictive model for Metastasis of LN-prRLN; **(B)** Learning curve of the XGBoost predictive model for Metastasis of LN-prRLN; **(C)** Win probabilities estimated by PMRA for predicting Metastasis of LN-prRLN; **(D)** Wald test p-values for predicting Metastasis of LN-prRLN. PMRA, Probability-based Ranking Model Approach; LN-prRLN, Lymph Nodes posterior to the Recurrent Laryngeal Nerve; XGBoost, Extreme Gradient Boosting.

ROC curve analysis revealed that the XGBoost model had higher AUC values across all datasets: 0.938 (training set), 0.859 (testing set), and 0.885 (validation set), compared to the nomogram’s 0.865, 0.814, and 0.836, respectively (**Figures 3A, C**). The DCA curves further confirmed the superior clinical utility of the XGBoost model, as its curve consistently outperformed the nomogram’s curve (**Figures 3B, D**). The learning curve also showed higher mean AUC values for the XGBoost model, especially in the training and testing sets (**Figures 5A, B**). Based on the PMRA for predicting LN-prRLN metastasis, the XGBoost model had the highest probability of winning. Another eight models showed significant differences from the XGBoost model, though their probabilities of winning were all below 0.5 (**Table 4** and **Figures 5C, D**).

**Table 4:** Ranking by probability-based ranking model approach (PMRA) for predicting ipsilateral lateral lymph node metastasis.

	Models	Probability of Winning Against the Top Model (PMRA)	Wald p-value
1	XGBoost	/	/
2	Nomogram	0.344	<0.001
3	K-Nearest Neighbors	0.317	<0.001
4	Decision Tree	0.279	<0.001
5	Random Forest	0.274	<0.001
6	Gradient Boosting	0.251	<0.001
7	Logistic Regression	0.236	<0.001
8	Gaussian Naive Bayes	0.232	<0.001
9	Neural Network	0.231	<0.001
10	Support Vector Machine	0.175	<0.001

### 3.5 Visualization and application of XGBoost model using SHAP

**Figure 6:**
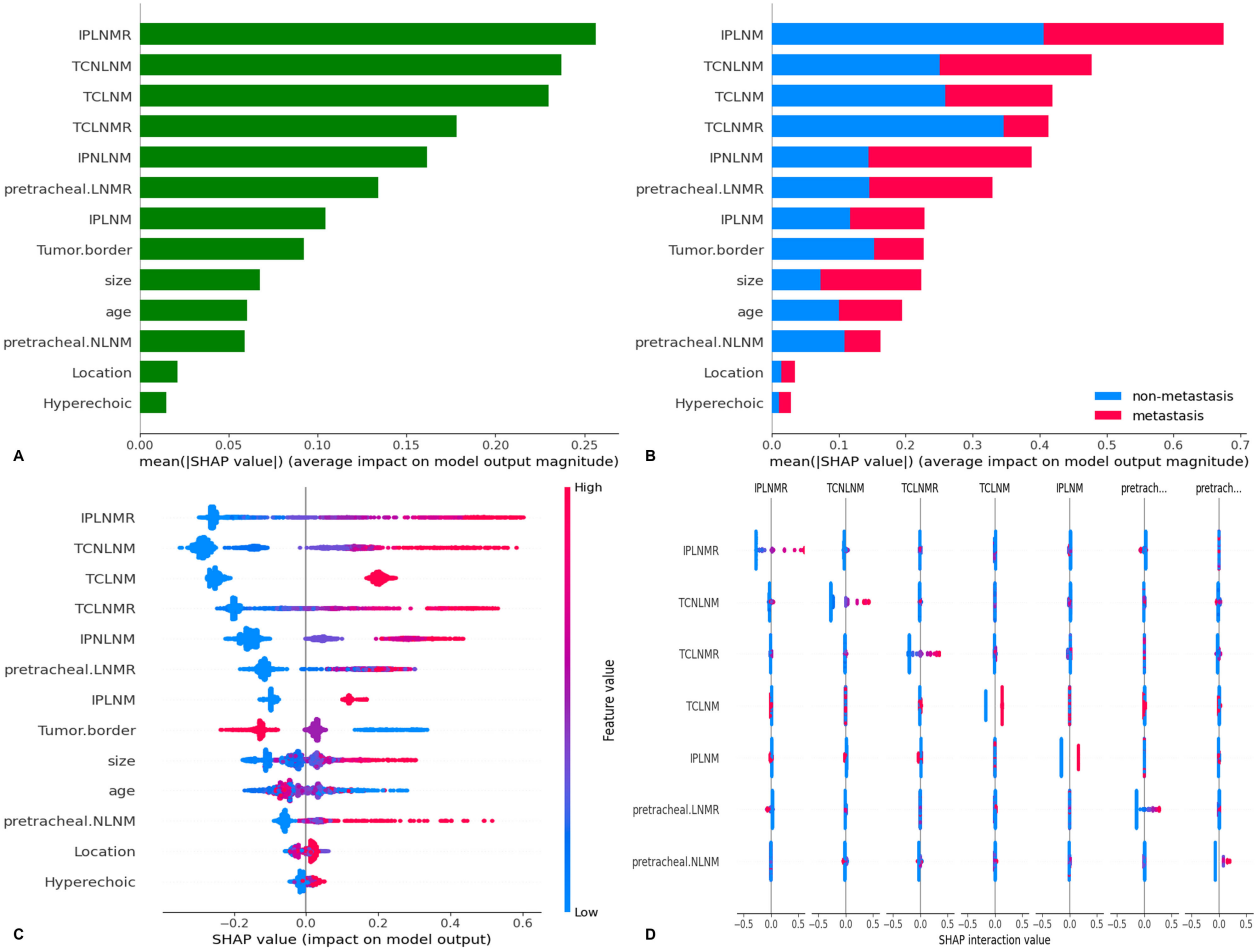
SHAP analysis of the XGBoost model: **(A)** Standard bar chart of SHAP summary plot, showing the impact of each feature on the XGBoost model; **(B)** Categorical bar chart of SHAP summary plot, also showing the impact of each feature; **(C)** SHAP summary scatter plot, visually reflecting the relationship between feature values and predicted probabilities; **(D)** SHAP interaction plot for the top 7 features affecting the prediction of Metastasis of LN-prRLN.SHAP, Shapley Additive Explanations; XGBoost, Extreme Gradient Boosting; LN-prRLN, Lymph nodes posterior to the recurrent Laryngeal Nerve; LNM, Lymph Node Metastasis; LNMR, Lymph Node Metastasis Ratio; IPLNM, Ipsilateral paratracheal lymph node metastasis; IPLNMR, Ratio of Ipsilateral Paratracheal Lymph Node Metastasis; IPNLNM, Number of Ipsilateral Paratracheal Lymph Node Metastasis; TCLNM, Total Central Lymph Node Metastasis; TCLNMR, Ratio of Total Central Lymph Node Metastasis; TCNLNM, Number of Total Central Lymph Node Metastasis.

**Figure 7:**
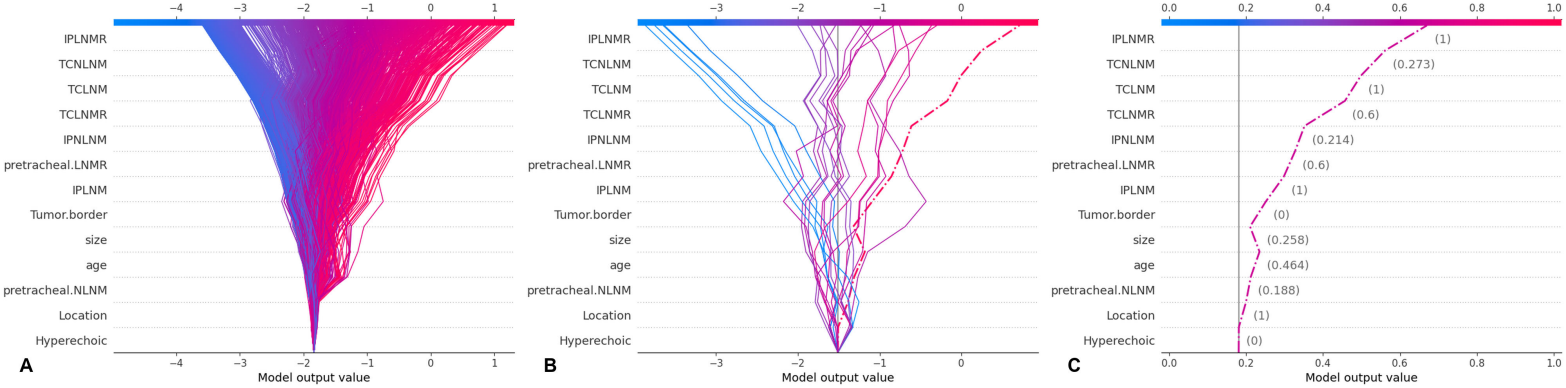
**(A)** SHAP decision plot for all PTC patients; **(B)** SHAP decision plot for 20 randomly selected PTC patients, with one misclassified case (shown by the dashed line); **(C)** Detailed SHAP decision plot for the misclassified case. SHAP, Shapley Additive Explanations; PTC, Papillary Thyroid Carcinoma; XGBoost, Extreme Gradient Boosting; LN-prRLN, Lymph nodes posterior to the recurrent laryngeal nerve; LNM, Lymph Node Metastasis; LNMR, Lymph Node Metastasis Ratio; IPLNM, Ipsilateral paratracheal lymph node metastasis; IPLNMR, Ratio of Ipsilateral Paratracheal Lymph Node Metastasis; IPNLNM, Number of Ipsilateral Paratracheal Lymph Node Metastasis; TCLNM, Total Central Lymph Node Metastasis; TCLNMR, Ratio of Total Central Lymph Node Metastasis; TCNLNM, Number of Total Central Lymph Node Metastasis.

SHAP values for all patients in the training set were visualized in bar charts (**Figure 6A**). The classification bar chart showed the distribution of LN-prRLN metastasis cases (red) and non-metastasis cases (blue) (**Figure 6B**). The top ten parameters were IPLNMR, TCNLNM, TCLNM, TCLNMR, IPNLNM, pretracheal LNM ratio, IPLNM, tumor border, size, and age. SHAP summary plots reflected the relationship between parameters and predicted probabilities (**Figure 6C**). Higher SHAP values for certain LNM indicated higher LN-prRLN metastasis likelihood; conversely, higher values for age > 39 years and tumor diameter < 10 mm indicated lower likelihood. The tumor border had a positive effect, where a clear border indicated a lower likelihood of metastasis. SHAP interaction and decision plots demonstrated parameter effects on predictions (**Figures 6D**, **7**).

### 3.6 Creating a web calculator for feature contribution visualization using SHAP

**Figure 8:**
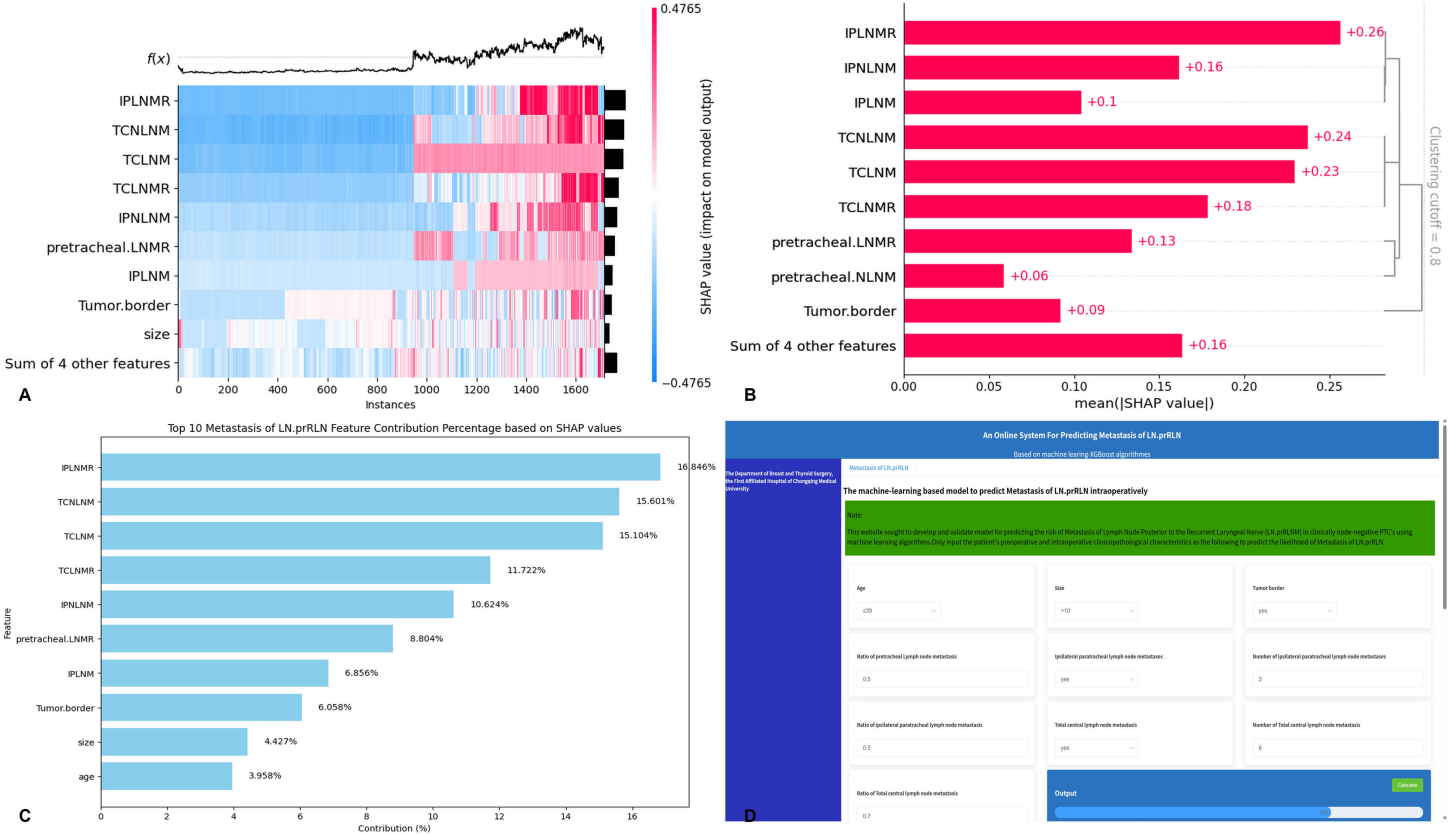
**(A)** SHAP absolute value heatmap for all PTC patients; **(B)** Bar chart of SHAP mean absolute values with clustering analysis for PTC patients; **(C)** Bar chart showing the contribution percentage of the top 10 feature variables based on SHAP values; **(D)** Web-based calculator interface, visualizing the ML model and SHAP interpretations. SHAP, Shapley Additive Explanations; PTC, Papillary Thyroid Carcinoma; ML, Machine Learning; XGBoost, Extreme Gradient Boosting; LN-prRLN, Lymph nodes posterior to the Recurrent Laryngeal Nerve; LNM, Lymph Node Metastasis; LNMR, Lymph Node Metastasis Ratio; IPLNM, Ipsilateral paratracheal lymph node metastasis; IPLNMR, Ratio of Ipsilateral Paratracheal Lymph Node Metastasis; IPNLNM, Number of Ipsilateral Paratracheal Lymph Node Metastasis; TCLNM, Total Central Lymph Node Metastasis; TCLNMR, Ratio of Total Central Lymph Node Metastasis; TCNLNM, Number of Total Central Lymph Node Metastasis.

The contribution calculation heatmap and bar chart visually presented each feature’s contribution to the model output (**Figures 8A, B**). The top ten ranked feature variables were IPLNMR (SHAP value of 0.26, contribution ratio of 16.846%), TCNLNM (SHAP value of 0.24, 15.601%), TCLNM (SHAP value of 0.23, 15.104%), TCLNMR (SHAP value of 0.18, 11.722%), IPNLNM (SHAP value of 0.16, 10.624%), pretracheal LNM ratio (SHAP value of 0.13, 8.804%), IPLNM (SHAP value of 0.1, 6.856%), unclear tumor border (SHAP value of 0.09, 6.058%), size (SHAP value of 0.07, 4.427%), and age ≤39 years (SHAP value of 0.06, 3.958%), with a gradual decrease in the positive contribution to LN-prRLN metastasis (**Figure 8C**). The top ten feature variables were selected to create a web calculator, with contribution ratios shown (**Figure 8D**). The web calculator can be accessed at:(http://121.41.36.155:9003/static/html/index.html).

## 4 Discussion

The rate of metastasis in LN-prRLN varies between 6% and 34.1% in literature (3, 4, 8, 27), with cN0 PTC patients showing rates up to 22.4% (14, 25, 37). There remains controversy regarding the prophylactic dissection of LN-prRLN (37, 38), focusing on balancing surgical benefits and avoiding complications. Preoperative prediction of LN-prRLN metastasis in cN0 PTC is critical for precise management. Accurate lymph node assessment is essential for selective lymph node dissection (12, 39). Therefore, a reliable and rapid lymph node evaluation tool is necessary. In this multicenter retrospective study, we developed an integrated model combining clinical, ultrasound, and intraoperative frozen pathology assessments to predict LN-prRLN metastasis in cN0 PTC patients. This model helps determine the necessity and timing of LN-prRLN dissection.

Several studies have explored factors influencing LN-prRLN metastasis, mostly focusing on cN1 patients (3, 14, 21, 39). For instance, Zou M et al. identified age < 45 years (p = 0.005; OR 2.155), male gender (p = 0.043; OR 1.657), tumor diameter > 1.0 cm (p = 0.042; OR 1.702), microcalcifications (p = 0.022; OR 1.980), and lateral LNM on ultrasound (p = 0.001; OR 2.578) as independent risk factors for LN-prRLN metastasis (17);Bae SY et al. found tumor size>1.0 cm (p= 0.009, OR = 2.654) and CLNM (p< 0.001, OR = 5.005) significantly associated with LN-prRLN metastasis (18). Chang H et al. suggested that CLNM (excluding LN-prRLN metastasis, p<0.001,OR=14.715) and LLNM (p=0.016,OR=4.383) significantly predict LN-prRLN metastasis (13). In these studies (14, 23, 26), cN1 patients were analyzed alongside cN0 patients, although cN0 patients also have a high rate of occult LNM. Our study found a 14.177% rate of LN-prRLN metastasis in cN0 PTC, similar to previous studies (4, 25). Prophylactic LN-prRLN dissection requires more accurate predictive models and characteristic variables for precision.

Tumor size is a crucial factor influencing LN-prRLN metastasis, with a cutoff value typically around 1.0 cm. Qi GF et al. showed that a maximum tumor diameter > 1.0 cm (p < 0.001,OR: 5.729) is an independent risk factor for LN-prRLN metastasis (27). Xiao X et al. found a tumor diameter > 1.0 cm (p<0.001, OR = 2.897) to be an independent risk factor (37). Our study identified a tumor size cutoff of 10 mm for LN-prRLN metastasis, consistent with other studies (8, 9, 18). In SHAP analysis, size > 10 mm ranked ninth, contributing 4.427%. Indistinct tumor boundaries were the only significant ultrasound feature (SHAP value 0.09), consistent with another research (27). Lower tumor location and microcalcifications also significantly influence LN-prRLN metastasis, albeit with smaller contributions, aligning with previous studies (8, 11). Age is a recognized factor affecting LNM (8, 22), with our ROC analysis setting the optimal cutoff at 39 years, possibly due to early-stage detection through routine examinations.

Most published studies analyze postoperative pathology or clinical data, lacking methods to guide intraoperative decisions (39, 40). Early-stage patients need suitable indicators for preoperative and intraoperative LN-prRLN status assessment to avoid unnecessary lymph node dissection and nerve injury (41, 42). Intraoperative frozen section provides immediate pathological results, aiding surgical decisions and avoiding unnecessary reoperations (43–45). Intraoperative frozen section accuracy for thyroid cancer CLNM is typically 80%-90% (46, 47). Our study’s intraoperative frozen pathology identified variables contributing 85.557% to LN-prRLN metastasis prediction. The most significant factor was IPLNM, which is the primary independent factor in traditional models (P<0.001, OR = 3.605). ML-based SHAP analysis of the optimal model revealed that the SHAP value for the proportion of IPLNM was highest at 0.26, constituting 16.846% (**Figure 8C**). Clustering analysis through SHAP visualization identified IPLNM status (presence, number, and proportion) as the primary category influencing LN-prRLN metastasis. Consistent with previous studies, this research provides more accurate quantification of feature variables. For instance, studies by Shen B et al. identified paratracheal LNM as an independent risk factor for LN-prRLN (p<0.001, OR = 5.357) (1), while Yang H et al. highlighted LN-arRLN as an independent risk factor (p<0. 001, OR = 4. 386) (11). Similar findings were reported in studies (14, 37, 48). The second most influential factor on LN-prRLN metastasis in this study is TCLNM, excluding LN-prRLN metastasis, which ranks second in traditional prediction models (P = 0.013, OR = 2.633) (**Figures 2A, B**). SHAP analysis clustering identified TCNLNM with the highest SHAP value of 0.24, accounting for 15.601%. Consistent with prior research, studies by Bae SY found significant correlations between TCLNM (P = 0.001, OR = 5.005) and LN-prRLN metastasis (18). Kim D et al. ‘s study showed TCLNM (p<0.001,OR=5.203) was LN-prRLN metastasis (10). Furthermore, Ling Y et al. interpreted the role of intraoperative frozen sections, highlighting the number of metastatic lymph nodes during LN-prRLN as a potential predictive factor (P = 0.014, OR = 1.320). The false negative rate was quite low (6.2%), suggesting that cN0 patients may not require LN-prRLN resection when lymph node biopsy does not show metastasis. Number of prelaryngeal, pretracheal, and paratracheal LNM are risk factors for LN-prRLN metastasis (25). Our previous study also confirmed that number of prelaryngeal, pretracheal, and paratracheal LNM (p<0.001, OR = 21.078) were independent predictors of LN-prRLN metastasis (19). This study further elucidates paratracheal LNM as the third category of risk factors influencing LN-prRLN metastasis, possibly related to thyroid lymphatic drainage anatomically through the lower thyroid veins, distributing lymph from the thyroid to the paratracheal area, then to the lateral neck nodes, and finally returning to the lymphatic duct or thoracic duct, anatomically referred to as the descending pathway (7, 15).

Of greater significance in this study is the comparison between traditional prediction models and artificial intelligence ML features. Traditional prediction models play an important role in predicting LNM to some extent, but they have limitations. In this study, the predictive value of traditional prediction models was relatively high, with a training set AUC of 0.865 (**Figure 3A**), similar to previous reports (14, 18, 21). However, XGBoost selected through ML showed higher predictive value with a training set AUC of 0.938, and good performance in the testing and validation sets with AUCs of 0.859 and 0.885, respectively. DCA curves, calibration curves, and precision-recall curves demonstrated the model’s clinical applicability, high accuracy, and low error rate. The XGBoost model enables precise prediction of LN-prRLN metastasis in cN0 PTC patients and successfully identifies key parameters influencing LN-prRLN metastasis prediction. SHAP provides reasonable visual explanations for predictions, encompassing both positive and negative impacts. In this study, we not only demonstrated the overall feature importance for predicting LN-prRLN metastasis but also developed a network calculator using SHAP visualizations of contribution degrees. This tool offers practical utility for clinicians to predict LN-prRLN metastasis based on preoperative and intraoperative feature variables, guiding surgical approaches and precise surgical planning.

Limitations of this study warrant discussion. Firstly, to mitigate the issue of a relatively small sample size, we subjected the constructed XGBoost model to ten-fold cross-validation to demonstrate sufficient accuracy. Secondly, this study employed a retrospective design. However, to enhance model credibility, we conducted internal and external validations, achieving high AUC values. Additionally, we compared XGBoost evaluation results with those of traditional ML model, further proving its superior performance. Thirdly, during the construction of the XGBoost model and the creation of the network calculator, manual input of relevant parameters was required. When clinicians record image features, some unknown or overlooked aspects may lead to the loss of hidden relationships. To minimize this phenomenon, we conducted consistent data analysis to ensure model accuracy. Fourthly, our model was developed using single-center data, which may introduce potential biases related to institutional practices and patient demographics. Although external validation was performed using 319 patients from another hospital in the same city, this validation remains geographically limited and may not fully represent broader clinical populations across different regions. In conclusion, SHAP provides reasonable visual explanations for the XGBoost model, applicable independently for predicting LN-prRLN metastasis in cN0 PTC patients, including both positive and negative impacts, significantly enhancing clinician confidence in the clinical application of the XGBoost model. Future studies are expected to validate the XGBoost model through prospective studies with broad samples from multiple centers, widely applying it in clinical practice to guide personalized treatment of LN clearance in PTC patients.

## 5 Conclusion

We developed and internally and externally validated nine ML prediction models using preoperative and intraoperative frozen pathological features. These models were compared with traditional prediction models, and the XGBoost algorithm emerged as the optimal predictive model. Through SHAP visualization applications, we identified the top ten feature variables influencing LN-prRLN metastasis in cN0 PTC patients and calculated their contributions using SHAP analysis. Cluster analysis demonstrated the importance of intraoperative frozen pathological examination, which can be utilized for personalized prediction of LN-prRLN metastasis in cN0-PTC patients. ML-based prediction models accurately identify whether patients are at risk of LN-prRLN metastasis. The online network calculator created based on SHAP analysis of contribution percentages serves as an easy-to-use tool for clinicians to make precise surgical decisions. In the future, we aim to integrate imaging and molecular data to further optimize the model’s performance in the field of personalized precision medicine. Additionally, more multicenter studies are needed to further validate our findings.

## Data Availability

The datasets presented in this study can be found in online repositories. The names of the repository/repositories and accession number(s) can be found in the article/**Supplementary Material**.
