# Supplementary material for: Machine learning and Shapley Additive exPlanations to predict metastasis of lymph nodes posterior to the recurrent laryngeal nerve in cN0 papillary thyroid carcinoma
Source: Front Oncol. 2026 Jan 7;15:1673332. doi: 10.3389/fonc.2025.1673332 (PMC12819197; doi:10.3389/fonc.2025.1673332)
Supplement: Supplementary file 1 [file Table1.docx]

Supplementary Material 1

| **Table 1: Overall Data and Feature Balance across Training, Validation, and Test Sets** | | | | | |
| --- | --- | --- | --- | --- | --- |
| **Characteristics** | **Total (N=1714)** | **Training set (N=1200)** | **Validation set (N=514)** | **Test set (N=319)** | **Univariate** |
|  |  |  |  |  | **p-value** |
| **Age** | **42.6± 11.696** | 42.493 ± 11.803 | 42.848 ± 11.450 | 41.301 ± 11.275 | 0.158 |
| age>39 | 993(57.935%) | 685（57.083%） | (308, 59.922%) | (156, 48.903%) | 0.987 |
| age≤39 | 721(42.065%) | 515（42.917%) | (206, 40.078%) | (163, 51.097%) |  |
| **Sex** |  |  |  |  |  |
| Female | 1250(72.929%) | 886（73.833%) | (364, 70.817%) | (280, 87.774%) | 0.954 |
| Male | 464(27.071%) | 314（26.167%) | (150, 29.183%) | (39, 12.226%) |  |
| **BMI** | **23.257 ± 3.250** | 23.302 ± 3.256 | 23.152 ± 3.235 | 23.613 ± 3.399 | 0.14 |
| Normal | 1006(58.693%) | 701（58.417%) | (305, 59.339%) | (173, 54.232%) | 1 |
| Overweight | 630(36.756%) | 448, 37.333%) | (182, 35.409%) | (130, 40.752%) |  |
| Underweight | 78(4.551%) | (51, 4.250%) | (27, 5.253%) | (16, 5.016%) |  |
| **Tumor.border** |  |  |  |  |  |
| extrandular-invasion | 687(40.082%) | (139, 11.583%) | (55, 10.700%) | (17, 5.329%) | 0.987 |
| irregular-shape/lsharpobed | 833(48.6%) | (582, 48.500%) | (251, 48.833%) | (267, 83.699%) |  |
| smooth/borderless | 194(11.319%) | (479, 39.917%) | (208, 40.467%) | (35, 10.972%) |  |
| **Aspect.ratio** |  |  |  |  |  |
| ≤1 | 850(49.592%) | (615, 51.250%) | (249, 48.444%) | (107, 33.542%) | 0.964 |
| >1 | 864(50.408%) | (585, 48.750%) | (265, 51.556%) | (212, 66.458%) |  |
| **Ingredients** |  |  |  |  |  |
| cystic/cavernous | 8(0.467%) | (7, 0.583%) | (1, 0.195%) | (1, 0.313%) | 1 |
| Mixed cystic and solid | 40(2.334%) | (28, 2.333%) | (12, 2.335%) | (3, 0.940%) |  |
| solid | 1666(97.2%) | (1165, 97.083%) | (501, 97.471%) | (315, 98.746%) |  |
| **Internal.echo.pattern** |  |  |  |  |  |
| echoless | 3(0.175%) | (1, 0.083%) | 2, 0.389%) | (1, 0.313%) | 1 |
| high/isoechoic | 187(10.91%) | (130, 10.833%) | (57, 11.089%) | (28, 8.777%) |  |
| hypoechoic | 1467(85.589%) | (1026, 85.500%) | (441, 85.798%) | (286, 89.655%) |  |
| very hypoechoic | 57(3.326%) | (43, 3.583%) | (14, 2.724%) | (4, 1.254%) |  |
| **Internal.echo.homogeneous** |  |  |  |  |  |
| Uniform | 1032(60.21%) | (718, 59.833%) | (314, 61.089%) | (212, 66.458%) | 0.88 |
| Non-uniform | 682(39.79%) | (482, 40.167%) | (200, 38.911%) | (107, 33.542%) |  |
| **Hyperechoic** |  |  |  |  |  |
| no/large comet tail | 430(25.088%) | (300, 25.000%) | (130, 25.292%) | (71, 22.257%) | 0.813 |
| coarse calcification | 329(19.195%) | (239, 19.917%) | (90, 17.510%) | (21, 6.583%) |  |
| peripheral calcification | 51(2.975%) | (38, 3.167%) | (13, 2.529%) | (6, 1.881%) |  |
| Microcalcification | 904(52.742%) | (623, 51.917%) | (281, 54.669%) | (221, 69.279%) |  |
| **Tumor.Peripheral.blood.flow** |  |  |  |  |  |
| Without | 1395(81.389%) | (971, 80.917%) | (97, 18.872%) | (142, 44.514%) | 0.995 |
| Abundant | 319(18.611%) | (229, 19.083%) | (417, 81.128%) | (177, 55.486%) |  |
| **Tumor.internal.vascularization** |  |  |  |  |  |
| Without | 1388(80.98%) | (977, 81.417%) | (418, 81.323%) | (171, 53.605%) | 0.998 |
| Abundant | 326(19.02%) | (223, 18.583%) | (96, 18.677%) | (148, 46.395%) |  |
| **Size** | **11.740 ± 8.183** | 11.936 ± 8.497 | 11.284 ± 7.383 | 10.343 ± 7.162 | 0.51 |
| >10 | 672(39.207%) | (482, 40.167%) | (190, 36.965%) | (106, 33.229%) | 1 |
| ≤10 | 1042(60.793%) | (718, 59.833%) | (324, 63.035%) | (213, 66.771%) |  |
| **Location** |  |  |  |  |  |
| Upper | 321(18.728%) | 222, 18.500% | 99（19.261%) | 39（12.226%) | 0.987 |
| Middle | 665(38.798%) | (459, 38.250%) | 206（40.078%) | 111（34.796%) |  |
| Under | 482(28.121%) | 343, 28.583% | 139（27.043%) | 78（ 24.451%) |  |
| Multisite | 222(12.952%) | 159, 13.250% | 63（12.257%) | 84（ 26.332%) |  |
| Isthmus | 24(1.4%) | 17（1.417%) | 7（1.362%) | 7（2.194%) |  |
| **ETE** |  |  |  |  |  |
| Without | 1493(87.106%) | (1043, 86.917%) | (450, 87.549%) | (273, 85.580%) | 1 |
| Abundant | 221(12.894%) | (157, 13.083%) | (64, 12.451%) | (46, 14.420%) |  |
| **Mulifocality** |  |  |  |  |  |
| Without | 1297(75.671%) | 900（75.000%) | 397,（77.237%) | 201（ 63.009%) | 1 |
| Abundant | 417(24.329%) | 300（25.000%） | 117（22.763%) | 118（36.991%) |  |
| **Hashimoto** |  |  |  |  |  |
| Without | 1365(79.638%) | 959（79.917%) | 406,（78.988%) | 227（71.160%) | 1 |
| Abundant | 349(20.362%) | 241（20.083%) | 108（21.012%) | 92（28.840%) |  |
| **T.staging** |  |  |  |  |  |
| 1 | 1349(78.705%) | 941(78.417%) | 408(79.377%) | 261( 81.818%) | 1 |
| 2 | 135(7.876%) | 94(7.833%) | 41(7.977%) | 13(4.075%) |  |
| 3 | 177(10.327%) | 126( 10.500%) | 51( 9.922%) | 35(10.972%) |  |
| 4 | 53(3.092%) | 39(3.250%) | 14(2.724%) | 10(3.135%) |  |
| **prelaryngeal.LNM** |  |  |  |  |  |
| No | 1302(84.326%) | 914(76.167%) | 388(75.486%) | 233( 73.041%) | 0.997 |
| Yes | 242(15.674%) | 175(14.583%) | 67(13.035%) | 44( 13.793%) |  |
|  |  | 111(9.250%) | 59(11.479%) | 42( 13.166%) |  |
| **prelaryngeal.LNMR** |  |  |  |  |  |
| Mean±SD | 0.111 ± 0.283 | 0.116 ± 0.289 | 0.100 ± 0.266 | 0.084 ± 0.229 |  |
| **prelaryngeal.NLNM** |  |  |  |  |  |
| Mean±SD | 0.238 ± 0.657 | 0.244 ± 0.659 | 0.222 ± 0.651 | 0.209 ± 0.524 |  |
| **pretracheal.LNM** |  |  |  |  |  |
| No | 1153(67.704%) | 807(67.250%) | 346(67.315%) | 210( 65.831%) | 0.997 |
| Yes | 550(32.296%) | 388(32.333%) | 162(31.518%) | 109(34.169%) |  |
|  |  | 5(0.417%) | 6(1.167%) | 0(0%) |  |
| **pretracheal.LNMR** |  |  |  |  |  |
| Mean±SD | 0.176 ± 0.308 | 0.179 ± 0.312 | 0.170 ± 0.298 | 0.194 ± 0.317 | 0.189 |
| **pretracheal.NLNM** |  |  |  |  |  |
| Mean±SD | 0.665± 1.327 | 0.676 ± 1.339 | 0.640 ± 1.300 | 0.690 ± 1.158 | 0.653 |
| **IPLNM** |  |  |  |  |  |
| No | 1160(67.678%) | 810( 67.500%) | 350( 68.093%) | 203(63.636%) | 0.998 |
| Yes | 554(32.322%) | 390(32.500%) | 164(31.907%) | 116(36.364%) |  |
| **IPLNMR** |  |  |  |  |  |
| Mean±SD | 0.200 ± 0.337 | 0.199 ± 0.336 | 0.201 ± 0.342 | 0.147 ± 0.224 | 0.553 |
| **IPNLNM** |  |  |  |  |  |
| Mean±SD | 0.653 ± 1.265 | 0.646 ± 1.206 | 0.669 ± 1.393 | 0.683 ± 1.191 | 0.83 |
| **TCLNM** |  |  |  |  |  |
| No | 946(55.193%) | 664(55.333%) | 282(54.864%) | 160(50.157%) | 0.498 |
| Yes | 768(44.807%) | 536(44.667%) | 232(45.136%) | 159( 49.843%) |  |
| **TCLNMR** |  |  |  |  |  |
| Mean±SD | 0.172 ± 0.254 | 0.174 ± 0.255 | 0.167 ± 0.253 | 0.156 ± 0.203 | 0.46 |
| **TCNLNM** |  |  |  |  |  |
| Mean±SD | 1.528± 2.500 | 1.541 ± 2.456 | 1.498 ± 2.596 | 1.555 ± 2.167 | 0.867 |
