# Supplementary material for: Machine learning and Shapley Additive exPlanations to predict metastasis of lymph nodes posterior to the recurrent laryngeal nerve in cN0 papillary thyroid carcinoma
Source: Front Oncol. 2026 Jan 7;15:1673332. doi: 10.3389/fonc.2025.1673332 (PMC12819197; doi:10.3389/fonc.2025.1673332)
Supplement: Supplementary file 2 [file Table2.docx]

Supplementary Material 2

##

| Table1 The method and number of screening variables | | | |
| --- | --- | --- | --- |
| Feature variable filtering method | Max AUC | Number of variables | Characteristic variables |
|  |  |  |  |
| Removing variance characteristics | 0.907 | 11 | Age','Sex','Aspect.ratio','Internal.echo.homogeneous','Size','location','Mulifocality','Hashimoto','pretracheal.LNM','IPLNM','TCLNM' |
| Univariate Feature Selection | 0.917 | 10 | prelaryngeal.LNM','pretracheal.LNM','IPLNM','TCLNM','T.staging','prelaryngeal.LNMR','pretracheal.LNMR','IPLNMR','TCLNMR','TCNLNM' |
| Recursive Feature Elimination (RFE) | 0.908 | 10 | prelaryngeal.LNM','pretracheal.LNM','IPLNM','TCLNM','prelaryngeal.NLNM','pretracheal.NLNM','IPLNMR','IPNLNM','TCLNMR','TCNLNM' |
| Recursive Feature Elimination with Cross-Validation (RFE-CV) | 0.919 | 10 | ETE','prelaryngeal.LNM','pretracheal.LNM','IPLNM','bmi','T.staging','prelaryngeal.LNMR','prelaryngeal.NLNM','pretracheal.LNMR','pretracheal.NLNM' |
| L1-based Feature Selection | 0.925 | 13 | BMI','Tumor.border','ETE','prelaryngeal.LNM','TCLNM','size','T.stage','prelaryngeal.NLNM','pretracheal.NLNM','IPLNMR','IPNLNM','TCLNMR','TCNLNM' |
| Tree-based Feature Selection | 0.938 | 12 | Tumor.border','Hyperechoic','Location','IPLNM','age','size','pretracheal.LNMR','pretracheal.NLNM','IPLNMR','IPNLNM','TCLNMR','TCNLNM' |

| Table2 ：The web calculator enters the feature variable assignment | | |
| --- | --- | --- |
| variables | SHAP contribution | Web calculator proportion |
| age | 3.957927 | 0.04 |
| size | 4.426855 | 0.04 |
| Tumor.border | 6.058465 | 0.06 |
| pretracheal.LNMR | 8.804328 | 0.09 |
| IPLNM | 6.855916 | 0.07 |
| IPNLNM | 10.623804 | 0.11 |
| IPLNMR | 16.845905 | 0.17 |
| TCLNM | 15.103771 | 0.15 |
| TCNLNM | 15.601066 | 0.15 |
| TCLNMR | 11.721963 | 0.12 |

## Variable name of lymph node behind recurrent laryngeal nerve

1. Total variable name：

Age,age,Sex,BMI,bmi,Tumor.border,Aspect.ratio,**Ingredients,**Internal.echo.pattern,Internal.echo.homogeneous,Hyperechoic,Tumor.internal.vascularization,Tumor.Peripheral.blood.flow,Size,size,location,Location,**Mulifocality,**Hashimoto,ETE,T.stage,prelaryngeal.LNM,prelaryngeal.LNMR,prelaryngeal.NLN,prelaryngeal.NLNM,pretracheal.LNM,**pretracheal.LNMR,**pretracheal.NLN,pretracheal.NLNM,IPLNM,IPLNMR,IPNLNM,TCLNM,TCLNMR,TCNLNM,LN.prRLNM

- 1. Categorical variables

"Sex","Tumor.border","Aspect.ratio","Ingredients","Internal.echo.pattern","Internal.echo.homogeneous","Hyperechoic","Tumor.internal.vascularization","Tumor.Peripheral.blood.flow","location","Location","Mulifocality","Hashimoto","ETE","T.stage","prelaryngeal.LNM","pretracheal.LNM","IPLNM","TCLNM","LN.prRLNM"

- 1. Numerical Variables

"Age","BMI","Size","prelaryngeal.LNMR","prelaryngeal.NLN","prelaryngeal.NLNM","pretracheal.LNMR","pretracheal.NLN","pretracheal.NLNM","IPLNMR","IPNLNM","TCLNMR","TCNLNM"

2.R language input variable name

2.1 Single factor analysis of meaningful variables

Age,Sex,Tumor.border,Tumor.internal.vascularization,Tumor.Peripheral.blood.flow,Size,size,**Mulifocality,**ETE,T.stage,prelaryngeal.LNM,prelaryngeal.LNMR,prelaryngeal.NLNM,pretracheal.LNM,**pretracheal.LNMR,**pretracheal.NLNM,IPLNM,IPLNMR,IPNLNM,TCLNM,TCLNMR,TCNLNM,

2.2Variables included in multi-factor analysis

Age+Sex+Tumor.border+Tumor.internal.vascularization+Tumor.Peripheral.blood.flow+Size+Mulifocality+ETE+T.staging+prelaryngeal.LNM+pretracheal.LNM+IPLNM+TCLNM

2.3 Variables included in the nomogram

Tumor.border+T.stage+prelaryngeal.LNM+pretracheal.LNM+IPLNM+TCLNM

3.Machine learning variable names

3.1 Target Variables：'LN.prRLNM'

3.2 the total characteristic variable name

'Age','Sex','BMI','Tumor.border','Aspect.ratio','Ingredients','Internal.echo.pattern','Internal.echo.homogeneous','Hyperechoic','Tumor.internal.vascularization','Tumor.Peripheral.blood.flow','Size','location','Location','Mulifocality','Hashimoto','ETE','prelaryngeal.LNM','pretracheal.LNM','IPLNM','TCLNM','age','size','T.staging','prelaryngeal.LNMR','prelaryngeal.NLNM','pretracheal.LNMR',

'pretracheal.NLNM','IPLNMR','IPNLNM','TCLNMR','TCNLNM'

3.3 Removing variance characteristics

'Age','Sex','Aspect.ratio','Internal.echo.homogeneous','Size','location','Mulifocality'**,**'Hashimoto','pretracheal.LNM','IPLNM','TCLNM'

3.4Univariate Feature Selection

'prelaryngeal.LNM','pretracheal.LNM','IPLNM','TCLNM','T.staging','prelaryngeal.LNMR','pretracheal.LNMR','IPLNMR','TCLNMR','TCNLNM'

3.5 Recursive Feature Elimination (RFE)

'prelaryngeal.LNM','pretracheal.LNM','IPLNM','TCLNM','prelaryngeal.NLNM','pretracheal.NLNM','IPLNMR','IPNLNM','TCLNMR','TCNLNM'

3.6Recursive Feature Elimination with Cross-Validation (RFE-CV)

'ETE','prelaryngeal.LNM','pretracheal.LNM','IPLNM','bmi','T.staging','prelaryngeal.LNMR','prelaryngeal.NLNM','pretracheal.LNMR','pretracheal.NLNM',

3.7L1-based Feature Selection

'BMI','Tumor.border','ETE','prelaryngeal.LNM','TCLNM','size','T.stage','prelaryngeal.NLNM','pretracheal.NLNM','IPLNMR','IPNLNM','TCLNMR','TCNLNM'

3.8Tree-based Feature Selection

'Tumor.border','Hyperechoic','Location','IPLNM','age','size','pretracheal.LNMR','pretracheal.NLNM','IPLNMR','IPNLNM','TCLNMR','TCNLNM'

4.The formula of calculator network

def index(request):

return render(request, "index.html")

@csrf_exempt

def js1(request):

data = json.loads(request.body)

res=0.04*data['value']+0.04*data['value2']+0.06*data['value3']+0.09*data['value4']+0.07*data['value5']+0.17*data['value7']+0.15*float(data['value8'])+0.12*float(data['value10'])

if float(data['value6'])>5:

res=res+0.11

else:

res=res+0.11*float(data['value6'])/5

if float(data['value8'])>5:

res=res+0.15

else:

res=res+0.15*float(data['value9'])/5

return HttpResponse(round(res*100,2))

5..XGBoost optimal parameter Settings

'eta': 0.027825594022071243,'n_estimators':333,'gamma':0.89,'max_depth':3, 'min_child_weight':0,'colsample_bytree':0.3,'colsample_bylevel': 0.0, 'subsample': 0.11111111111, 'reg_lambda': 0.2, 'reg_alpha': 0
