# Supplementary material for: Machine learning and Shapley Additive exPlanations to predict metastasis of lymph nodes posterior to the recurrent laryngeal nerve in cN0 papillary thyroid carcinoma
Source: Front Oncol. 2026 Jan 7;15:1673332. doi: 10.3389/fonc.2025.1673332 (PMC12819197; doi:10.3389/fonc.2025.1673332)
Supplement: Supplementary file 3 [file Table3.docx]

Supplementary Material 3

## 1.1Univariate analysis.R.md

install.packages("tidyverse")

install.packages("caret")

install.packages("dplyr")

install.packages("AER")

install.packages("table1")

install.packages("boot")

library(table1)

library(boot)

library("dplyr")

library("AER")

install.packages("gtsummary")

install.packages("tidyverse")

install.packages("kableExtra")

library(dplyr)

library(gtsummary)

library(tidyverse)

library(kableExtra)

# 读取数据

data <- read.csv("/Users/zj/Desktop/4.机器学习/三、喉返后/0.数据/1.总.csv")

# 分类变量

categorical_variables <- c(

"Age","Sex","Size","BMI","Tumor.border","Aspect.ratio","Ingredients","Internal.echo.pattern","Internal.echo.homogeneous","Hyperechoic","Tumor.internal.vascularization","Tumor.Peripheral.blood.flow","location","Location","Mulifocality","Hashimoto","ETE","T.staging","prelaryngeal.LNM","pretracheal.LNM","IPLNM","TCLNM","LN.prRLNM")

# 数值变量

numeric_variables <- c(

"age","bmi","size","prelaryngeal.LNMR","prelaryngeal.NLNM","pretracheal.LNMR","pretracheal.NLNM","IPLNMR","IPNLNM","TCLNMR","TCNLNM","LN.prRLNM"

)

# 创建空的摘要表

summary_table <- data.frame(变量名称 = character(), 类别 = character(), 数量百分比 = character(), stringsAsFactors = FALSE)

# 处理分类变量

for (variable in categorical_variables) {

counts <- table(data[[variable]])

if (length(counts) > 0) {

total_count <- sum(counts)

variable_values <- data.frame(变量名称 = variable, 类别 = names(counts), 数量百分比 = paste(counts, "(", round(counts / total_count * 100,3), "%)", sep = ""), stringsAsFactors = FALSE)

summary_table <- rbind(summary_table, variable_values)

}

}

# 处理数值变量

for (variable in numeric_variables) {

mean_value <- mean(data[[variable]], na.rm = TRUE)

sd_value <- sd(data[[variable]], na.rm = TRUE)

summary_table <- rbind(summary_table, data.frame(变量名称 = variable, 类别 = "均值 ± 标准差", 数量百分比 = paste(mean_value, "±", sd_value), stringsAsFactors = FALSE))

}

# 导出结果为 CSV 文件

write.csv(summary_table, file = "/Users/zj/Desktop/4.机器学习/三、喉返后/0.数据/结果/1.r语言分析结果/1.TABLE1.csv", row.names = FALSE)

print("摘要表格已成功导出为三线表.csv文件。")

# 加载所需的包

library(pROC)

# 计算ROC曲线

roc_obj <- roc(data$LN.prRLNM, data$size)

# 根据最大Youden指数选择最佳cut-off值

best_cutoff <- coords(roc_obj, "best", ret="threshold")

# 打印最佳cut-off值

print(best_cutoff)

library(table1)

library(boot)

library(dplyr)

# 将缺失值替换为"N/A"

data_filled <-data%>%

mutate_all(~if_else(is.na(.), "N/A", as.character(.)))

# 打印填充后的数据框

print(data_filled)

pvalue <- function(x, ...) {

y <- unlist(x)

g <- factor(rep(1:length(x), times=sapply(x, length)))

if (is.numeric(y)) {

p <- t.test(y ~ g)$p.value

} else {

p <- chisq.test(table(y, g))$p.value

}

c("", sub("<", "&lt;", format.pval(p, digits=3, eps=0.001)))

}

table1(~Sex+Age+Size+BMI+Tumor.border+Aspect.ratio+Ingredients+Internal.echo.pattern+Internal.echo.homogeneous+Hyperechoic+Tumor.internal.vascularization+Tumor.Peripheral.blood.flow+location+Location+Mulifocality+Hashimoto+ETE+T.staging+prelaryngeal.LNM+pretracheal.LNM+IPLNM+TCLNM|

LN.prRLNM,data=data_filled,

overall = F,

extra.col = list('p-value'=pvalue),

topclass = "Rtable1-zebra")

# 将目标变量转换为因子类型

data$LN.prRLNM <- as.factor(data$LN.prRLNM)

# 定义自定义函数，计算p值、95%CI、均值和标准差

pvalue_ci <- function(x, y) {

if (is.numeric(x)) {

if (length(unique(y)) == 2) {

# 删除缺失值

x_no_na <- x[!is.na(x)]

y_no_na <- y[!is.na(x)]

# 两组样本的独立样本t检验

result <- t.test(x_no_na ~ y_no_na)

p <- format(result$p.value, digits = 3)

ci <- paste0("[", format(result$conf.int[1], digits = 3), ", ", format(result$conf.int[2], digits = 3), "]")

mean_sd <- paste0("Mean ± SD: ",

format(mean(x_no_na[y_no_na == levels(y_no_na)[1]]), digits = 3), " ± ",

format(sd(x_no_na[y_no_na == levels(y_no_na)[1]]), digits = 3), " | ",

format(mean(x_no_na[y_no_na == levels(y_no_na)[2]]), digits = 3), " ± ",

format(sd(x_no_na[y_no_na == levels(y_no_na)[2]]), digits = 3))

} else {

# 删除缺失值

x_no_na <- x[!is.na(x)]

y_no_na <- y[!is.na(x)]

# 两组样本的Mann-Whitney U检验

result <- wilcox.test(x_no_na ~ y_no_na)

p <- format(result$p.value, digits = 3)

ci <- ""

mean_sd <- ""

}

} else {

p <- ""

ci <- ""

mean_sd <- ""

}

c(p, ci, mean_sd)

}

# 创建一个空的结果表格

result_table <- data.frame(Variable = character(),

p_value = numeric(),

CI = character(),

Mean_SD = character(),

stringsAsFactors = FALSE)

# 循环计算每个数值变量的p值、95%CI、均值和标准差

for (var in c("bmi", "age","size","prelaryngeal.LNMR","prelaryngeal.NLNM","pretracheal.LNMR","pretracheal.NLNM","IPLNMR","IPNLNM","TCLNMR","TCNLNM")) {

pvalue_ci_result <- pvalue_ci(data[[var]], data$LN.prRLN)

result_table <- rbind(result_table, data.frame(Variable = var,

p_value = pvalue_ci_result[1],

CI = pvalue_ci_result[2],

Mean_SD = pvalue_ci_result[3],

stringsAsFactors = FALSE))

}

# 导出结果为CSV文件

write.csv(result_table, file = "/Users/zj/Desktop/4.机器学习/三、喉返后/0.数据/结果/1.r语言分析结果/1.TABLE1数值变量的单因素.csv", row.names = FALSE)

print("SingleFactorAnalysis.csv文件已成功导出。")

## Multivariate analysis.R.md

install.packages("survival")

install.packages('rrtable')

install.packages('magrittr')

install.packages("ggplot")

install.packages("dplyr")

install.packages("AER")

library("dplyr")

library("AER")

library(openxlsx)

library(survival)

library(rrtable)

library(ggplot2)

A<-fit1<-glm(LN.prRLNM~Age+Sex+Tumor.border+Tumor.internal.vascularization+Tumor.Peripheral.blood.flow+Size+Mulifocality+ETE+T.staging+prelaryngeal.LNM+pretracheal.LNM+IPLNM+TCLNM,data=data,family = binomial())

summary(A)

coefficients(A)

exp(coefficients(A))

exp(confint(A))

coef<-summary(A)$coefficients[,1]

se<-summary(A)$coefficients[,2]

pvalue<-summary(A)$coefficients[,4]

Results<-cbind(exp(coef),exp(coef-1.96*se),exp(coef+1.96*se),pvalue)

dimnames(Results)[[2]]<-c("OR","LL","UL","p value")

Results

Results=Results[,]

View(Results)

table2docx(Results, add.rownames = FALSE)

library(ggplot2)

# 提取模型的系数

fit1 <- glm(LN.prRLNM ~ Age + Sex + Tumor.border + Tumor.internal.vascularization + Tumor.Peripheral.blood.flow + Size + Mulifocality + ETE + T.staging + prelaryngeal.LNM + pretracheal.LNM + IPLNM + TCLNM, data = data, family = binomial())

coefficients <- coef(fit1)

# 创建系数数据框

coef_df <- data.frame(

variable = names(coefficients),

coefficient = coefficients,

odds_ratio = exp(coefficients),

p_value = summary(fit1)$coefficients[, "Pr(>|z|)"],

ci_lower = exp(confint(fit1)[, 1]), # 使用指数化的置信区间

ci_upper = exp(confint(fit1)[, 2]) # 使用指数化的置信区间

)

# 将(Intercept)标签改为Intercept

coef_df$variable[coef_df$variable == "(Intercept)"] <- "Intercept"

# 手动设置变量顺序并反转

variable_order <- c("Intercept", "Age", "Sex", "Tumor.border", "Tumor.internal.vascularization", "Tumor.Peripheral.blood.flow", "Size", "Mulifocality", "ETE", "T.staging", "prelaryngeal.LNM", "pretracheal.LNM", "IPLNM", "TCLNM")

coef_df$variable <- factor(coef_df$variable, levels = rev(variable_order))

# 创建森林图

forest_plot <- ggplot(coef_df, aes(x = odds_ratio, y = variable)) +

geom_errorbarh(aes(xmin = ci_lower, xmax = ci_upper), height = 0.2, color = "black") +

geom_point(aes(color = p_value < 0.05), size = 3) +

geom_vline(xintercept = 1, linetype = "dashed", color = "gray") +

geom_text(aes(label = paste(round(odds_ratio, 3), " (", round(ci_lower, 3), " - ", round(ci_upper, 3), ")", sep = ""), x = -10, hjust = -0.1), size = 3.5) +

geom_text(aes(label = paste("p =", round(p_value, 3)), x = 10, hjust = 1.1), size = 3.5) +

coord_cartesian(xlim = c(-12, 12)) +

scale_color_manual(values = c("black", "red"), labels = c("p >= 0.05", "p < 0.05")) +

labs(x = "Metastasis of LN.prRLN Odds Ratio", y = "Variable") +

theme_minimal()

# 显示森林图

print(forest_plot)

library(ggplot2)

# 提取模型的系数

fit1 <- glm(LN.prRLNM ~ Age + Sex + Tumor.border + Tumor.internal.vascularization + Tumor.Peripheral.blood.flow + Size + Mulifocality + ETE + T.staging + prelaryngeal.LNM + pretracheal.LNM + IPLNM + TCLNM, data = data, family = binomial())

coefficients <- coef(fit1)

# 创建系数数据框

coef_df <- data.frame(

variable = names(coefficients),

coefficient = coefficients,

odds_ratio = exp(coefficients),

p_value = summary(fit1)$coefficients[, "Pr(>|z|)"],

ci_lower = exp(confint(fit1)[, 1]), # 使用指数化的置信区间

ci_upper = exp(confint(fit1)[, 2]) # 使用指数化的置信区间

)

# 将数据框按照Odds Ratio值由小到大排序

coef_df <- coef_df[order(coef_df$odds_ratio), ]

coef_df$variable <- factor(coef_df$variable, levels = coef_df$variable) # 按照排序后的顺序设置变量因子

# 创建森林图

forest_plot <- ggplot(coef_df, aes(x = odds_ratio, y = variable)) +

geom_errorbarh(aes(xmin = ci_lower, xmax = ci_upper), height = 0.2, color = "black") +

geom_point(aes(color = p_value < 0.05), size = 3) +

geom_vline(xintercept = 1, linetype = "dashed", color = "gray") +

geom_text(aes(label = paste(round(odds_ratio, 3), " (", round(ci_lower, 3), " - ", round(ci_upper, 3), ")", sep = ""), x = -10, hjust = -0.1), size = 3.5) +

geom_text(aes(label = paste("p =", round(p_value, 3)), x = 10, hjust = 1.1), size = 3.5) +

coord_cartesian(xlim = c(-12, 12)) +

scale_color_manual(values = c("black", "red"), labels = c("p >= 0.05", "p < 0.05")) +

labs(x = "Metastasis of LN.prRLN Odds Ratio", y = "Variable") +

theme_minimal()

# 显示森林图

print(forest_plot)

## Nomogram.R.md

#install.packages("foreign")

#install.packages("rms")

library(foreign)

library(rms)

data$Tumor.border<-factor(data$Tumor.border,levels = c(0,1,2),labels = c("smooth/borderless","irregular-shape/lsharpobed","extrandular-invasion"))

data$prelaryngeal.LNM<-factor(data$prelaryngeal.LNM,levels = c(0,1),labels = c("No", "Yes"))

data$pretracheal.LNM<-factor(data$pretracheal.LNM,levels = c(0,1),labels = c("No", "Yes"))

data$IPLNM<-factor(data$IPLNM,levels = c(0,1),labels = c("No", "Yes"))

data$TCLNM<-factor(data$TCLNM,levels = c(0,1),labels = c("No", "Yes"))

###4.1.2整合数据

x<-as.data.frame(data)

dd<-datadist(data)

options(datadist='dd')

###4.2logistic回归比GLM好用

fit1<-lrm(LN.prRLNM~Tumor.border+T.staging+prelaryngeal.LNM+pretracheal.LNM+IPLNM+TCLNM

,data=data,x=T,y=T)

fit1

summary(fit1)#可以直接给到一些结果很好

nom1 <- nomogram(fit1, fun=plogis, fun.at=c(.001, .01, .05, seq(.1,.9, by=.1), .95, .99, .999),

lp=F, funlabel="Metastasis of LN.prRLN")

plot(nom1)

###4.4验证曲线

cal1 <- calibrate(fit1, method = 'boot', B = 1000)

plot(cal1, xlim = c(0, 1.0), ylim = c(0, 1.0))

library(foreign)

library(rms)

# 设置绘图参数

par(mar = c(1, 2, 2, 2)) # 调整绘图边距

# 创建 nomogram

nom1 <- nomogram(fit1, fun = plogis, fun.at = c(0.001, 0.01, 0.05, seq(0.1, 0.9, by = 0.1), 0.95, 0.99, 0.999),

lp = FALSE, funlabel = "Metastasis of LN.prRLN")

# 绘制 nomogram

plot(nom1, abbreviate = FALSE, col.lines = "blue", col.points = "blue", cex.names = 11, cex.axis = 0.5,

cex.lab = 10, lwd.lines = 16, lwd.funnel = 20)

##4.2C-statistics计算

###4.2.1加载包

library(foreign)

library(rms)

###4.2.2方法1

x<-as.data.frame(val_data)

dd<-datadist(val_data)

options(datadist='dd')

fit1<-lrm(LN.prRLNM~Age+Sex+Tumor.border+Tumor.internal.vascularization+Tumor.Peripheral.blood.flow+Size+Mulifocality+ETE+T.stage+prelaryngeal.LNM+pretracheal.LNM+IPLNM+TCLNM

,data=val_data,x=T,y=T)

fit1

summary(fit1)#直接读取模型中Rank Discrim.参数 C(方法1)

library(pROC)

library(ggplot2)

# 将数据按照7:3的比例随机分为训练集和验证集

# 设置随机数种子，以确保结果可复现

set.seed(123)

index <- 1:nrow(data)

shuffled_index <- sample(index)

tra_ratio <- 0.7 # 训练集比例

val_ratio <- 0.3 # 验证集比例

tra_size <- round(tra_ratio * nrow(data))

val_size <- round(val_ratio * nrow(data))

tra_data <- data[shuffled_index[1:tra_size], ]

val_data <- data[shuffled_index[(tra_size + 1):(tra_size + val_size)], ]

cat("训练集观测数量:", nrow(tra_data), "\n")

cat("验证集观测数量:", nrow(val_data), "\n")

# 构建模型

fit1 <- glm(LN.prRLNM ~ Tumor.border + T.staging + prelaryngeal.LNM + pretracheal.LNM + IPLNM + TCLNM,

data = tra_data, family = binomial())

# 预测

probs <- predict(fit1, newdata = val_data, type = "response")

response <- val_data$LN.prRLNM

# 创建ROC对象

roc_obj <- roc(response, probs)

# 提取ROC曲线的坐标点

roc_data <- coords(roc_obj, "all")

# 绘制ROC曲线

ggplot(roc_data, aes(x = 1 - specificity, y = sensitivity)) +

geom_line(color = "steelblue", size = 1) +

geom_abline(slope = 1, intercept = 0, linetype = "dashed", color = "darkred") +

labs(title = "ROC for Metastasis of LN.prRLN Nomogram Prediction(Validation set)", x = "False Positive Rate", y = "True Positive Rate") +

theme_minimal() +

theme(legend.position = "none") + # 移除图例

annotate("text", x = 0.7, y = 0.3, label = paste("AUC =", round(auc(roc_obj), 3)), size = 4, color = "black")

# 加载必要的库

library(pROC)

library(ggplot2)

# 读取数据

data <- read.csv("/Users/zj/Desktop/4.机器学习/三、喉返后/0.数据/1.总P插补缺失值后.csv")

# 将数据按照7:3的比例随机分为训练集和验证集

# 设置随机数种子,以确保结果可复现

set.seed(123)

index <- 1:nrow(data)

shuffled_index <- sample(index)

tra_ratio <- 0.7 # 训练集比例

tra_size <- round(tra_ratio * nrow(data))

tra_data <- data[shuffled_index[1:tra_size], ]

val_data <- data[shuffled_index[(tra_size + 1):nrow(data)], ]

cat("训练集观测数量:", nrow(tra_data), "\n")

cat("验证集观测数量:", nrow(val_data), "\n")

# 构建模型

fit1 <- glm(LN.prRLNM ~ Tumor.border + T.staging + prelaryngeal.LNM + pretracheal.LNM + IPLNM + TCLNM,

data = tra_data, family = binomial())

# 预测

probs <- predict(fit1, newdata = val_data, type = "response")

response <- val_data$LN.prRLNM

# 创建ROC对象

roc_obj <- roc(response, probs)

# 提取ROC曲线的坐标点

roc_data <- coords(roc_obj, "all")

# 绘制ROC曲线

ggplot(roc_data, aes(x = 1 - specificity, y = sensitivity)) +

geom_line(color = "steelblue", size = 1) +

geom_abline(slope = 1, intercept = 0, linetype = "dashed", color = "darkred") +

labs(title = "ROC for Metastasis of LN.prRLN Nomogram Prediction(Validation set)", x = "False Positive Rate", y = "True Positive Rate") +

theme_minimal() +

theme(legend.position = "none") + # 移除图例

annotate("text", x = 0.7, y = 0.3, label = paste("AUC =", round(auc(roc_obj), 3)), size = 4, color = "black")

# 定义净收益计算函数

net_benefit <- function(probs, outcome, threshold) {

tp <- sum(outcome == 1 & probs >= threshold)

fp <- sum(outcome == 0 & probs >= threshold)

total_population <- length(outcome)

if (total_population == 0) {

return(0)

}

net_benefit <- (tp / total_population) - ((fp / total_population) * (threshold / (1 - threshold)))

return(net_benefit)

}

# 计算不同阈值下的净收益

thresholds <- seq(0, 0.6, 0.01)

net_benefits <- sapply(thresholds, function(x) net_benefit(probs, response, x))

# 计算所有人都进行干预时的净收益

net_benefit_all <- sapply(thresholds, function(x) net_benefit(rep(1, length(response)), response, x))

# 计算没有人进行干预时的净收益

net_benefit_none <- rep(0, length(thresholds))

# 绘制DCA曲线

net_benefit_df <- data.frame(thresholds, net_benefits, net_benefit_all, net_benefit_none)

ggplot(net_benefit_df, aes(x = thresholds)) +

geom_line(aes(y = net_benefits, color = "Net Benefit"), size = 1) +

geom_line(aes(y = net_benefit_none, color = "None"), size = 0.8, linetype = "solid") +

geom_line(aes(y = net_benefit_all, color = "All"), size = 0.8, linetype = "dashed") +

labs(title = "DCA for Metastasis of LN.prRLN Nomogram Prediction",

x = "Threshold Probability", y = "Net Benefit") +

scale_x_continuous(limits = c(0, 0.4)) +

scale_y_continuous(limits = c(-0.025, 0.1)) +

scale_color_manual(name = "Legend",

values = c("Net Benefit" = "steelblue", "None" = "darkred", "All" = "grey")) +

theme_bw()

# 加载必要的库

library(pROC)

library(ggplot2)

# 读取数据

train_data <- read.csv("/Users/zj/Desktop/4.机器学习/三、喉返后/0.数据/1.总P矫正.csv")

test_data <- read.csv("/Users/zj/Desktop/4.机器学习/三、喉返后/0.数据/2.T总P.csv")

# 将训练数据按照7:3的比例随机分为训练集和验证集

# 设置随机数种子,以确保结果可复现

set.seed(123)

index <- 1:nrow(train_data)

shuffled_index <- sample(index)

tra_ratio <- 0.7 # 训练集比例

tra_size <- round(tra_ratio * nrow(train_data))

tra_data <- train_data[shuffled_index[1:tra_size], ]

val_data <- train_data[shuffled_index[(tra_size + 1):nrow(train_data)], ]

cat("训练集观测数量:", nrow(tra_data), "\n")

cat("验证集观测数量:", nrow(val_data), "\n")

cat("测试集观测数量:", nrow(test_data), "\n")

# 构建模型

fit1 <- glm(LN.prRLNM ~ Tumor.border + T.stage + prelaryngeal.LNM + pretracheal.LNM + IPLNM + TCLNM,

data = tra_data, family = binomial())

# 预测概率

train_probs <- predict(fit1, newdata = tra_data, type = "response")

val_probs <- predict(fit1, newdata = val_data, type = "response")

test_probs <- predict(fit1, newdata = test_data, type = "response")

train_response <- tra_data$LN.prRLNM

val_response <- val_data$LN.prRLNM

test_response <- test_data$LN.prRLNM

# 创建ROC对象

train_roc <- roc(train_response, train_probs)

val_roc <- roc(val_response, val_probs)

test_roc <- roc(test_response, test_probs)

# 提取ROC曲线的坐标点

train_roc_data <- coords(train_roc, "all")

val_roc_data <- coords(val_roc, "all")

test_roc_data <- coords(test_roc, "all")

# 绘制ROC曲线

ggplot() +

geom_line(data = train_roc_data, aes(x = 1 - specificity, y = sensitivity), color = "darkorange", size = 0.8) +

geom_line(data = val_roc_data, aes(x = 1 - specificity, y = sensitivity), color = "steelblue", size = 0.8) +

geom_line(data = test_roc_data, aes(x = 1 - specificity, y = sensitivity), color = "darkgreen", size = 0.8) +

geom_abline(slope = 1, intercept = 0, linetype = "dashed", color = "darkred") +

labs(title = "ROC for Metastasis of LN.prRLN Nomogram Prediction",

x = "False Positive Rate", y = "True Positive Rate") +

theme_minimal() +

theme(legend.position = "none") +

annotate("text", x = 0.7, y = 0.4, label = paste("Train set AUC =", round(auc(train_roc), 3)), size = 4, color = "darkorange") +

annotate("text", x = 0.7, y = 0.3, label = paste("Validation set AUC =", round(auc(val_roc), 3)), size = 4, color = "steelblue") +

annotate("text", x = 0.7, y = 0.2, label = paste("Test set AUC =", round(auc(test_roc), 3)), size = 4, color = "darkgreen")

# 定义净收益计算函数

net_benefit <- function(probs, outcome, threshold) {

tp <- sum(outcome == 1 & probs >= threshold)

fp <- sum(outcome == 0 & probs >= threshold)

total_population <- length(outcome)

if (total_population == 0) {

return(0)

}

net_benefit <- (tp / total_population) - ((fp / total_population) * (threshold / (1 - threshold)))

return(net_benefit)

}

# 计算不同阈值下的净收益

thresholds <- seq(0, 0.6, 0.01)

train_net_benefits <- sapply(thresholds, function(x) net_benefit(train_probs, train_response, x))

val_net_benefits <- sapply(thresholds, function(x) net_benefit(val_probs, val_response, x))

test_net_benefits <- sapply(thresholds, function(x) net_benefit(test_probs, test_response, x))

# 计算所有人都进行干预时的净收益

all_net_benefit <- sapply(thresholds, function(x) net_benefit(rep(1, length(val_response)), val_response, x))

# 计算没有人进行干预时的净收益

none_net_benefit <- rep(0, length(thresholds))

# 绘制DCA曲线

dca_data <- data.frame(thresholds, train_net_benefits, val_net_benefits, test_net_benefits, all_net_benefit, none_net_benefit)

ggplot(dca_data, aes(x = thresholds)) +

geom_line(aes(y = train_net_benefits, color = "Train Net Benefit"), size = 0.8) +

geom_line(aes(y = val_net_benefits, color = "Validation Net Benefit"), size = 0.8) +

geom_line(aes(y = test_net_benefits, color = "Test Net Benefit"), size = 0.8) +

geom_line(aes(y = none_net_benefit, color = "None"), size = 0.6, linetype = "solid") +

geom_line(aes(y = all_net_benefit, color = "All"), size = 0.6, linetype = "dashed") +

labs(title = "DCA for Metastasis of LN.prRLN Nomogram Prediction",

x = "Threshold Probability", y = "Net Benefit") +

scale_x_continuous(limits = c(0, 0.5)) +

scale_y_continuous(limits = c(-0.025, 0.15)) +

scale_color_manual(name = "Legend",

values = c("Train Net Benefit" = "darkorange", "Validation Net Benefit" = "steelblue", "Test Net Benefit" = "darkgreen", "None" = "darkred", "All" = "grey")) +

theme_bw()

## 2.Analysis of the data set.Py.md

#安装包

!pip install -U scikit-learn

##安装pandas的包用于数据的读取与纳入

!pip install pandas

#1、三组数据库的差异表现

import pandas as pd

import numpy as np

from scipy.stats import chi2_contingency, f_oneway

# 读取数据

data1 = pd.read_csv("/Users/zj/Desktop/4.机器学习/三、喉返后/0.数据/1.总P.csv")

data2 = pd.read_csv("/Users/zj/Desktop/4.机器学习/三、喉返后/0.数据/2.T总P.csv")

# 分类变量

categorical_variables = [

"Age","Sex","BMI","Tumor.border","Aspect.ratio","Ingredients","Internal.echo.pattern","Internal.echo.homogeneous","Hyperechoic","Tumor.internal.vascularization",

"Tumor.Peripheral.blood.flow","Size","location","Location","Mulifocality","Hashimoto","ETE","T.staging",

"prelaryngeal.LNM","pretracheal.LNM",

"IPLNM","TCLNM","LN.prRLNM"

]

# 数值变量

numeric_variables = [

"TCLNMR","TCNLNM", "IPLNMR","IPNLNM","pretracheal.LNMR","pretracheal.NLNM","prelaryngeal.LNMR","prelaryngeal.NLNM","size","bmi","age",

]

# 分割训练集和验证集

train_data = data1.sample(frac=0.7, random_state=123)

val_data = data1.drop(train_data.index)

# 初始化 summary_table

summary_table = []

# 确保分类变量为字符串

for variable in categorical_variables:

train_data[variable] = train_data[variable].astype(str)

val_data[variable] = val_data[variable].astype(str)

data2[variable] = data2[variable].astype(str)

# 处理分类变量

for variable in categorical_variables:

if variable in train_data.columns:

# 训练集

train_counts = train_data[variable].value_counts().to_dict()

train_total = train_data[variable].count()

train_summary = "; ".join([f"{k}({v}, {v/train_total*100:.3f}%)" for k, v in train_counts.items()])

# 内验证集

val_counts = val_data[variable].value_counts().to_dict()

val_total = val_data[variable].count()

val_summary = "; ".join([f"{k}({v}, {v/val_total*100:.3f}%)" for k, v in val_counts.items()])

# 外验证集

ext_counts = data2[variable].value_counts().to_dict()

ext_total = data2[variable].count()

ext_summary = "; ".join([f"{k}({v}, {v/ext_total*100:.3f}%)" for k, v in ext_counts.items()])

# 卡方检验计算 p 值

combined_counts = pd.DataFrame({

"train": train_data[variable].value_counts(normalize=True),

"val": val_data[variable].value_counts(normalize=True),

"ext": data2[variable].value_counts(normalize=True)

}).fillna(0)

chi2, p_value, _, _ = chi2_contingency(combined_counts.T.values)

summary_table.append({

"变量名称": variable,

"训练集": train_summary,

"内验证集": val_summary,

"外验证集": ext_summary,

"p值": round(p_value, 3)

})

# 处理数值变量

for variable in numeric_variables:

if variable in train_data.columns:

# 训练集

train_mean = train_data[variable].mean()

train_sd = train_data[variable].std()

train_summary = f"{train_mean:.3f} ± {train_sd:.3f}"

# 内验证集

val_mean = val_data[variable].mean()

val_sd = val_data[variable].std()

val_summary = f"{val_mean:.3f} ± {val_sd:.3f}"

# 外验证集

ext_mean = data2[variable].mean()

ext_sd = data2[variable].std()

ext_summary = f"{ext_mean:.3f} ± {ext_sd:.3f}"

# ANOVA 计算 p 值

f_stat, p_value = f_oneway(train_data[variable].dropna(), val_data[variable].dropna(), data2[variable].dropna())

summary_table.append({

"变量名称": variable,

"训练集": train_summary,

"内验证集": val_summary,

"外验证集": ext_summary,

"p值": round(p_value, 3)

})

# 转换为 DataFrame 并导出 CSV

summary_df = pd.DataFrame(summary_table)

summary_df.to_csv("/Users/zj/Desktop/4.机器学习/三、喉返后/0.数据/结果/1.r语言分析结果/2.table2.csv", index=False, encoding='utf-8-sig')

##1、2三组数据集的单因素分析

import pandas as pd

import numpy as np

from scipy.stats import chi2_contingency, f_oneway

# 读取数据

data1 = pd.read_csv("/Users/zj/Desktop/4.机器学习/三、喉返后/0.数据/1.总P.csv")

data2 = pd.read_csv("/Users/zj/Desktop/4.机器学习/三、喉返后/0.数据/2.T总P.csv")

# 分类变量

categorical_variables = [

"Age", "Sex", "BMI", "Tumor.border", "Aspect.ratio", "Ingredients", "Internal.echo.pattern",

"Internal.echo.homogeneous", "Hyperechoic", "Tumor.internal.vascularization", "Tumor.Peripheral.blood.flow",

"Size", "location", "Location", "Mulifocality", "Hashimoto", "ETE", "T.staging", "prelaryngeal.LNM",

"pretracheal.LNM", "IPLNM", "TCLNM", "LN.prRLNM"

]

# 数值变量

numeric_variables = [

"TCLNMR", "TCNLNM", "IPLNMR", "IPNLNM", "pretracheal.LNMR", "pretracheal.NLNM", "prelaryngeal.LNMR",

"prelaryngeal.NLNM", "size", "bmi", "age"

]

# 分割训练集和验证集

train_data = data1.sample(frac=0.7, random_state=123)

val_data = data1.drop(train_data.index)

# 初始化 summary_table

summary_table = []

# 确保分类变量为字符串

for variable in categorical_variables:

train_data[variable] = train_data[variable].astype(str)

val_data[variable] = val_data[variable].astype(str)

data2[variable] = data2[variable].astype(str)

# 函数：获取每个变量在yes和no组中的数目及百分比

def get_counts_percentages(data, variable, target_variable):

yes_counts = data[data[target_variable] == 'Yes'][variable].value_counts()

no_counts = data[data[target_variable] == 'No'][variable].value_counts()

total_yes = yes_counts.sum()

total_no = no_counts.sum()

yes_summary = {k: f"{v} ({v/total_yes*100:.3f}%)" for k, v in yes_counts.items()}

no_summary = {k: f"{v} ({v/total_no*100:.3f}%)" for k, v in no_counts.items()}

return yes_summary, no_summary

# 处理分类变量

for variable in categorical_variables:

if variable in train_data.columns:

# 训练集

train_yes_summary, train_no_summary = get_counts_percentages(train_data, variable, 'LN.prRLNM')

# 内验证集

val_yes_summary, val_no_summary = get_counts_percentages(val_data, variable, 'LN.prRLNM')

# 外验证集

ext_yes_summary, ext_no_summary = get_counts_percentages(data2, variable, 'LN.prRLNM')

# 卡方检验计算 p 值

combined_counts_train = pd.crosstab(train_data[variable], train_data['LN.prRLNM'])

chi2_train, train_p_value, _, _ = chi2_contingency(combined_counts_train)

combined_counts_val = pd.crosstab(val_data[variable], val_data['LN.prRLNM'])

chi2_val, val_p_value, _, _ = chi2_contingency(combined_counts_val)

combined_counts_ext = pd.crosstab(data2[variable], data2['LN.prRLNM'])

chi2_ext, ext_p_value, _, _ = chi2_contingency(combined_counts_ext)

summary_table.append({

"变量名称": variable,

"训练集:yes": "; ".join([f"{k}({v})" for k, v in train_yes_summary.items()]),

"训练集:no": "; ".join([f"{k}({v})" for k, v in train_no_summary.items()]),

"训练集p值": round(train_p_value, 3),

"内验证集:yes": "; ".join([f"{k}({v})" for k, v in val_yes_summary.items()]),

"内验证集:no": "; ".join([f"{k}({v})" for k, v in val_no_summary.items()]),

"内验证集p值": round(val_p_value, 3),

"外验证集:yes": "; ".join([f"{k}({v})" for k, v in ext_yes_summary.items()]),

"外验证集:no": "; ".join([f"{k}({v})" for k, v in ext_no_summary.items()]),

"外验证集p值": round(ext_p_value, 3)

})

# 处理数值变量

for variable in numeric_variables:

if variable in train_data.columns:

# 训练集

train_yes = train_data[train_data['LN.prRLNM'] == 'Yes'][variable].dropna()

train_no = train_data[train_data['LN.prRLNM'] == 'No'][variable].dropna()

train_yes_mean = train_yes.mean()

train_yes_sd = train_yes.std()

train_no_mean = train_no.mean()

train_no_sd = train_no.std()

train_summary_yes = f"{train_yes_mean:.3f} ± {train_yes_sd:.3f}"

train_summary_no = f"{train_no_mean:.3f} ± {train_no_sd:.3f}"

# 内验证集

val_yes = val_data[val_data['LN.prRLNM'] == 'Yes'][variable].dropna()

val_no = val_data[val_data['LN.prRLNM'] == 'No'][variable].dropna()

val_yes_mean = val_yes.mean()

val_yes_sd = val_yes.std()

val_no_mean = val_no.mean()

val_no_sd = val_no.std()

val_summary_yes = f"{val_yes_mean:.3f} ± {val_yes_sd:.3f}"

val_summary_no = f"{val_no_mean:.3f} ± {val_no_sd:.3f}"

# 外验证集

ext_yes = data2[data2['LN.prRLNM'] == 'Yes'][variable].dropna()

ext_no = data2[data2['LN.prRLNM'] == 'No'][variable].dropna()

ext_yes_mean = ext_yes.mean()

ext_yes_sd = ext_yes.std()

ext_no_mean = ext_no.mean()

ext_no_sd = ext_no.std()

ext_summary_yes = f"{ext_yes_mean:.3f} ± {ext_yes_sd:.3f}"

ext_summary_no = f"{ext_no_mean:.3f} ± {ext_no_sd:.3f}"

# ANOVA 计算 p 值

_, train_p_value = f_oneway(train_yes, train_no)

_, val_p_value = f_oneway(val_yes, val_no)

_, ext_p_value = f_oneway(ext_yes, ext_no)

summary_table.append({

"变量名称": variable,

"训练集:yes": train_summary_yes,

"训练集:no": train_summary_no,

"训练集p值": round(train_p_value, 3),

"内验证集:yes": val_summary_yes,

"内验证集:no": val_summary_no,

"内验证集p值": round(val_p_value, 3),

"外验证集:yes": ext_summary_yes,

"外验证集:no": ext_summary_no,

"外验证集p值": round(ext_p_value, 3)

})

# 转换为 DataFrame 并导出 CSV

summary_df = pd.DataFrame(summary_table)

summary_df.to_csv("/Users/zj/Desktop/4.机器学习/三、喉返后/0.数据/结果/1.r语言分析结果/2.table2.2.csv", index=False, encoding='utf-8-sig')

## 3.1.ML.Data processing.py

#指定pandas为pd方便后续数据的读取

import pandas as pd

#3、数据规范化

#指定pandas为pd方便后续数据的读取

import pandas as pd

#1、分类变量的编码

data.head

#1.1找出分类型的变量

data_category = data.select_dtypes(include=['object'])

#1.2查看

data_category

data_Number=data.select_dtypes(exclude=['object'])

data_Number

data_Number.columns.values

#1.6整合编码

from sklearn.preprocessing import OrdinalEncoder

# 创建并拟合编码器

encoder = OrdinalEncoder()

encoder.fit(data_category)

# 将分类变量进行编码转换

data_category_enc = pd.DataFrame(encoder.transform(data_category), columns=data_category.columns)

#1.7加载表头

data_category_enc

#1.10将表格拼回去

data_enc=pd.concat([data_category_enc,data_Number],axis=1)

#axis=0为纵向拼接 axis=1是按列拼接

#1.11编码完成

data_enc

#1.12将新的编码后的数据输入文件夹中

data_enc.to_csv('/Users/zj/Desktop/4.机器学习/三、喉返后/0.数据/结果/2.T总编码后.csv')

#2.1分类变量无法使用均质填补，因此使用众数填补（即出现频率最高的数进行填补）

#加载sklearn 的函数

from sklearn.impute import SimpleImputer

#2.2众数填补的缺失值

import pandas as pd

import numpy as np

from sklearn.impute import SimpleImputer

# 创建并拟合填充器

imp = SimpleImputer(missing_values=np.nan, strategy='most_frequent')

data_encImpute = pd.DataFrame(imp.fit_transform(data_enc))

# 设置列名

data_encImpute.columns = data_enc.columns

#2.3整合

data_encImpute

#2.4看之前的变量名字

data_encImpute['prelaryngeal.LNM'].value_counts()

#2.5将插补后的数据保存下来

data_encImpute.to_csv('/Users/zj/Desktop/4.机器学习/三、喉返后/0.数据/结果/2.T总-仅仅用于r的dca2.csv')

#3数值数据校准和归一化

data_scale=data_encImpute

#3.1

target=data_encImpute['LN.prRLNM'].astype(int)

##3.2

target.value_counts()

from sklearn import preprocessing

scaler=preprocessing.MinMaxScaler()

data_scaled=pd.DataFrame(scaler.fit_transform(data_scale))

data_scaled.columns=data_scale.columns

data_scaled

#将矫正后的数据保存下来

data_scaled.to_csv('/Users/zj/Desktop/4.机器学习/三、喉返后/0.数据/2.T总P矫正.csv')

## 3.2.ML.Dimension reduction.py

data=pd.read_csv('/Users/zj/Desktop/4.机器学习/三、喉返后/0.数据/2.T总P矫正.csv')

#1、移除方差特性

from sklearn.feature_selection import VarianceThreshold

sel = VarianceThreshold(threshold=(.8 * (1 - .8)))

data_sel = sel.fit_transform(data)

data_sel

a=sel.get_support(indices=True)

data.iloc[:,a]

data_sel=data.iloc[:,a]

data_sel.info()

#2、单变量特征选择

from sklearn.feature_selection import SelectKBest, chi2

data=pd.read_csv('/Users/zj/Desktop/4.机器学习/三、喉返后/0.数据/1.总P矫正.csv')

data_feature = data[['Age','Sex','BMI','Tumor.border','Aspect.ratio','Ingredients','Internal.echo.pattern','Internal.echo.homogeneous','Hyperechoic',

'Tumor.internal.vascularization','Tumor.Peripheral.blood.flow','Size','location','Location','Mulifocality','Hashimoto','ETE','prelaryngeal.LNM',

'pretracheal.LNM','IPLNM','TCLNM','age','size','T.staging','prelaryngeal.LNMR','prelaryngeal.NLNM','pretracheal.LNMR',

'pretracheal.NLNM','IPLNMR','IPNLNM','TCLNMR','TCNLNM']]

data_feature.shape

data_target=data['LN.prRLNM']

data_target.unique()

set_kit=SelectKBest(chi2,k=10)#选取k值最高的10(5)个元素

data_sel=set_kit.fit_transform(data_feature,data_target)

data_sel.shape

a=set_kit.get_support(indices=True)

data_sel=data_feature.iloc[:,a]

data_sel.info()

#3、RFE

from sklearn.feature_selection import RFE, RFECV

from sklearn.ensemble import RandomForestClassifier

from sklearn.preprocessing import StandardScaler

from sklearn.svm import SVR #知识向量回归模型

from sklearn.model_selection import cross_val_score #知识向量回归模型

data=pd.read_csv('/Users/zj/Desktop/4.机器学习/三、喉返后/0.数据/1.总P矫正.csv')

data_feature = data[['Age','Sex','BMI','Tumor.border','Aspect.ratio','Ingredients','Internal.echo.pattern','Internal.echo.homogeneous','Hyperechoic',

'Tumor.internal.vascularization','Tumor.Peripheral.blood.flow','Size','location','Location','Mulifocality','Hashimoto','ETE','prelaryngeal.LNM',

'pretracheal.LNM','IPLNM','TCLNM','age','size','T.staging','prelaryngeal.LNMR','prelaryngeal.NLNM','pretracheal.LNMR',

'pretracheal.NLNM','IPLNMR','IPNLNM','TCLNMR','TCNLNM']]

data_feature.shape

estimator=SVR(kernel='linear')

sel=RFE(estimator,n_features_to_select=10,step=1)

data_target=data['LN.prRLNM']

data_target.unique()

sel.fit(data_feature,data_target)

a=sel.get_support(indices=True)

data_sel=data_feature.iloc[:,a]

data_sel.info()

#4、RFECV

RFC_ = RandomForestClassifier() # 随机森林

RFC_.fit(data_sel, data_target) # 拟合模型

c = RFC_.feature_importances_ # 特征重要性

print('重要性：')

print(c)

selector = RFECV(RFC_, step=1, cv=10,min_features_to_select=10) # 采用交叉验证cv就是10倍交叉验证，每次排除一个特征，筛选出最优特征

selector.fit(data_sel, data_target)

X_wrapper = selector.transform(data_sel) # 最优特征

score = cross_val_score(RFC_, X_wrapper, data_target, cv=5).mean() # 最优特征分类结果

print(score)

print('最佳数量和排序')

print(selector.support_)

print(selector.n_features_)

print(selector.ranking_)

print(selector.support_)

feature_names = data_sel.columns

selected_features = feature_names[selector.support_]

print(selected_features)

print(selector.ranking_)

import matplotlib.pyplot as plt

plt.figure(figsize=(10, 6))

plt.bar(range(len(selector.ranking_)), selector.ranking_)

plt.xticks(range(len(selector.ranking_)), feature_names, rotation=90)

plt.xlabel('Feature')

plt.ylabel('Ranking')

plt.title('Feature Importance Ranking')

plt.show()

rfecv=RFECV(estimator=RFC_,step=1,cv=StratifiedKFold(2),scoring='accuracy')

rfecv.fit(data,data_target)

data.iloc[:,a]

data_sel=data.iloc[:,a]

#5、L1

from sklearn.feature_selection import SelectFromModel

from sklearn.ensemble import ExtraTreesClassifier

from sklearn.datasets import load_iris

from sklearn.linear_model import LogisticRegression #知识向量回归模型

import numpy as np

import matplotlib.pyplot as plt

from sklearn.linear_model import LogisticRegression

from sklearn.feature_selection import SelectFromModel

from sklearn.model_selection import StratifiedKFold

clf = LogisticRegression()

clf.fit(data_feature, data_target)

model = SelectFromModel(clf, prefit=True)

data_new = model.transform(data_feature)

model.get_support(indices=True)

a=model.get_support(indices=True)

data_features=pd.DataFrame(data_feature)

data_features.columns=data_feature.columns

data_featurenew=data_features.iloc[:,a]

data_featurenew.info()

#6、基于树模型

data=pd.read_csv('/Users/zj/Desktop/4.机器学习/三、喉返后/0.数据/1.总P矫正.csv')

data_feature = data[['Age','Sex','BMI','Tumor.border','Aspect.ratio','Ingredients','Internal.echo.pattern','Internal.echo.homogeneous','Hyperechoic',

'Tumor.internal.vascularization','Tumor.Peripheral.blood.flow','Size','location','Location','Mulifocality','Hashimoto','ETE','prelaryngeal.LNM',

'pretracheal.LNM','IPLNM','TCLNM',

'age','size','T.staging','prelaryngeal.LNMR','prelaryngeal.NLNM','pretracheal.LNMR',

'pretracheal.NLNM','IPLNMR','IPNLNM','TCLNMR','TCNLNM']]

data_target=data['LN.prRLNM']

data_target.unique()#二分类

clf = ExtraTreesClassifier()

clf.fit(data_feature, data_target)

clf.feature_importances_

model=SelectFromModel(clf,prefit=True)

x_new=model.transform(data_feature)

model.get_support(indices=True)

a=model.get_support(indices=True)

data_features=pd.DataFrame(data_feature)

data_features.columns=data_feature.columns

data_featurenew=data_features.iloc[:,a]

data_featurenew

data_featurenew.info()

## 4.1.Train.Development and optimization of prediction models

#1、ROC

import pandas as pd

import matplotlib.pyplot as plt

from sklearn.model_selection import train_test_split, GridSearchCV

from sklearn.metrics import roc_auc_score, roc_curve, accuracy_score, precision_score, recall_score, f1_score, confusion_matrix

from sklearn.linear_model import LogisticRegression

from sklearn.tree import DecisionTreeClassifier

from sklearn.ensemble import RandomForestClassifier, GradientBoostingClassifier

from sklearn.svm import SVC

from sklearn.neighbors import KNeighborsClassifier

from sklearn.naive_bayes import GaussianNB

from sklearn.neural_network import MLPClassifier

from sklearn.preprocessing import MinMaxScaler

import numpy as np

from xgboost import XGBClassifier

# 加载数据

data = pd.read_csv('/Users/zj/Desktop/4.机器学习/三、喉返后/1.矫正后.csv')

# 导入数据

data_feature = data[['Tumor.border','Hyperechoic','Location','IPLNM',

'age','size','pretracheal.LNMR',

'pretracheal.NLNM','IPLNMR','IPNLNM','TCLNMR','TCNLNM']]

data_target = data['LN.prRLNM']

# 数值变量标准化

data_featureNum = data[['age','size','pretracheal.LNMR',

'pretracheal.NLNM','IPLNMR','IPNLNM','TCLNMR','TCNLNM']]

scaler = MinMaxScaler()

data_featureNum = scaler.fit_transform(data_featureNum)

data_featureCata = data[['Tumor.border','Hyperechoic','Location','IPLNM','prelaryngeal.LNM',]]

data_featureCata = np.array(data_featureCata)

# 整合数据

data_feature = np.hstack((data_featureCata, data_featureNum))

# 分为训练集和验证集

class_x_tra, class_x_val, class_y_tra, class_y_val = train_test_split(data_feature, data_target, test_size=0.3, random_state=0)

# 定义模型和参数空间

model_param_grid= {

'Logistic Regression': (LogisticRegression(), {'C': [0.01, 0.1, 1, 10, 100]}),

'Decision Tree': (DecisionTreeClassifier(), {'max_depth': [3, 5, 7, 10], 'min_samples_split': [2, 5, 10]}),

'Random Forest': (RandomForestClassifier(), {'n_estimators': [50, 100, 200], 'max_depth': [3, 5, 7], 'min_samples_split': [2, 5, 10]}),

'Gradient Boosting': (GradientBoostingClassifier(), {'n_estimators': [50, 100, 200], 'learning_rate': [0.01, 0.1, 0.2], 'max_depth': [3, 5, 7]}),

'Support Vector Machine': (SVC(probability=True), {'C': [0.01, 0.1, 1, 10, 100], 'kernel': ['linear', 'rbf']}),

'K-Nearest Neighbors': (KNeighborsClassifier(), {'n_neighbors': [3, 5, 7]}),

'Gaussian Naive Bayes': (GaussianNB(), {}),

'Neural Network': (MLPClassifier(), {'hidden_layer_sizes': [(10,), (20,), (30,)], 'activation': ['relu', 'tanh'], 'solver': ['adam', 'sgd'], 'alpha': [0.0001, 0.001, 0.01], 'learning_rate': ['constant', 'adaptive'], 'learning_rate_init': [0.001, 0.01, 0.1]}),

'XGBoost': (XGBClassifier(use_label_encoder=False, eval_metric='auc'), {'n_estimators': [50, 100, 200],

'max_depth': [5, 7, 10], 'learning_rate': [0.01,0.05, 0.1], 'subsample': [0.7, 0.8, 0.9], 'gamma': [0, 0.1, 0.5]})

}

# 定义颜色列表

colors = ['blue', 'green', 'red', 'purple', 'brown', 'pink', 'gray', 'cyan', 'orange']

# 初始化最佳AUC和最佳模型

best_auc = 0

best_model_name = ''

best_model = None

# 创建评价指标的空列表

train_accuracy_scores = []

train_auc_scores = []

train_precision_scores = []

train_specificity_scores = []

train_sensitivity_scores = []

train_npv_scores = []

train_ppv_scores = []

train_recall_scores = []

train_f1_scores = []

train_fpr_scores = []

# 拟合模型并绘制ROC曲线

plt.figure(figsize=(8, 6))

for (name, (model, param_grid)), color in zip(model_param_grid.items(), colors):

grid_search = GridSearchCV(estimator=model, param_grid=param_grid, cv=5)

grid_search.fit(class_x_tra, class_y_tra)

best_model_temp = grid_search.best_estimator_

# 计算训练集上的预测概率

if hasattr(best_model_temp, 'predict_proba'):

y_train_pred_prob = best_model_temp.predict_proba(class_x_tra)[:, 1]

else:

y_train_pred_prob = best_model_temp.decision_function(class_x_tra)

# 计算AUC值

auc = roc_auc_score(class_y_tra, y_train_pred_prob)

# 如果当前模型的AUC值是最高的，则更新最佳模型和最佳AUC

if auc > best_auc:

best_auc = auc

best_model_name = name

best_model = best_model_temp

# 计算ROC曲线

fpr, tpr, _ = roc_curve(class_y_tra, y_train_pred_prob)

# 绘制ROC曲线

plt.plot(fpr, tpr, color=color, label='%s (AUC = %0.3f)' % (name, auc))

# 计算其他评价指标

train_y_pred = best_model_temp.predict(class_x_tra)

train_accuracy = accuracy_score(class_y_tra, train_y_pred)

train_precision = precision_score(class_y_tra, train_y_pred)

train_cm = confusion_matrix(class_y_tra, train_y_pred)

train_tn, train_fp, train_fn, train_tp = train_cm.ravel()

train_specificity = train_tn / (train_tn + train_fp)

train_sensitivity = recall_score(class_y_tra, train_y_pred)

train_npv = train_tn / (train_tn + train_fn)

train_ppv = train_tp / (train_tp + train_fp)

train_recall = train_sensitivity

train_f1 = f1_score(class_y_tra, train_y_pred)

train_fpr = train_fp / (train_fp + train_tn)

# 将评价指标添加到列表中

train_accuracy_scores.append(train_accuracy)

train_auc_scores.append(auc)

train_precision_scores.append(train_precision)

train_specificity_scores.append(train_specificity)

train_sensitivity_scores.append(train_sensitivity)

train_npv_scores.append(train_npv)

train_ppv_scores.append(train_ppv)

train_recall_scores.append(train_recall)

train_f1_scores.append(train_f1)

train_fpr_scores.append(train_fpr)

plt.grid(True)

plt.plot([0, 1], [0, 1], 'k--') # 绘制对角线

plt.xlabel('False Positive Rate')

plt.ylabel('True Positive Rate')

plt.title('ROC For Metastasis of LN.prRLN (Training set)')

plt.legend(loc='lower right')

plt.show()

# 打印最佳模型的名称和AUC值

print(f"最佳模型: {best_model_name} with AUC = {best_auc}")

# 使用最佳模型在验证集上进行评估

if hasattr(best_model, 'predict_proba'):

y_val_pred_prob = best_model.predict_proba(class_x_val)[:, 1]

else:

y_val_pred_prob = best_model.decision_function(class_x_val)

# 计算验证集上的AUC值

val_auc = roc_auc_score(class_y_val, y_val_pred_prob)

# 打印验证集上的AUC值

print(f"验证集上的AUC = {val_auc}")

# 创建训练集评价指标的DataFrame

train_metrics_df = pd.DataFrame({

'Model': list(model_param_grid.keys()),

'Accuracy': train_accuracy_scores,

'AUC': train_auc_scores,

'Precision': train_precision_scores,

'Specificity': train_specificity_scores,

'Sensitivity': train_sensitivity_scores,

'Negative Predictive Value': train_npv_scores,

'Positive Predictive Value': train_ppv_scores,

'Recall': train_recall_scores,

'F1 Score': train_f1_scores,

'False Positive Rate': train_fpr_scores

})

# 显示训练集评价指标DataFrame

print(train_metrics_df)

# 将训练集评价指标DataFrame导出为CSV文件

# 将训练集评价指标DataFrame导出为CSV文件

train_metrics_df.to_csv('/Users/zj/Desktop/4.机器学习/三、喉返后/0.数据/结果/2.p分析结果/1.1.1训练集的评价指标.csv', index=False)

#2、DCA

#训练集的决策曲线

import numpy as np

import matplotlib.pyplot as plt

from sklearn.metrics import precision_score

# 定义风险阈值

thresholds = np.linspace(0, 1, 100)

tra_net_benefit = []

for (name, (model, param_grid)), color in zip(model_param_grid.items(), colors):

grid_search = GridSearchCV(estimator=model, param_grid=param_grid, cv=10)

grid_search.fit(class_x_tra, class_y_tra)

best_model_temp = grid_search.best_estimator_

# 计算训练集上的预测概率

if hasattr(best_model_temp, 'predict_proba'):

y_tra_pred_prob = best_model_temp.predict_proba(class_x_tra)[:, 1]

else:

y_tra_pred_prob = best_model_temp.decision_function(class_x_tra)

tra_model_net_benefit = []

# 计算每个阈值下的净收益

for threshold in thresholds:

tra_predictions = (y_tra_pred_prob >= threshold).astype(int)

tra_net_benefit_value = (precision_score(class_y_tra, tra_predictions) - threshold * (1 - precision_score(class_y_tra, tra_predictions))) / (threshold + 1e-10)

tra_model_net_benefit.append(tra_net_benefit_value)

tra_net_benefit.append(tra_model_net_benefit)

# 转换为数组

tra_net_benefit = np.array(tra_net_benefit)

# 计算所有人都进行干预时的净收益

tra_all_predictions = np.ones_like(class_y_tra) # 将所有预测标记为阳性（正类）

tra_all_net_benefit = (precision_score(class_y_tra, tra_all_predictions) - thresholds * (1 - precision_score(class_y_tra, tra_all_predictions))) / (thresholds + 1e-10)

names = [

'Logistic Regression',

'Decision Tree',

'Random Forest',

'Gradient Boosting',

'Support Vector Machine',

'K-Nearest Neighbors',

'Gaussian Naive Bayes',

'Neural Network',

'XGBoost',

]

# 绘制DCA曲线

for i in range(tra_net_benefit.shape[0]):

plt.plot(thresholds, tra_net_benefit[i], color=colors[i], label=names[i])

# 绘制"None"和"All"线

plt.plot(thresholds, np.zeros_like(thresholds), color='black', linestyle='-', label='None')

plt.plot(thresholds, tra_all_net_benefit, color='gray', linestyle='--', label='All')

# 设置y轴的限制

plt.xlim(0, 0.6)

plt.ylim(-0.5,6)

# 设置图形属性

plt.xlabel('Threshold')

plt.ylabel('Net Benefit')

plt.title('Decision Curve Analysis For Metastasis of LN.prRLN (Training set)')

plt.legend(loc='upper right')

# 设置背景灰色格子线

plt.grid(color='lightgray', linestyle='-', linewidth=1)

# 显示图形

plt.show()

#3、校准

#训练集的校准曲线

from sklearn.calibration import calibration_curve

from sklearn.metrics import brier_score_loss

from scipy.stats import ttest_ind

# 创建一个空列表来存储每个模型的校准曲线和Brier Score

train_calibration_curves = []

train_brier_scores = []

# 对每个模型进行循环

for (name, (model, param_grid)), color in zip(model_param_grid.items(), colors):

grid_search = GridSearchCV(estimator=model, param_grid=param_grid, cv=10)

grid_search.fit(class_x_tra, class_y_tra)

best_model_temp = grid_search.best_estimator_

# 计算训练集上的预测概率

if hasattr(best_model_temp, 'predict_proba'):

y_train_pred_prob = best_model_temp.predict_proba(class_x_tra)[:, 1]

else:

y_train_pred_prob = best_model_temp.decision_function(class_x_tra)

# 计算校准曲线

train_fraction_of_positives, train_mean_predicted_value = calibration_curve(class_y_tra, y_train_pred_prob, n_bins=10)

train_calibration_curves.append((train_fraction_of_positives, train_mean_predicted_value, name, color))

# 计算Brier分数

train_brier_score = brier_score_loss(class_y_tra, y_train_pred_prob)

train_brier_scores.append((name, train_brier_score))

# 打印Brier分数

print(f'{name} - Training Brier Score: {train_brier_score:.3f}')

# 绘制校准曲线和Brier Score

fig, ax1 = plt.subplots(figsize=(10, 6))

for curve in train_calibration_curves:

train_fraction_of_positives, train_mean_predicted_value, name, color = curve

# 获取对应模型的Brier Score

train_brier_score = next((score for model, score in train_brier_scores if model == name), None)

# 将Brier Score赋予线颜色标注名称的后面

if train_brier_score is not None:

name += f' (Training Brier Score: {train_brier_score:.3f})'

ax1.plot(train_mean_predicted_value, train_fraction_of_positives, "s-", label=name, color=color)

# 绘制"Perfectly calibrated"曲线

ax1.plot([0, 1], [0, 1], "k:",label="Perfectly calibrated")

ax1.set_ylabel("Fraction of positives")

ax1.set_xlabel("Mean predicted value")

ax1.set_ylim([-0.05, 1.05])

ax1.legend(loc="lower right")

# 设置背景灰色格子线

plt.grid(color='lightgray', linestyle='-', linewidth=1)

plt.title("Calibration Curves For Metastasis of LN.prRLN (Training set)")

plt.tight_layout()

plt.show()

#4、精确召回

#训练集的精确召回曲线

from sklearn.metrics import precision_recall_curve, average_precision_score

import numpy as np

# 初始化存储精确召回曲线和平均精确度的列表

train_precision_recall_curves = []

train_average_precision_scores = []

# 遍历每个模型

for (name, (model, param_grid)), color in zip(model_param_grid.items(), colors):

grid_search = GridSearchCV(estimator=model, param_grid=param_grid, cv=5)

grid_search.fit(class_x_tra, class_y_tra)

best_model_temp = grid_search.best_estimator_

# 计算训练集上的预测概率

if hasattr(best_model_temp, 'predict_proba'):

y_train_pred_prob = best_model_temp.predict_proba(class_x_tra)[:, 1]

else:

y_train_pred_prob = best_model_temp.decision_function(class_x_tra)

# 计算精确召回曲线

train_precision, train_recall, _ = precision_recall_curve(class_y_tra, y_train_pred_prob)

train_average_precision = average_precision_score(class_y_tra, y_train_pred_prob)

# 存储结果

train_precision_recall_curves.append((train_precision, train_recall, f'{name} (AUPR: {train_average_precision:.3f})', color))

train_average_precision_scores.append((f'{name} (AUPR: {train_average_precision:.3f})', train_average_precision))

# 打印平均精确度

print(f'{name} - Training Average Precision: {train_average_precision:.3f}')

# 绘制精确召回曲线

fig, ax2 = plt.subplots(figsize=(10, 6))

for curve in train_precision_recall_curves:

train_precision, train_recall, name, color = curve

ax2.plot(train_recall, train_precision, "-", color=color, label=name)

# 添加随机猜测曲线

plt.plot([0, 1], [class_y_tra.mean(), class_y_tra.mean()], linestyle='--', color='black', label='Random Guessing')

ax2.set_xlabel("Recall")

ax2.set_ylabel("Precision")

ax2.set_ylim([0.0, 1.05])

ax2.set_xlim([0.0, 1.0])

ax2.legend(loc="lower left")

ax2.grid(True)

# 设置背景灰色格子线

plt.grid(color='lightgray', linestyle='-', linewidth=1)

plt.title("Precision-Recall Curves For Metastasis of LN.prRLN (Training set)")

plt.tight_layout()

plt.show()

## 4.2.Val.Development and optimization of prediction

#1.内验证集的roc曲线

import pandas as pd

import matplotlib.pyplot as plt

from sklearn.model_selection import train_test_split, GridSearchCV

from sklearn.metrics import roc_auc_score, roc_curve, accuracy_score, precision_score, recall_score, f1_score, confusion_matrix

from sklearn.linear_model import LogisticRegression

from sklearn.tree import DecisionTreeClassifier

from sklearn.ensemble import RandomForestClassifier, GradientBoostingClassifier

from sklearn.svm import SVC

from sklearn.neighbors import KNeighborsClassifier

from sklearn.naive_bayes import GaussianNB

from sklearn.neural_network import MLPClassifier

from sklearn.preprocessing import MinMaxScaler

import numpy as np

from xgboost import XGBClassifier

from sklearn.model_selection import cross_val_score

# 加载数据

data = pd.read_csv('/Users/zj/Desktop/4.机器学习/三、喉返后/0.数据/1.总P矫正.csv')

# 导入数据

data_feature = data[['Location',

'age','pretracheal.NLNM','pretracheal.LNMR',

'IPNLNM','TCLNMR',]]

data_target = data['LN.prRLNM']

# 数值变量标准化

data_featureNum = data[['age','pretracheal.NLNM','pretracheal.LNMR',

'IPNLNM','TCLNMR',]]

scaler = MinMaxScaler()

data_featureNum = scaler.fit_transform(data_featureNum)

data_featureCata = data[['Location',]]

data_featureCata = np.array(data_featureCata)

# 整合数据

data_feature = np.hstack((data_featureCata, data_featureNum))

# 分为训练集和验证集

class_x_tra, class_x_val, class_y_tra, class_y_val = train_test_split(data_feature, data_target, test_size=0.2, random_state=0)

# 定义模型和参数空间

model_param_grid= {

'Logistic Regression': (LogisticRegression(), {'C': [0.01, 0.1, 1, 10, 100]}),

'Decision Tree': (DecisionTreeClassifier(), {'max_depth': [3, 5, 7, 10], 'min_samples_split': [2, 5, 10]}),

'Random Forest': (RandomForestClassifier(), {'n_estimators': [50, 100, 200], 'max_depth': [3, 5, 7], 'min_samples_split': [2, 5, 10]}),

'Gradient Boosting': (GradientBoostingClassifier(), {'n_estimators': [50, 100, 200], 'learning_rate': [0.01, 0.1, 0.2], 'max_depth': [3, 5, 7]}),

'Support Vector Machine': (SVC(probability=True), {'C': [0.01, 0.1, 1, 10, 100], 'kernel': ['linear', 'rbf']}),

'K-Nearest Neighbors': (KNeighborsClassifier(), {'n_neighbors': [3, 5, 7]}),

'Gaussian Naive Bayes': (GaussianNB(), {}),

'Neural Network': (MLPClassifier(), {'hidden_layer_sizes': [(10,), (20,), (30,)], 'activation': ['relu', 'tanh'], 'solver': ['adam', 'sgd'], 'alpha': [0.0001, 0.001, 0.01], 'learning_rate': ['constant', 'adaptive'], 'learning_rate_init': [0.001, 0.01, 0.1]}),

'XGBoost': (XGBClassifier(use_label_encoder=False, eval_metric='auc'), {'n_estimators': [50, 100, 500], 'colsample_bytree': [0, 1, 11],

'max_depth': [5, 7, 10], 'learning_rate': [0.01,0.05, 0.1], 'subsample': [0.7, 0.8, 0.9], 'gamma': [0, 0.1, 0.5]})

}

# 定义颜色列表

colors = ['blue', 'green', 'red', 'purple', 'brown', 'pink', 'gray', 'cyan','orange']

# 初始化最佳AUC和最佳模型

best_auc = 0

best_model_name = ''

best_model = None

# 创建评价指标的空列表

Val_accuracy_scores = []

Val_auc_scores = []

Val_precision_scores = []

Val_specificity_scores = []

Val_sensitivity_scores = []

Val_npv_scores = []

Val_ppv_scores = []

Val_recall_scores = []

Val_f1_scores = []

Val_fpr_scores = []

# 拟合模型并绘制ROC曲线

plt.figure(figsize=(8, 6))

for (name, (model, param_grid)), color in zip(model_param_grid.items(), colors):

grid_search = GridSearchCV(estimator=model, param_grid=param_grid, cv=2)

grid_search.fit(class_x_tra, class_y_tra, )

best_model_temp = grid_search.best_estimator_

# 计算验证集上的预测概率

if hasattr(best_model_temp, 'predict_proba'):

y_Val_pred_prob = best_model_temp.predict_proba(class_x_val)[:, 1]

else:

y_Val_pred_prob = best_model_temp.decision_function(class_x_val)

# 计算AUC值

auc = roc_auc_score(class_y_val, y_Val_pred_prob)

# 如果当前模型的AUC值是最高的，则更新最佳模型和最佳AUC

if auc > best_auc:

best_auc = auc

best_model_name = name

best_model = best_model_temp

# 计算ROC曲线

fpr, tpr, _ = roc_curve(class_y_val, y_Val_pred_prob)

# 绘制ROC曲线

plt.plot(fpr, tpr, color=color, label='%s (AUC = %0.3f)' % (name, auc))

# 计算其他评价指标

Val_y_pred = best_model_temp.predict(class_x_val)

Val_accuracy = accuracy_score(class_y_val, Val_y_pred)

Val_precision = precision_score(class_y_val, Val_y_pred)

Val_cm = confusion_matrix(class_y_val, Val_y_pred)

Val_tn, Val_fp, Val_fn, Val_tp = Val_cm.ravel()

Val_specificity = Val_tn / (Val_tn + Val_fp)

Val_sensitivity = recall_score(class_y_val, Val_y_pred)

Val_npv = Val_tn / (Val_tn + Val_fn)

Val_ppv = Val_tp / (Val_tp + Val_fp)

Val_recall = Val_sensitivity

Val_f1 = f1_score(class_y_val, Val_y_pred)

Val_fpr = Val_fp / (Val_fp + Val_tn)

# 将评价指标添加到列表中

Val_accuracy_scores.append(Val_accuracy)

Val_auc_scores.append(auc)

Val_precision_scores.append(Val_precision)

Val_specificity_scores.append(Val_specificity)

Val_sensitivity_scores.append(Val_sensitivity)

Val_npv_scores.append(Val_npv)

Val_ppv_scores.append(Val_ppv)

Val_recall_scores.append(Val_recall)

Val_f1_scores.append(Val_f1)

Val_fpr_scores.append(Val_fpr)

plt.grid(True)

plt.plot([0, 1], [0, 1], 'k--') # 绘制对角线

# 设置背景灰色格子线

plt.grid(color='lightgray', linestyle='-', linewidth=1)

plt.xlabel('False Positive Rate')

plt.ylabel('True Positive Rate')

plt.title('ROC For Metastasis of LN.prRLN (Validation set)')

plt.legend(loc='lower right')

plt.show()

# 打印最佳模型的名称和AUC值

print(f"最佳模型: {best_model_name} with AUC = {best_auc}")

# 创建训练集评价指标的DataFrame

Val_metrics_df = pd.DataFrame({

'Model': list(model_param_grid.keys()),

'Accuracy': Val_accuracy_scores,

'AUC': Val_auc_scores,

'Precision': Val_precision_scores,

'Specificity': Val_specificity_scores,

'Sensitivity': Val_sensitivity_scores,

'Negative Predictive Value': Val_npv_scores,

'Positive Predictive Value': Val_ppv_scores,

'Recall': Val_recall_scores,

'F1 Score': Val_f1_scores,

'False Positive Rate': Val_fpr_scores

})

# 显示训练集评价指标DataFrame

print(Val_metrics_df)

# 将训练集评价指标DataFrame导出为CSV文件

Val_metrics_df.to_csv('/Users/zj/Desktop/4.机器学习/三、喉返后/0.数据/结果/2.p分析结果/3.筛选最佳模型/2.1.1验证集的评价指标.csv', index=False)

#2、内验证集的决策曲线

import numpy as np

import matplotlib.pyplot as plt

from sklearn.metrics import precision_score

# 定义风险阈值

thresholds = np.linspace(0, 1, 100)

val_net_benefit = []

for (name, (model, param_grid)), color in zip(model_param_grid.items(), colors):

grid_search = GridSearchCV(estimator=model, param_grid=param_grid, cv=10)

grid_search.fit(class_x_tra, class_y_tra)

best_model_temp = grid_search.best_estimator_

# 计算训练集上的预测概率

if hasattr(best_model_temp, 'predict_proba'):

y_val_pred_prob = best_model_temp.predict_proba(class_x_val)[:, 1]

else:

y_val_pred_prob = best_model_temp.decision_function(class_x_val)

val_model_net_benefit = []

# 计算每个阈值下的净收益

for threshold in thresholds:

val_predictions = (y_val_pred_prob >= threshold).astype(int)

val_net_benefit_value = (precision_score(class_y_val, val_predictions) - threshold * (1 - precision_score(class_y_val, val_predictions))) / (threshold + 1e-10)

val_model_net_benefit.append(val_net_benefit_value)

val_net_benefit.append(val_model_net_benefit)

# 转换为数组

val_net_benefit = np.array(val_net_benefit)

# 计算所有人都进行干预时的净收益

val_all_predictions = np.ones_like(class_y_val) # 将所有预测标记为阳性（正类）

val_all_net_benefit = (precision_score(class_y_val, val_all_predictions) - thresholds * (1 - precision_score(class_y_val, val_all_predictions))) / (thresholds + 1e-10)

names = [

'Logistic Regression',

'Decision Tree',

'Random Forest',

'Gradient Boosting',

'Support Vector Machine',

'K-Nearest Neighbors',

'Gaussian Naive Bayes',

'Neural Network',

'XGBoost'

]

# 绘制DCA曲线

for i in range(val_net_benefit.shape[0]):

plt.plot(thresholds, val_net_benefit[i], color=colors[i], label=names[i])

# 绘制"None"和"All"线

plt.plot(thresholds, np.zeros_like(thresholds), color='black', linestyle='-', label='None')

plt.plot(thresholds, val_all_net_benefit, color='gray', linestyle='--', label='All')

# 设置背景灰色格子线

plt.grid(color='lightgray', linestyle='-', linewidth=1)

# 设置y轴的限制

plt.xlim(0, 0.6)

plt.ylim(-0.5,6)

# 设置图形属性

plt.xlabel('Threshold')

plt.ylabel('Net Benefit')

plt.title('Decision Curve Analysis For Metastasis of LN.prRLN (Validation set)')

plt.legend(loc='upper right')

# 显示图形

plt.show()

#内验证集的校准曲线

from sklearn.calibration import calibration_curve

from sklearn.metrics import brier_score_loss

from scipy.stats import ttest_ind

# 创建一个空列表来存储每个模型的校准曲线和Brier Score

val_calibration_curves = []

val_brier_scores = []

# 对每个模型进行循环

for (name, (model, param_grid)), color in zip(model_param_grid.items(), colors):

grid_search = GridSearchCV(estimator=model, param_grid=param_grid, cv=10)

grid_search.fit(class_x_tra, class_y_tra)

best_model_temp = grid_search.best_estimator_

# 计算训练集上的预测概率

if hasattr(best_model_temp, 'predict_proba'):

y_val_pred_prob = best_model_temp.predict_proba(class_x_val)[:, 1]

else:

y_val_pred_prob = best_model_temp.decision_function(class_x_val)

# 计算校准曲线

val_fraction_of_positives, val_mean_predicted_value = calibration_curve(class_y_val, y_val_pred_prob, n_bins=10)

val_calibration_curves.append((val_fraction_of_positives, val_mean_predicted_value, name, color))

# 计算Brier分数

val_brier_score = brier_score_loss(class_y_val, y_val_pred_prob)

val_brier_scores.append((name, val_brier_score))

# 打印Brier分数

print(f'{name} - Brier Score: {val_brier_score:.3f}')

# 绘制校准曲线和Brier Score

fig, ax1 = plt.subplots(figsize=(10, 6))

for curve in val_calibration_curves:

val_fraction_of_positives, val_mean_predicted_value, name, color = curve

# 获取对应模型的Brier Score

val_brier_score = next((score for model, score in val_brier_scores if model == name), None)

# 将Brier Score赋予线颜色标注名称的后面

if val_brier_score is not None:

name += f' (Brier Score: {val_brier_score:.3f})'

ax1.plot(val_mean_predicted_value, val_fraction_of_positives, "s-", label=name, color=color)

# 绘制"Perfectly calibrated"曲线

ax1.plot([0, 1], [0, 1], "k:",label="Perfectly calibrated")

ax1.set_ylabel("Fraction of positives")

ax1.set_xlabel("Mean predicted value")

ax1.set_ylim([-0.05, 1.05])

ax1.legend(loc="lower right")

# 设置背景灰色格子线

plt.grid(color='lightgray', linestyle='-', linewidth=1)

plt.title("Calibration Curves For Metastasis of LN.prRLN (Validation set)")

plt.tight_layout()

plt.show()

#4、内验证集的精确召回曲线

from sklearn.metrics import precision_recall_curve, average_precision_score

import numpy as np

# 初始化存储精确召回曲线和平均精确度的列表

val_precision_recall_curves = []

val_average_precision_scores = []

# 遍历每个模型

for (name, (model, param_grid)), color in zip(model_param_grid.items(), colors):

grid_search = GridSearchCV(estimator=model, param_grid=param_grid, cv=5)

grid_search.fit(class_x_tra, class_y_tra)

best_model_temp = grid_search.best_estimator_

# 计算训练集上的预测概率

if hasattr(best_model_temp, 'predict_proba'):

y_val_pred_prob = best_model_temp.predict_proba(class_x_val)[:, 1]

else:

y_val_pred_prob = best_model_temp.decision_function(class_x_val)

# 计算精确召回曲线

val_precision, val_recall, _ = precision_recall_curve(class_y_val, y_val_pred_prob)

val_average_precision = average_precision_score(class_y_val, y_val_pred_prob)

# 存储结果

val_precision_recall_curves.append((val_precision, val_recall, f'{name} (AUPR: {val_average_precision:.3f})', color))

val_average_precision_scores.append((f'{name} (AUPR: {val_average_precision:.3f})', val_average_precision))

# 打印平均精确度

print(f'{name} - Average Precision: {val_average_precision:.3f}')

# 绘制精确召回曲线

fig, ax2 = plt.subplots(figsize=(10, 6))

for curve in val_precision_recall_curves:

val_precision, val_recall, name, color = curve

ax2.plot(val_recall, val_precision, "-", color=color, label=name)

# 添加随机猜测曲线

plt.plot([0, 1], [class_y_val.mean(), class_y_val.mean()], linestyle='--', color='black', label='Random Guessing')

ax2.set_xlabel("Recall")

ax2.set_ylabel("Precision")

ax2.set_ylim([0.0, 1.05])

ax2.set_xlim([0.0, 1.0])

ax2.legend(loc="lower left")

ax2.grid(True)

# 设置背景灰色格子线

plt.grid(color='lightgray', linestyle='-', linewidth=1)

plt.title("Precision-Recall Curves For Metastasis of LN.prRLN (Validation set)")

plt.tight_layout()

plt.show()

## 4.3.Test.Development and optimization of prediction models.py

#外验证的roc曲线

import pandas as pd

import matplotlib.pyplot as plt

from sklearn.model_selection import train_test_split, GridSearchCV

from sklearn.metrics import roc_auc_score, roc_curve, accuracy_score, precision_score, recall_score, f1_score, confusion_matrix

from sklearn.linear_model import LogisticRegression

from sklearn.tree import DecisionTreeClassifier

from sklearn.ensemble import RandomForestClassifier, GradientBoostingClassifier

from sklearn.svm import SVC

from sklearn.neighbors import KNeighborsClassifier

from sklearn.naive_bayes import GaussianNB

from sklearn.neural_network import MLPClassifier

from sklearn.preprocessing import MinMaxScaler

import numpy as np

from xgboost import XGBClassifier

from sklearn.model_selection import cross_val_score

# 加载数据

data = pd.read_csv('/Users/zj/Desktop/4.机器学习/三、喉返后/1.矫正后.csv')

# 导入数据

data_feature = data[['prelaryngeal.NLNM','prelaryngeal.LNMR',

'Aspect.ratio','Internal.echo.homogeneous','Ingredients','T.stage','IPNLNM',

]]

data_target = data['LN.prRLNM']

# 数值变量标准化

data_featureNum = data[['prelaryngeal.NLNM','prelaryngeal.LNMR','T.stage','IPNLNM',]]

scaler = MinMaxScaler()

data_featureNum = scaler.fit_transform(data_featureNum)

data_featureCata = data[['Aspect.ratio','Internal.echo.homogeneous','Ingredients',]]

data_featureCata = np.array(data_featureCata)

# 整合数据

data_feature = np.hstack((data_featureCata, data_featureNum))

# 分为训练集和验证集

class_x_tra, class_x_val, class_y_tra, class_y_val = train_test_split(data_feature, data_target, test_size=0.2, random_state=0)

# 定义模型和参数空间

model_param_grid= {

'Logistic Regression': (LogisticRegression(), {'C': [0.01, 0.1, 1, 10, 100]}),

'Decision Tree': (DecisionTreeClassifier(), {'max_depth': [3, 5, 7, 10], 'min_samples_split': [2, 5, 10]}),

'Random Forest': (RandomForestClassifier(), {'n_estimators': [50, 100, 200], 'max_depth': [3, 5, 7], 'min_samples_split': [2, 5, 10]}),

'Gradient Boosting': (GradientBoostingClassifier(), {'n_estimators': [50, 100, 200], 'learning_rate': [0.01, 0.1, 0.2], 'max_depth': [3, 5, 7]}),

'Support Vector Machine': (SVC(probability=True), {'C': [0.01, 0.1, 1, 10, 100], 'kernel': ['linear', 'rbf']}),

'K-Nearest Neighbors': (KNeighborsClassifier(), {'n_neighbors': [3, 5, 7]}),

'Gaussian Naive Bayes': (GaussianNB(), {}),

'Neural Network': (MLPClassifier(), {'hidden_layer_sizes': [(10,), (20,), (30,)], 'activation': ['relu', 'tanh'], 'solver': ['adam', 'sgd'], 'alpha': [0.0001, 0.001, 0.01], 'learning_rate': ['constant', 'adaptive'], 'learning_rate_init': [0.001, 0.01, 0.1]}),

'XGBoost': (XGBClassifier(use_label_encoder=False, eval_metric='auc'), {'n_estimators': [50, 100, 200],

'max_depth': [5, 7, 10], 'learning_rate': [0.01,0.05, 0.1], 'subsample': [0.7, 0.8, 0.9], 'gamma': [0, 0.1, 0.5]})

}

# 定义颜色列表

colors = ['blue', 'green', 'red', 'purple', 'brown', 'pink', 'gray', 'cyan','orange']

# 初始化最佳AUC和最佳模型

best_auc = 0

best_model_name = ''

best_model = None

# Load the external validation set

external_data = pd.read_csv('/Users/zj/Desktop/4.机器学习/三、喉返后/0.数据/2.T总P矫正.csv')

#导入数据

external_feature = external_data[['prelaryngeal.NLNM','prelaryngeal.LNMR','IPNLNM',

'Aspect.ratio','Internal.echo.homogeneous','Ingredients','T.stage',

]]

external_target=external_data['LN.prRLNM']

external_target.unique()#二分类

# Preprocess the external validation set

external_featureCata = external_data[['Aspect.ratio','Internal.echo.homogeneous','Ingredients',]]

external_featureNum = external_data[['prelaryngeal.NLNM','prelaryngeal.LNMR','T.stage','IPNLNM',

]]

external_featureNum = scaler.transform(external_featureNum)

external_feature = np.hstack((external_featureCata, external_featureNum))

external_target = external_data['LN.prRLNM']

# Lists for evaluation metrics

Ext_accuracy_scores = []

Ext_auc_scores = []

Ext_precision_scores = []

Ext_specificity_scores = []

Ext_sensitivity_scores = []

Ext_npv_scores = []

Ext_ppv_scores = []

Ext_recall_scores = []

Ext_f1_scores = []

Ext_fpr_scores = []

# Fit models and plot ROC curve for external validation set

plt.figure(figsize=(8, 6))

for (name, (model, param_grid)), color in zip(model_param_grid.items(), colors):

grid_search = GridSearchCV(estimator=model, param_grid=param_grid, cv=2)

grid_search.fit(class_x_tra, class_y_tra)

best_model_temp = grid_search.best_estimator_

# Predict probabilities on external validation set

if hasattr(best_model_temp, 'predict_proba'):

y_test_pred_prob = best_model_temp.predict_proba(external_feature)[:, 1]

else:

y_test_pred_prob = best_model_temp.decision_function(external_feature)

# Calculate AUC

auc = roc_auc_score(external_target, y_test_pred_prob)

# Update best model if current model has higher AUC

if auc > best_auc:

best_auc = auc

best_model_name = name

best_model = best_model_temp

# Calculate ROC curve

fpr, tpr, _ = roc_curve(external_target, y_test_pred_prob)

# Plot ROC curve

plt.plot(fpr, tpr, color=color, label='%s (AUC = %0.3f)' % (name, auc))

# Calculate other evaluation metrics

y_test_pred = best_model_temp.predict(external_feature)

Ext_accuracy = accuracy_score(external_target, y_test_pred)

Ext_precision = precision_score(external_target, y_test_pred)

Ext_cm = confusion_matrix(external_target, y_test_pred)

Ext_tn, Ext_fp, Ext_fn, Ext_tp = Ext_cm.ravel()

Ext_specificity = Ext_tn / (Ext_tn + Ext_fp)

Ext_sensitivity = recall_score(external_target, y_test_pred)

Ext_npv = Ext_tn / (Ext_tn + Ext_fn)

Ext_ppv = Ext_tp / (Ext_tp + Ext_fp)

Ext_recall = Ext_sensitivity

Ext_f1 = f1_score(external_target, y_test_pred)

Ext_fpr = Ext_fp / (Ext_fp + Ext_tn)

# Append evaluation metrics to lists

Ext_accuracy_scores.append(Ext_accuracy)

Ext_auc_scores.append(auc)

Ext_precision_scores.append(Ext_precision)

Ext_specificity_scores.append(Ext_specificity)

Ext_sensitivity_scores.append(Ext_sensitivity)

Ext_npv_scores.append(Ext_npv)

Ext_ppv_scores.append(Ext_ppv)

Ext_recall_scores.append(Ext_recall)

Ext_f1_scores.append(Ext_f1)

Ext_fpr_scores.append(Ext_fpr)

plt.grid(True)

plt.plot([0, 1], [0, 1], 'k--') # Diagonal line

plt.grid(color='lightgray', linestyle='-', linewidth=1) # Background grid lines

plt.xlabel('False Positive Rate')

plt.ylabel('True Positive Rate')

plt.title('ROC for Metastasis of LN.prRLN (Test Set)')

plt.legend(loc='lower right')

plt.show()

# Print best model name and AUC

print(f"Best model: {best_model_name} with AUC = {best_auc}")

# Create DataFrame for external validation metrics

Ext_metrics_df = pd.DataFrame({

'Model': list(model_param_grid.keys()),

'Accuracy': Ext_accuracy_scores,

'AUC': Ext_auc_scores,

'Precision': Ext_precision_scores,

'Specificity': Ext_specificity_scores,

'Sensitivity': Ext_sensitivity_scores,

'Negative Predictive Value': Ext_npv_scores,

'Positive Predictive Value': Ext_ppv_scores,

'Recall': Ext_recall_scores,

'F1 Score': Ext_f1_scores,

'False Positive Rate': Ext_fpr_scores

})

# Display DataFrame

print(Ext_metrics_df)

# Export metrics to CSV

Ext_metrics_df.to_csv('/Users/zj/Desktop/4.机器学习/三、喉返后/0.数据/结果/2.p分析结果/3.筛选最佳模型/3.1.1测试集的评价指标.csv', index=False)

#2、外验证集的决策曲线

import numpy as np

import matplotlib.pyplot as plt

from sklearn.metrics import precision_score

# 定义风险阈值

thresholds = np.linspace(0, 1, 100)

Ext_net_benefit = []

for (name, (model, param_grid)), color in zip(model_param_grid.items(), colors):

grid_search = GridSearchCV(estimator=model, param_grid=param_grid, cv=5)

grid_search.fit(class_x_tra, class_y_tra)

best_model_temp = grid_search.best_estimator_

# 计算训练集上的预测概率

if hasattr(best_model_temp, 'predict_proba'):

y_Ext_pred_prob = best_model_temp.predict_proba(external_feature)[:, 1]

else:

y_Ext_pred_prob = best_model_temp.decision_function(external_feature)

Ext_model_net_benefit = []

# 计算每个阈值下的净收益

for threshold in thresholds:

Ext_predictions = (y_Ext_pred_prob >= threshold).astype(int)

Ext_net_benefit_value = (precision_score(external_target, Ext_predictions) - threshold * (1 - precision_score(external_target, Ext_predictions))) / (threshold + 1e-10)

Ext_model_net_benefit.append(Ext_net_benefit_value)

Ext_net_benefit.append(Ext_model_net_benefit)

# 转换为数组

Ext_net_benefit = np.array(Ext_net_benefit)

# 计算所有人都进行干预时的净收益

Ext_all_predictions = np.ones_like(external_target) # 将所有预测标记为阳性（正类）

Ext_all_net_benefit = (precision_score(external_target, Ext_all_predictions) - thresholds * (1 - precision_score(external_target, Ext_all_predictions))) / (thresholds + 1e-10)

names = [

'Logistic Regression',

'Decision Tree',

'Random Forest',

'Gradient Boosting',

'Support Vector Machine',

'K-Nearest Neighbors',

'Gaussian Naive Bayes',

'Neural Network',

'XGBoost'

]

# 绘制DCA曲线

for i in range(Ext_net_benefit.shape[0]):

plt.plot(thresholds, Ext_net_benefit[i], color=colors[i], label=names[i])

# 绘制"None"和"All"线

plt.plot(thresholds, np.zeros_like(thresholds), color='black', linestyle='-', label='None')

plt.plot(thresholds, Ext_all_net_benefit, color='gray', linestyle='--', label='All')

# 设置背景灰色格子线

plt.grid(color='lightgray', linestyle='-', linewidth=1)

# 设置y轴的限制

plt.xlim(0, 0.6)

plt.ylim(-0.5,6)

# 设置图形属性

plt.xlabel('Threshold')

plt.ylabel('Net Benefit')

plt.title('Decision Curve Analysis For Metastasis of LN.prRLN (Test set)')

plt.legend(loc='upper right')

# 显示图形

plt.show()

#3、 外验证集的校准曲线

from sklearn.calibration import calibration_curve

from sklearn.metrics import brier_score_loss

from scipy.stats import ttest_ind

# 创建一个空列表来存储每个模型的校准曲线和Brier Score

Ext_calibration_curves = []

Ext_brier_scores = []

# 对每个模型进行循环

for (name, (model, param_grid)), color in zip(model_param_grid.items(), colors):

grid_search = GridSearchCV(estimator=model, param_grid=param_grid, cv=5)

grid_search.fit(class_x_tra, class_y_tra)

best_model_temp = grid_search.best_estimator_

# 计算训练集上的预测概率

if hasattr(best_model_temp, 'predict_proba'):

y_Ext_pred_prob = best_model_temp.predict_proba(external_feature)[:, 1]

else:

y_Ext_pred_prob = best_model_temp.decision_function(external_feature)

# 计算校准曲线

Ext_fraction_of_positives, Ext_mean_predicted_value = calibration_curve(external_target, y_Ext_pred_prob, n_bins=10)

Ext_calibration_curves.append((Ext_fraction_of_positives, Ext_mean_predicted_value, name, color))

# 计算Brier分数

Ext_brier_score = brier_score_loss(external_target, y_Ext_pred_prob)

Ext_brier_scores.append((name, Ext_brier_score))

# 打印Brier分数

print(f'{name} - Brier Score: {Ext_brier_score:.3f}')

# 绘制校准曲线和Brier Score

fig, ax1 = plt.subplots(figsize=(10, 6))

for curve in Ext_calibration_curves:

Ext_fraction_of_positives, Ext_mean_predicted_value, name, color = curve

# 获取对应模型的Brier Score

Ext_brier_score = next((score for model, score in Ext_brier_scores if model == name), None)

# 将Brier Score赋予线颜色标注名称的后面

if Ext_brier_score is not None:

name += f' (Brier Score: {Ext_brier_score:.3f})'

ax1.plot(Ext_mean_predicted_value, Ext_fraction_of_positives, "s-", label=name, color=color)

# 绘制"Perfectly calibrated"曲线

ax1.plot([0, 1], [0, 1], "k:",label="Perfectly calibrated")

ax1.set_ylabel("Fraction of positives")

ax1.set_xlabel("Mean predicted value")

ax1.set_ylim([-0.05, 1.05])

ax1.legend(loc="lower right")

# 设置背景灰色格子线

plt.grid(color='lightgray', linestyle='-', linewidth=1)

plt.title("Calibration Curves For Metastasis of LN.prRLN (Test set)")

plt.tight_layout()

plt.show()

#4、外验证集的精确召回曲线

from sklearn.metrics import precision_recall_curve, average_precision_score

import numpy as np

# 初始化存储精确召回曲线和平均精确度的列表

Ext_precision_recall_curves = []

Ext_average_precision_scores = []

# 遍历每个模型

for (name, (model, param_grid)), color in zip(model_param_grid.items(), colors):

grid_search = GridSearchCV(estimator=model, param_grid=param_grid, cv=5)

grid_search.fit(class_x_tra, class_y_tra)

best_model_temp = grid_search.best_estimator_

# 计算训练集上的预测概率

if hasattr(best_model_temp, 'predict_proba'):

y_Ext_pred_prob = best_model_temp.predict_proba(external_feature)[:, 1]

else:

y_Ext_pred_prob = best_model_temp.decision_function(external_feature)

# 计算精确召回曲线

Ext_precision, Ext_recall, _ = precision_recall_curve(external_target, y_Ext_pred_prob)

Ext_average_precision = average_precision_score(external_target, y_Ext_pred_prob)

# 存储结果

Ext_precision_recall_curves.append((Ext_precision, Ext_recall, f'{name} (AUPR: {Ext_average_precision:.3f})', color))

Ext_average_precision_scores.append((f'{name} (AUPR: {Ext_average_precision:.3f})', Ext_average_precision))

# 打印平均精确度

print(f'{name} - Average Precision: {Ext_average_precision:.3f}')

# 绘制精确召回曲线

fig, ax2 = plt.subplots(figsize=(10, 6))

for curve in Ext_precision_recall_curves:

Ext_precision, Ext_recall, name, color = curve

ax2.plot(Ext_recall, Ext_precision, "-", color=color, label=name)

# 添加随机猜测曲线

plt.plot([0, 1], [external_target.mean(), external_target.mean()], linestyle='--', color='black', label='Random Guessing')

ax2.set_xlabel("Recall")

ax2.set_ylabel("Precision")

ax2.set_ylim([0.0, 1.05])

ax2.set_xlim([0.0, 1.0])

ax2.legend(loc="lower left")

ax2.grid(True)

# 设置背景灰色格子线

plt.grid(color='lightgray', linestyle='-', linewidth=1)

plt.title("Precision-Recall Curves For Metastasis of LN.prRLN (Test set)")

plt.tight_layout()

plt.show()

## 5.1.SHAP.Parameter setting.py

pip install xgboost

pip install shap

#5.1.1

import pandas as pd

import numpy as np

from sklearn.model_selection import RepeatedKFold, cross_validate

import xgboost

from sklearn.model_selection import GridSearchCV

import matplotlib.pyplot as plt

from sklearn.model_selection import train_test_split

from xgboost import XGBClassifier

from sklearn.model_selection import cross_val_score

df = pd.read_csv('/Users/zj/Desktop/4.机器学习/三、喉返后/1.矫正后_副本.csv')

y = df['LN.prRLNM']

X = df.drop(['LN.prRLNM'], axis=1)

X_train, X_test, y_train, y_test = train_test_split(X, y, test_size=0.3, random_state=2)

other_params = {'eta': 0.025, 'n_estimators': 333, 'gamma': 0.89, 'max_depth': 3, 'min_child_weight': 0,

'colsample_bytree': 0.3, 'colsample_bylevel': 0.0, 'subsample': 0.11111111111, 'reg_lambda': 0.2, 'reg_alpha': 0,}

cv_params = {'n_estimators': np.linspace(0, 3000, 10, dtype=int)}

model = xgboost.XGBClassifier(**other_params)

gs = GridSearchCV(estimator=model, param_grid=cv_params, scoring='roc_auc', cv=10, verbose=1, n_jobs=-1)

gs.fit(X_train, y_train)

print("参数的最佳取值：:", gs.best_params_)

print("最佳模型得分:", gs.best_score_)

#5.1.2

other_params = {'eta': 0.025, 'n_estimators': 333, 'gamma': 0.89, 'max_depth': 3, 'min_child_weight': 0,

'colsample_bytree': 0.3, 'colsample_bylevel': 0.0, 'subsample': 0.11111111111, 'reg_lambda': 0.2, 'reg_alpha': 0,}

cv_params = {'max_depth': np.linspace(1, 10, 15, dtype=int)}

model = xgboost.XGBClassifier(**other_params)

gs = GridSearchCV(estimator=model, param_grid=cv_params, scoring='accuracy', cv=10, verbose=1, n_jobs=-1)

gs.fit(X_train, y_train)

print("参数的最佳取值：:", gs.best_params_)

print("最佳模型得分:", gs.best_score_)

#5.1.3

import pandas as pd

import numpy as np

from sklearn.model_selection import RepeatedKFold, cross_validate

import xgboost

from sklearn.model_selection import GridSearchCV

import matplotlib.pyplot as plt

from sklearn.model_selection import train_test_split

from xgboost import XGBClassifier

from sklearn.model_selection import cross_val_score

df = pd.read_csv('/Users/zj/Desktop/4.机器学习/三、喉返后/1.矫正后_副本.csv')

y = df['LN.prRLNM']

X = df.drop(['LN.prRLNM'], axis=1)

X_train, X_test, y_train, y_test = train_test_split(X, y, test_size=0.3, random_state=2)

other_params = {'eta': 0.025, 'n_estimators': 333, 'gamma': 0.89, 'max_depth': 3, 'min_child_weight': 0,

'colsample_bytree': 0.3, 'colsample_bylevel': 0.0, 'subsample': 0.11111111111, 'reg_lambda': 0.2, 'reg_alpha': 0,}

cv_params = {'min_child_weight': np.linspace(0, 10, 20, dtype=int)}

model = xgboost.XGBClassifier(**other_params)

gs = GridSearchCV(estimator=model, param_grid=cv_params, scoring='accuracy', cv=5, verbose=1, n_jobs=-1)

gs.fit(X_train, y_train)

print("参数的最佳取值：:", gs.best_params_)

print("最佳模型得分:", gs.best_score_)

#5.1.4

import pandas as pd

import numpy as np

from sklearn.model_selection import RepeatedKFold, cross_validate

import xgboost

from sklearn.model_selection import GridSearchCV

import matplotlib.pyplot as plt

from sklearn.model_selection import train_test_split

from xgboost import XGBClassifier

from sklearn.model_selection import cross_val_score

df = pd.read_csv('/Users/zj/Desktop/4.机器学习/三、喉返后/1.矫正后_副本.csv')

y = df['LN.prRLNM']

X = df.drop(['LN.prRLNM'], axis=1)

X_train, X_test, y_train, y_test = train_test_split(X, y, test_size=0.3, random_state=2)

other_params = {'eta': 0.025, 'n_estimators': 333, 'gamma': 0.89, 'max_depth': 3, 'min_child_weight': 0,

'colsample_bytree': 0.3, 'colsample_bylevel': 0.0, 'subsample': 0.11111111111, 'reg_lambda': 0.2, 'reg_alpha': 0,}

cv_params = {'gamma': np.linspace(0.01, 1, 10)}

model = xgboost.XGBClassifier(**other_params)

gs = GridSearchCV(estimator=model, param_grid=cv_params, scoring='accuracy', cv=10, verbose=1, n_jobs=-1)

gs.fit(X_train, y_train)

print("参数的最佳取值：:", gs.best_params_)

print("最佳模型得分:", gs.best_score_)

#5.1.5

import pandas as pd

import numpy as np

from sklearn.model_selection import RepeatedKFold, cross_validate

import xgboost

from sklearn.model_selection import GridSearchCV

import matplotlib.pyplot as plt

from sklearn.model_selection import train_test_split

from xgboost import XGBClassifier

from sklearn.model_selection import cross_val_score

df = pd.read_csv('/Users/zj/Desktop/4.机器学习/三、喉返后/1.矫正后_副本.csv')

y = df['LN.prRLNM']

X = df.drop(['LN.prRLNM'], axis=1)

X_train, X_test, y_train, y_test = train_test_split(X, y, test_size=0.3, random_state=2)

other_params = {'eta': 0.025, 'n_estimators': 333, 'gamma': 0.89, 'max_depth': 3, 'min_child_weight': 0,

'colsample_bytree': 0.3, 'colsample_bylevel': 0.0, 'subsample': 0.11111111111, 'reg_lambda': 0.2, 'reg_alpha': 0,}

cv_params = {'subsample': np.linspace(0, 1, 10)}

model = xgboost.XGBClassifier(**other_params)

gs = GridSearchCV(estimator=model, param_grid=cv_params, scoring='accuracy', cv=10, verbose=1, n_jobs=-1)

gs.fit(X_train, y_train)

print("参数的最佳取值：:", gs.best_params_)

print("最佳模型得分:", gs.best_score_)

#5.1.6

import pandas as pd

import numpy as np

from sklearn.model_selection import RepeatedKFold, cross_validate

import xgboost

from sklearn.model_selection import GridSearchCV

import matplotlib.pyplot as plt

from sklearn.model_selection import train_test_split

from xgboost import XGBClassifier

from sklearn.model_selection import cross_val_score

df = pd.read_csv('/Users/zj/Desktop/4.机器学习/三、喉返后/1.矫正后_副本.csv')

y = df['LN.prRLNM']

X = df.drop(['LN.prRLNM'], axis=1)

X_train, X_test, y_train, y_test = train_test_split(X, y, test_size=0.3, random_state=2)

other_params = {'eta': 0.025, 'n_estimators': 333, 'gamma': 0.89, 'max_depth': 3, 'min_child_weight': 0,

'colsample_bytree': 0.3, 'colsample_bylevel': 0.0, 'subsample': 0.11111111111, 'reg_lambda': 0.2, 'reg_alpha': 0,}

cv_params = {'colsample_bytree': np.linspace(0, 1, 11)}

model = xgboost.XGBClassifier(**other_params)

gs = GridSearchCV(estimator=model, param_grid=cv_params, scoring='accuracy', cv=10, verbose=1, n_jobs=-1)

gs.fit(X_train, y_train)

print("参数的最佳取值：:", gs.best_params_)

print("最佳模型得分:", gs.best_score_)

#5.1.7

import pandas as pd

import numpy as np

from sklearn.model_selection import RepeatedKFold, cross_validate

import xgboost

from sklearn.model_selection import GridSearchCV

import matplotlib.pyplot as plt

from sklearn.model_selection import train_test_split

from xgboost import XGBClassifier

from sklearn.model_selection import cross_val_score

df = pd.read_csv('/Users/zj/Desktop/4.机器学习/三、喉返后/1.矫正后_副本.csv')

y = df['LN.prRLNM']

X = df.drop(['LN.prRLNM'], axis=1)

X_train, X_test, y_train, y_test = train_test_split(X, y, test_size=0.3, random_state=2)

other_params = {'eta': 0.025, 'n_estimators': 333, 'gamma': 0.89, 'max_depth': 3, 'min_child_weight': 0,

'colsample_bytree': 0.3, 'colsample_bylevel': 0.0, 'subsample': 0.11111111111, 'reg_lambda': 0.2, 'reg_alpha': 0,}

cv_params = {'colsample_bylevel': np.linspace(0, 1, 11)}

model = xgboost.XGBClassifier(**other_params)

gs = GridSearchCV(estimator=model, param_grid=cv_params, scoring='accuracy', cv=10, verbose=1, n_jobs=-1)

gs.fit(X_train, y_train)

print("参数的最佳取值：:", gs.best_params_)

print("最佳模型得分:", gs.best_score_)

#5.1.8

import pandas as pd

import numpy as np

from sklearn.model_selection import RepeatedKFold, cross_validate

import xgboost

from sklearn.model_selection import GridSearchCV

import matplotlib.pyplot as plt

from sklearn.model_selection import train_test_split

from xgboost import XGBClassifier

from sklearn.model_selection import cross_val_score

df = pd.read_csv('/Users/zj/Desktop/4.机器学习/三、喉返后/1.矫正后_副本.csv')

y = df['LN.prRLNM']

X = df.drop(['LN.prRLNM'], axis=1)

X_train, X_test, y_train, y_test = train_test_split(X, y, test_size=0.3, random_state=2)

other_params = {'eta': 0.025, 'n_estimators': 333, 'gamma': 0.89, 'max_depth': 3, 'min_child_weight': 0,

'colsample_bytree': 0.3, 'colsample_bylevel': 0.0, 'subsample': 0.11111111111, 'reg_lambda': 0.2, 'reg_alpha': 0,}

cv_params = {'reg_lambda': np.linspace(0, 1, 11)}

model = xgboost.XGBClassifier(**other_params)

gs = GridSearchCV(estimator=model, param_grid=cv_params, scoring='accuracy', cv=10, verbose=1, n_jobs=-1)

gs.fit(X_train, y_train)

print("参数的最佳取值：:", gs.best_params_)

print("最佳模型得分:", gs.best_score_)

#5.1.9

import pandas as pd

import numpy as np

from sklearn.model_selection import RepeatedKFold, cross_validate

import xgboost

from sklearn.model_selection import GridSearchCV

import matplotlib.pyplot as plt

from sklearn.model_selection import train_test_split

from xgboost import XGBClassifier

from sklearn.model_selection import cross_val_score

df = pd.read_csv('/Users/zj/Desktop/4.机器学习/三、喉返后/1.矫正后_副本.csv')

y = df['LN.prRLNM']

X = df.drop(['LN.prRLNM'], axis=1)

X_train, X_test, y_train, y_test = train_test_split(X, y, test_size=0.3, random_state=2)

other_params = {'eta': 0.027825594022071243, 'n_estimators': 333, 'gamma': 0.89, 'max_depth': 3, 'min_child_weight': 0,

'colsample_bytree': 0.3, 'colsample_bylevel': 0.0, 'subsample': 0.11111111111, 'reg_lambda': 0.2, 'reg_alpha': 0}

cv_params = {'reg_alpha': np.linspace(0, 10, 10)}

model = xgboost.XGBClassifier(**other_params)

gs = GridSearchCV(estimator=model, param_grid=cv_params, scoring='accuracy', cv=10, verbose=1, n_jobs=-1)

gs.fit(X_train, y_train)

print("参数的最佳取值：:", gs.best_params_)

print("最佳模型得分:", gs.best_score_)

#5.1.10

import pandas as pd

import numpy as np

from sklearn.model_selection import RepeatedKFold, cross_validate

import xgboost

from sklearn.model_selection import GridSearchCV

import matplotlib.pyplot as plt

from sklearn.model_selection import train_test_split

from xgboost import XGBClassifier

from sklearn.model_selection import cross_val_score

df = pd.read_csv('/Users/zj/Desktop/4.机器学习/三、喉返后/1.矫正后_副本.csv')

y = df['LN.prRLNM']

X = df.drop(['LN.prRLNM'], axis=1)

X_train, X_test, y_train, y_test = train_test_split(X, y, test_size=0.3, random_state=2)

other_params = {'eta': 0.027825594022071243, 'n_estimators': 333, 'gamma': 0.89, 'max_depth': 3, 'min_child_weight': 0,

'colsample_bytree': 0.3, 'colsample_bylevel': 0.0, 'subsample': 0.11111111111, 'reg_lambda': 0.2, 'reg_alpha': 0}

cv_params = {'eta': np.logspace(-2, 0, 10)}

model = xgboost.XGBClassifier(**other_params)

gs = GridSearchCV(estimator=model, param_grid=cv_params, scoring='accuracy', cv=10, verbose=1, n_jobs=-1)

gs.fit(X_train, y_train)

print("参数的最佳取值：:", gs.best_params_)

print("最佳模型得分:", gs.best_score_)

## 5.2.SHAP.Decision curve.py

import pandas as pd

import numpy as np

from sklearn.model_selection import RepeatedKFold, cross_validate

import xgboost

from sklearn.model_selection import GridSearchCV

import matplotlib.pyplot as plt

from sklearn.model_selection import train_test_split

from xgboost import XGBClassifier

from sklearn.model_selection import cross_val_score

import shap

from sklearn.model_selection import KFold

import sklearn

# Load data

df = pd.read_csv('/Users/zj/Desktop/4.机器学习/三、喉返后/1.矫正后_副本.csv')

# Select features and target variable

X = df[['Tumor.border','Hyperechoic','Location','IPLNM','TCLNM','age','size','pretracheal.LNMR',

'pretracheal.NLNM','IPLNMR','IPNLNM','TCLNMR','TCNLNM']]

y = df['LN.prRLNM']

# Split into training and test sets

X_train, X_test, y_train, y_test = train_test_split(X, y, test_size=0.3, random_state=0)

# Define XGBClassifier parameters

params = {'eta': 0.027825594022071243, 'n_estimators': 333, 'gamma': 0.89, 'max_depth': 3, 'min_child_weight': 0,

'colsample_bytree': 0.3, 'colsample_bylevel': 0.0, 'subsample': 0.11111111111, 'reg_lambda': 0.2, 'reg_alpha': 0

}

# Initialize and train the model

model = XGBClassifier(**params)

eval_set = [(X_test, y_test)]

model.fit(X_train, y_train, early_stopping_rounds=10, eval_metric="logloss", eval_set=eval_set, verbose=False)

# Use SHAP to explain the model predictions

explainer = shap.TreeExplainer(model)

# 计算所有样本的SHAP值

shap_values = explainer.shap_values(X)

#条形图和散点图

shap.summary_plot(shap_values, X, plot_type="bar", color="green")

shap.summary_plot(shap_values, X, plot_type="dot")

#分类条形图

ndf =df.sort_values(by="LN.prRLNM")

print(ndf)

X2 = ndf[2:1472].drop(['LN.prRLNM'], axis=1)

X1 = ndf[1473:1713].drop(['LN.prRLNM'], axis=1)

shap_values = explainer.shap_values(X)

shap_values1 = explainer.shap_values(X1[1:224])

shap_values2 = explainer.shap_values(X2[1:224])

shap.summary_plot([shap_values1, shap_values2], X, plot_type="bar", class_names=["non-metastasis","metastasis"])

##因为只纳入了244个数据，所以输出的顺序不一致，需要手动更改，很重要！！！！

# 获取所有特征变量的名称

feature_names = X.columns.tolist()

# 手动指定特征变量名称的顺序

feature_names_order = ['Tumor.border', 'Hyperechoic','Location', 'age','TCNLNM','size','pretracheal.NLNM','IPLNM','TCLNMR','IPNLNM','TCLNM','pretracheal.LNMR','IPLNM',]

shap.summar

import pandas as pd

import numpy as np

from sklearn.model_selection import RepeatedKFold, cross_validate

import xgboost

from sklearn.model_selection import GridSearchCV

import matplotlib.pyplot as plt

from sklearn.model_selection import train_test_split

from xgboost import XGBClassifier

from sklearn.model_selection import cross_val_score

import shap

from sklearn.model_selection import KFold

import sklearn

# Load data

df = pd.read_csv('/Users/zj/Desktop/4.机器学习/三、喉返后/1.矫正后_副本.csv')

# Select features and target variable

X = df[['Tumor.border','Hyperechoic','Location','IPLNM','TCLNM','age','size','pretracheal.LNMR',

'pretracheal.NLNM','IPLNMR','IPNLNM','TCLNMR','TCNLNM']]

y = df['LN.prRLNM']

# Split into training and test sets

X_train, X_test, y_train, y_test = train_test_split(X, y, test_size=0.3, random_state=0)

# Define XGBClassifier parameters

params = {'eta': 0.027825594022071243, 'n_estimators': 333, 'gamma': 0.89, 'max_depth': 3, 'min_child_weight': 0,

'colsample_bytree': 0.3, 'colsample_bylevel': 0.0, 'subsample': 0.11111111111, 'reg_lambda': 0.2, 'reg_alpha': 0

}

# Initialize and train the model

model = XGBClassifier(**params)

eval_set = [(X_test, y_test)]

model.fit(X_train, y_train, early_stopping_rounds=10, eval_metric="logloss", eval_set=eval_set, verbose=False)

# Use SHAP to explain the model predictions

explainer = shap.TreeExplainer(model)

shap_values = explainer.shap_values(X)

shap.decision_plot(explainer.expected_value, shap_values[730, :], X.iloc[730, :], link='logit')

shap.bar_plot(shap_values[730, :], feature_names=X.columns)

shap.decision_plot(explainer.expected_value, shap_values, X, link='logit')

shap.decision_plot(explainer.expected_value, shap_values, X, feature_order="hclust")

shap.plots.force(explainer.expected_value, shap_values[730, :], X.iloc[730, :], link="logit", matplotlib=True)

###最终采用的这个版本以下所见为重要，以上为练习

import shap

# 计算所有样本的SHAP值

shap_values = explainer.shap_values(X)

# 计算每个特征的平均绝对SHAP值

mean_abs_shap = np.mean(np.abs(shap_values), axis=0)

# 按照特征重要性排序

feature_order = np.argsort(mean_abs_shap)

# 绘制Decision Plot

shap.decision_plot(explainer.expected_value, shap_values, X, feature_order=feature_order)

import pandas as pd

import numpy as np

from sklearn.model_selection import RepeatedKFold, cross_validate

import xgboost

from sklearn.model_selection import GridSearchCV

import matplotlib.pyplot as plt

from sklearn.model_selection import train_test_split

from xgboost import XGBClassifier

from sklearn.model_selection import cross_val_score

import shap

from sklearn.model_selection import KFold

import sklearn

# Load data

df = pd.read_csv('/Users/zj/Desktop/4.机器学习/三、喉返后/1.矫正后_副本.csv')

# Select features and target variable

X = df[['Tumor.border','Hyperechoic','Location','IPLNM','TCLNM','age','size','pretracheal.LNMR',

'pretracheal.NLNM','IPLNMR','IPNLNM','TCLNMR','TCNLNM']]

y = df['LN.prRLNM']

# Split into training and test sets

X_train, X_test, y_train, y_test = train_test_split(X, y, test_size=0.3, random_state=0)

# Define XGBClassifier parameters

params = {'eta': 0.027825594022071243, 'n_estimators': 333, 'gamma': 0.89, 'max_depth': 3, 'min_child_weight': 0,

'colsample_bytree': 0.3, 'colsample_bylevel': 0.0, 'subsample': 0.11111111111, 'reg_lambda': 0.2, 'reg_alpha': 0

}

cv= KFold(n_splits=10, random_state=0, shuffle=True)

for i, (train, test) in enumerate(cv.split(X_train, y_train)):

model = XGBClassifier(**params).fit(X_train.iloc[train], y_train.iloc[train])

explainer = shap.TreeExplainer(model)

expected_value = explainer.expected_value

select = range(1000)

features = X.iloc[295:315]

features_display = X.loc[features.index]

shap_values = explainer.shap_values(features)

shap.decision_plot(expected_value, shap_values, features_display,feature_order=feature_order)

y_pred = (shap_values.sum(1) + expected_value) > 0

misclassified = y_pred != y[295:315]

shap.decision_plot(expected_value, shap_values, features_display, highlight=misclassified,feature_order=feature_order)

shap.decision_plot(expected_value, shap_values[misclassified], features_display[misclassified],

link='logit', highlight=0,feature_order=feature_order)

## 5.3.SHAP.visualization.py

shap值的可视化热图及绝对值图展示

import pandas as pd

import numpy as np

from sklearn.model_selection import RepeatedKFold, cross_validate

import xgboost

from sklearn.model_selection import GridSearchCV

import matplotlib.pyplot as plt

from sklearn.model_selection import train_test_split

from xgboost import XGBClassifier

from sklearn.model_selection import cross_val_score

import shap

from sklearn.model_selection import KFold

import sklearn

# Load data

df = pd.read_csv('/Users/zj/Desktop/4.机器学习/三、喉返后/1.矫正后_副本.csv')

# Select features and target variable

X = df[['Tumor.border','Hyperechoic','Location','IPLNM','TCLNM','age','size','pretracheal.LNMR',

'pretracheal.NLNM','IPLNMR','IPNLNM','TCLNMR','TCNLNM']]

y = df['LN.prRLNM']

# Split into training and test sets

X_train, X_test, y_train, y_test = train_test_split(X, y, test_size=0.3, random_state=0)

# Define XGBClassifier parameters

params = {'eta': 0.027825594022071243, 'n_estimators': 333, 'gamma': 0.89, 'max_depth': 3, 'min_child_weight': 0,

'colsample_bytree': 0.3, 'colsample_bylevel': 0.0, 'subsample': 0.11111111111, 'reg_lambda': 0.2, 'reg_alpha': 0

}

model = XGBClassifier(**params)

eval_set = [(X_test, y_test)]

model.fit(X_train, y_train, early_stopping_rounds=10, eval_metric="logloss", eval_set=eval_set, verbose=False)

explainer = shap.Explainer(model)

shap_values = explainer(X)

shap.plots.scatter(shap_values[:,'IPLNM'])

shap.plots.scatter(shap_values[:,'IPLNMR'])

shap.plots.scatter(shap_values[:,'TCLNMR'])

shap.plots.scatter(shap_values[:,'IPNLNM'])

shap.plots.scatter(shap_values[:,'pretracheal.NLNM'])

shap.plots.scatter(shap_values[:,'TCNLNM'])

shap.plots.scatter(shap_values[:,'Tumor.border'])

shap.plots.scatter(shap_values[:, 'age'])

shap.plots.scatter(shap_values[:,'pretracheal.LNMR'])

shap.plots.scatter(shap_values[:, 'Location'])

shap.plots.scatter(shap_values[:,'size'])

shap.plots.scatter(shap_values[:,'Hyperechoic'])

shap.plots.heatmap(shap_values[:1715])

shap.plots.bar(shap_values)

shap.plots.bar(shap_values.abs.max(0))

shap.plots.beeswarm(shap_values)

shap.plots.beeswarm(shap_values.abs, color="shap_red")

clustering = shap.utils.hclust(X, y)

shap.plots.bar(shap_values, clustering=clustering)

shap.plots.bar(shap_values, clustering=clustering, clustering_cutoff=0.8)

shap.plots.bar(shap_values, clustering=clustering, clustering_cutoff=1.8)

import pandas as pd

import numpy as np

import matplotlib.pyplot as plt

from xgboost import XGBClassifier

from sklearn.model_selection import train_test_split

import shap

# Load data

df = pd.read_csv('/Users/zj/Desktop/4.机器学习/三、喉返后/1.矫正后_副本.csv')

# Select features and target variable

X = df[['Tumor.border','Hyperechoic','Location','IPLNM','TCLNM','age','size','pretracheal.LNMR',

'pretracheal.NLNM','IPLNMR','IPNLNM','TCLNMR','TCNLNM']]

y = df['LN.prRLNM']

# Split into training and test sets

X_train, X_test, y_train, y_test = train_test_split(X, y, test_size=0.3, random_state=0)

# Define XGBClassifier parameters

params = {'eta': 0.027825594022071243, 'n_estimators': 333, 'gamma': 0.89, 'max_depth': 3, 'min_child_weight': 0,

'colsample_bytree': 0.3, 'colsample_bylevel': 0.0, 'subsample': 0.11111111111, 'reg_lambda': 0.2, 'reg_alpha': 0

}

model = XGBClassifier(**params)

eval_set = [(X_test, y_test)]

model.fit(X_train, y_train, early_stopping_rounds=10, eval_metric="logloss", eval_set=eval_set, verbose=False)

# Calculate SHAP values

explainer = shap.Explainer(model)

shap_values = explainer(X)

# Calculate mean absolute SHAP values for each feature

mean_abs_shap_values = np.mean(np.abs(shap_values.values), axis=0)

feature_names = X.columns

# Create a DataFrame for the feature importances

shap_importance_df = pd.DataFrame({'Feature': feature_names, 'Mean Abs SHAP': mean_abs_shap_values})

# Calculate contribution percentage

shap_importance_df['Contribution (%)'] = (shap_importance_df['Mean Abs SHAP'] / shap_importance_df['Mean Abs SHAP'].sum()) * 100

# Sort the DataFrame by contribution percentage

shap_importance_df = shap_importance_df.sort_values(by='Contribution (%)', ascending=False)

# Plot the feature contributions as a bar chart

plt.figure(figsize=(12, 8))

bars = plt.barh(shap_importance_df['Feature'], shap_importance_df['Contribution (%)'], color='skyblue')

plt.xlabel('Contribution (%)')

plt.ylabel('Feature')

plt.title('Metastasis of LN.prRLN Contribution Percentage based on SHAP values')

plt.gca().invert_yaxis()

# Add percentage values at the end of each bar

for bar, value in zip(bars, shap_importance_df['Contribution (%)']):

plt.text(bar.get_width() + 0.1, bar.get_y() + bar.get_height()/2, f'{value:.3f}%', va='center')

plt.show()

# Display the DataFrame

print(shap_importance_df)

import pandas as pd

import numpy as np

import matplotlib.pyplot as plt

from xgboost import XGBClassifier

from sklearn.model_selection import train_test_split

import shap

# Load data

df = pd.read_csv('/Users/zj/Desktop/4.机器学习/三、喉返后/1.矫正后_副本.csv')

# Select features and target variable

X = df[['Tumor.border','Hyperechoic','Location','IPLNM','TCLNM','age','size','pretracheal.LNMR',

'pretracheal.NLNM','IPLNMR','IPNLNM','TCLNMR','TCNLNM']]

y = df['LN.prRLNM']

# Split into training and test sets

X_train, X_test, y_train, y_test = train_test_split(X, y, test_size=0.3, random_state=0)

# Define XGBClassifier parameters

params = {'eta': 0.027825594022071243, 'n_estimators': 333, 'gamma': 0.89, 'max_depth': 3, 'min_child_weight': 0,

'colsample_bytree': 0.3, 'colsample_bylevel': 0.0, 'subsample': 0.11111111111, 'reg_lambda': 0.2, 'reg_alpha': 0

}

model = XGBClassifier(**params)

eval_set = [(X_test, y_test)]

model.fit(X_train, y_train, early_stopping_rounds=10, eval_metric="logloss", eval_set=eval_set, verbose=False)

# Calculate SHAP values

explainer = shap.Explainer(model)

shap_values = explainer(X)

# Calculate mean absolute SHAP values for each feature

mean_abs_shap_values = np.mean(np.abs(shap_values.values), axis=0)

feature_names = X.columns

# Create a DataFrame for the feature importances

shap_importance_df = pd.DataFrame({'Feature': feature_names, 'Mean Abs SHAP': mean_abs_shap_values})

# Sort the DataFrame by SHAP value and select top 10 features

shap_importance_df = shap_importance_df.sort_values(by='Mean Abs SHAP', ascending=False).head(10)

# Normalize the contribution to sum to 100%

shap_importance_df['Contribution (%)'] = (shap_importance_df['Mean Abs SHAP'] / shap_importance_df['Mean Abs SHAP'].sum()) * 100

# Plot the top 10 feature contributions as a bar chart

plt.figure(figsize=(12, 8))

bars = plt.barh(shap_importance_df['Feature'], shap_importance_df['Contribution (%)'], color='skyblue')

plt.xlabel('Contribution (%)')

plt.ylabel('Feature')

plt.title('Top 10 Metastasis of LN.prRLN Feature Contribution Percentage based on SHAP values')

plt.gca().invert_yaxis()

# Add percentage values at the end of each bar

for bar, value in zip(bars, shap_importance_df['Contribution (%)']):

plt.text(bar.get_width() + 0.5, bar.get_y() + bar.get_height()/2, f'{value:.3f}%', va='center')

plt.show()

# Display the DataFrame

print(shap_importance_df)

import pandas as pd

import numpy as np

import matplotlib.pyplot as plt

from xgboost import XGBClassifier

from sklearn.model_selection import train_test_split

import shap

# Load data

df = pd.read_csv('/Users/zj/Desktop/4.机器学习/三、喉返后/1.矫正后_副本.csv')

# Select features and target variable

X = df[['Tumor.border','Hyperechoic','Location','IPLNM','TCLNM','age','size','pretracheal.LNMR',

'pretracheal.NLNM','IPLNMR','IPNLNM','TCLNMR','TCNLNM']]

y = df['LN.prRLNM']

# Split into training and test sets

X_train, X_test, y_train, y_test = train_test_split(X, y, test_size=0.3, random_state=0)

# Define XGBClassifier parameters

params = {'eta': 0.027825594022071243, 'n_estimators': 333, 'gamma': 0.89, 'max_depth': 3, 'min_child_weight': 0,

'colsample_bytree': 0.3, 'colsample_bylevel': 0.0, 'subsample': 0.11111111111, 'reg_lambda': 0.2, 'reg_alpha': 0

}

model = XGBClassifier(**params)

eval_set = [(X_test, y_test)]

model.fit(X_train, y_train, early_stopping_rounds=10, eval_metric="logloss", eval_set=eval_set, verbose=False)

# Calculate SHAP values

explainer = shap.Explainer(model)

shap_values = explainer(X)

# Calculate mean absolute SHAP values for each feature

mean_abs_shap_values = np.mean(np.abs(shap_values.values), axis=0)

feature_names = X.columns

# Create a DataFrame for the feature importances

shap_importance_df = pd.DataFrame({'Feature': feature_names, 'Mean Abs SHAP': mean_abs_shap_values})

# Sort the DataFrame by SHAP value and select top 5 features

shap_importance_df = shap_importance_df.sort_values(by='Mean Abs SHAP', ascending=False).head(10)

# Normalize the contribution to sum to 100%

shap_importance_df['Contribution (%)'] = (shap_importance_df['Mean Abs SHAP'] / shap_importance_df['Mean Abs SHAP'].sum()) * 100

# Plot the top 5 feature contributions as a pie chart

plt.figure(figsize=(10, 7))

plt.pie(shap_importance_df['Contribution (%)'], labels=shap_importance_df['Feature'], autopct='%1.1f%%', startangle=140, colors=plt.cm.Paired.colors)

plt.title('Top 10 Metastasis of LN.prRLN Feature Contribution Percentage based on SHAP values')

plt.axis('equal') # Equal aspect ratio ensures that pie is drawn as a circle.

plt.show()

# Display the DataFrame

print(shap_importance_df)

## Web page calculator.py

"""

Django settings for hzp project.

Generated by 'django-admin startproject' using Django 3.2.8.

For more information on this file, see

https://docs.djangoproject.com/en/3.2/topics/settings/

For the full list of settings and their values, see

https://docs.djangoproject.com/en/3.2/ref/settings/

"""

import os

from pathlib import Path

# Build paths inside the project like this: BASE_DIR / 'subdir'.

BASE_DIR = Path(__file__).resolve().parent.parent

# Quick-start development settings - unsuitable for production

# See https://docs.djangoproject.com/en/3.2/howto/deployment/checklist/

# SECURITY WARNING: keep the secret key used in production secret!

SECRET_KEY = 'django-insecure-xedt#e)oz!l%eb$x%zr94n(l(*p&o8!i!i30sie%mt=v29k&42'

# SECURITY WARNING: don't run with debug turned on in production!

DEBUG = True

ALLOWED_HOSTS = ['*']

BASE_DIR = os.path.dirname(os.path.dirname(os.path.abspath(__file__)))

STATIC_URL = '/static/' # 通过别名指向STATICFILES_DIRS目录，当然，别名也可以修改

STATIC_ROOT = os.path.join(BASE_DIR, 'static')

STATICFILES_DIRS = [ # 列表或者元组都行

os.path.join(BASE_DIR, 'static') # 你也可以配置多个静态文件目录，只需拼上路径就好了

]

# STATIC_ROOT = os.path.join(BASE_DIR, 'staticfiles')

# STATICFILES_FINDERS = [

# 'django.contrib.staticfiles.finders.FileSystemFinder',

# 'django.contrib.staticfiles.finders.AppDirectoriesFinder',

# ]

TEMPLATES_DIR=os.path.join(BASE_DIR,'templates')

# Application definition

INSTALLED_APPS = [

'django.contrib.admin',

'django.contrib.auth',

'django.contrib.contenttypes',

'django.contrib.sessions',

'django.contrib.messages',

'django.contrib.staticfiles',

'myapp',

'hzp',

]

MIDDLEWARE = [

'django.middleware.security.SecurityMiddleware',

'django.contrib.sessions.middleware.SessionMiddleware',

'django.middleware.common.CommonMiddleware',

'django.middleware.csrf.CsrfViewMiddleware',

'django.contrib.auth.middleware.AuthenticationMiddleware',

'django.contrib.messages.middleware.MessageMiddleware',

'django.middleware.clickjacking.XFrameOptionsMiddleware',

]

ROOT_URLCONF = 'hzp.urls'

TEMPLATES = [

{

'BACKEND': 'django.template.backends.django.DjangoTemplates',

'DIRS': [TEMPLATES_DIR],

'APP_DIRS': True,

'OPTIONS': {

'context_processors': [

'django.template.context_processors.debug',

'django.template.context_processors.request',

'django.contrib.auth.context_processors.auth',

'django.contrib.messages.context_processors.messages',

],

},

},

]

WSGI_APPLICATION = 'hzp.wsgi.application'

# Database

# https://docs.djangoproject.com/en/3.2/ref/settings/#databases

# DATABASES = {

# 'default': {

# 'ENGINE': 'django.db.backends.mysql',

# 'NAME': 'hzp',

# 'HOST': '127.0.0.1',

# 'PORT': '3306',

# 'USER': 'root',

# 'PASSWORD': 'root',

# }

# }

# Password validation

# https://docs.djangoproject.com/en/3.2/ref/settings/#auth-password-validators

AUTH_PASSWORD_VALIDATORS = [

{

'NAME': 'django.contrib.auth.password_validation.UserAttributeSimilarityValidator',

},

{

'NAME': 'django.contrib.auth.password_validation.MinimumLengthValidator',

},

{

'NAME': 'django.contrib.auth.password_validation.CommonPasswordValidator',

},

{

'NAME': 'django.contrib.auth.password_validation.NumericPasswordValidator',

},

]

# Internationalization

# https://docs.djangoproject.com/en/3.2/topics/i18n/

LANGUAGE_CODE = 'en-us'

TIME_ZONE = 'UTC'

USE_I18N = True

USE_L10N = True

USE_TZ = True

# Static files (CSS, JavaScript, Images)

# https://docs.djangoproject.com/en/3.2/howto/static-files/

# STATIC_URL = '/static/'#'/static/'

# Default primary key field type

# https://docs.djangoproject.com/en/3.2/ref/settings/#default-auto-field

DEFAULT_AUTO_FIELD = 'django.db.models.BigAutoField'

import json

from django.core.serializers import serialize

from django.shortcuts import render

from django.utils import timezone

from django.core import serializers

from django.http import HttpResponse

from django.views.decorators.csrf import csrf_exempt

from myapp import models

def index(request):

return render(request, "index.html")

@csrf_exempt

def js1(request):

data = json.loads(request.body)

res=0.04*float(data['value']) + \

0.04*float(data['value2']) + \

0.06*float(data['value3']) + \

0.09*float(data['value4']) + \

0.07*float(data['value5']) + \

0.17*float(data['value7']) + \

0.15*float(data['value8']) + \

0.12*float(data['value10'])

if float(data['value6'])>5:

res=res+0.11

else:

res=res+0.11*float(data['value6'])/5

if float(data['value9'])>5:

res=res+0.15

else:

res=res+0.15*float(data['value9'])/5

return HttpResponse(round(res*100,2)) #round(res*100,2)
